# Supplementary material for: Centering voices of scientists from marginalized backgrounds to understand experiences in climate adaptation science and inform action
Source: PLoS One. 2025 Feb 21;20(2):e0318438. doi: 10.1371/journal.pone.0318438 (PMC11844896; doi:10.1371/journal.pone.0318438)
Supplement: S1 Table — PDF table of Survey questions, formats and answers. (PDF) [file pone.0318438.s001.pdf]

S1 Table: NECASC climate survey questions, question types, and answer options.

Question	Number of respondents	Answer Format	Response Options
**Background and demographics**			
Do you identify as a member of a marginalized or underrepresented group in climate adaptation science?	32	Multiple choice	Yes, No
Please indicate your gender identity	39	Multiple choice	Cisgender Woman, Cisgender Man, Transgender Woman, Transgender Man, Non-binary/Gender non-conforming, Prefer not to respond
Please indicate your racial identity (check as many as apply)	41	Select all that apply	Hispanic/Latino, Black/African/African American, Asian/Asian American, South Asian/South Asian American, Indigenous American/Native American/American Indian/Alaska Native, Native Hawaiian/Pacific Islander, North African/Middle Eastern, White/Caucasian, Mixed Race
Please indicate any disability status that you may have	39	Multiple choice	Yes I have a disability, No I do not have a disability
Do you consider yourself to be	39	Multiple choice	Hetersexual or straight, Gay, Lesbian, Bisexual, Queer, Pansexual, Asexual/Aromantic, Prefer not to disclose
Are you a first generation college student? (your parents did not complete a four-year degree)	39	Multiple choice	Yes, No
Are you the primary caretaker for another family member?	39	Multiple choice	Yes, Partially, No
What is your age group?	39	Multiple choice	18-24 years old, 25-34 years old, 35-44 years old, 45-54 years old, 55-64 years old, 65-74 years old, 75 years and older
Are you currently affiliated with the NE CASC?		Multiple choice	Yes, No
What is/was your role at NE CASC	40	Select all that apply	Fellow (Undergraduate Student), Fellow (Graduate Student), Fellow (Post Doc), Affiliated Investigator (Funded by NE CASC through a competitive project), Staff Member (Federal, University, Tribal Liaisons), Principal Investigator (on 5-year host agreement)

Which NE CASC institution are/were you affiliated with?	40	Multiple choice	University of Massachusetts, Amherst, College of Menominee Nation, Columbia University, Cornell University, Michigan State University, University of Minnesota, University of Missouri, University of Vermont, University of Wisconsin, USFS Northern Research Station, USGS, Woodwell Climate Research Center (Formerly known as Woods Hole)
How long have you been/were you affiliated with NE CASC	39	Multiple choice	Less than 1 year, 1-3 years, 3-5 years, More than 5 years
How many training events and meetings have you attended over the past year (2020 - 2021) that included content about DEIJ issues?	42	Multiple choice	None, More than 1 but less than 3, More than 3 but less than 5, More than 5 but less than 10, More than 10
**Interest and applications of climate adaptation research**			
Why did you choose to pursue climate adaptation research?		Open Answer	NA
How do you hope your climate adaptation research will make an impact?		Open Answer	NA
**How NE CASC addresses the needs of a diverse community**			
NE CASC members with different backgrounds interact well.	52	Likert Scale	Strongly agree (1), Somewhat agree (2), Neither agree nor disagree (3), Somewhat agree (4), Strongly disagree (5)
NE CASC members of different ages are valued equally by the organization.	51	Likert Scale	Strongly agree (1), Somewhat agree (2), Neither agree nor disagree (3), Somewhat agree (4), Strongly disagree (5)
Racial, ethnic, sexual, and gender-based jokes or slurs are not tolerated at this organization.	52	Likert Scale	Strongly agree (1), Somewhat agree (2), Neither agree nor disagree (3), Somewhat agree (4), Strongly disagree (5)
The NE CASC has taken meaningful steps to addressing DEIJ issues over the last year.	52	Likert Scale	Strongly agree (1), Somewhat agree (2), Neither agree nor disagree (3), Somewhat agree (4), Strongly disagree (5)

The NE CASC leadership demonstrates a commitment to meeting the needs of employees with disabilities.	34	Likert Scale	Strongly agree (1), Somewhat agree (2), Neither agree nor disagree (3), Somewhat agree (4), Strongly disagree (5)
The NE CASC leadership shows the importance of diversity through its actions.	50	Likert Scale	Strongly agree (1), Somewhat agree (2), Neither agree nor disagree (3), Somewhat agree (4), Strongly disagree (5)
The NE CASC leadership fosters a learning and workplace environment that allows employees to be themselves at work without fear.	50	Likert Scale	Strongly agree (1), Somewhat agree (2), Neither agree nor disagree (3), Somewhat agree (4), Strongly disagree (5)
The NE CASC leadership respects individuals and values their differences.	50	Likert Scale	Strongly agree (1), Somewhat agree (2), Neither agree nor disagree (3), Somewhat agree (4), Strongly disagree (5)
The NE CASC has policies and procedures that encourage diversity, equity, and inclusion.	50	Likert Scale	Strongly agree (1), Somewhat agree (2), Neither agree nor disagree (3), Somewhat agree (4), Strongly disagree (5)
If self-identified as a member of a marginalized group, What can the NE CASC do to support you as a member of a marginalized or underrepresented group?		Open Answer	NA
**How NE CASC supports the needs of NE CASC community members**			
My research and research goals are/were supported by the NE CASC and the NE CASC community	48	Likert Scale	Strongly agree (1), Somewhat agree (2), Neither agree nor disagree (3), Somewhat agree (4), Strongly disagree (5)
My professional and professional development goals are/were supported by the NE CASC Community.	49	Likert Scale	Strongly agree (1), Somewhat agree (2), Neither agree nor disagree (3), Somewhat agree (4), Strongly disagree (5)
My outreach and stakeholder engagement goals are/were supported by the NE CASC community.	48	Likert Scale	Strongly agree (1), Somewhat agree (2), Neither agree nor disagree (3), Somewhat agree (4), Strongly disagree (5)
I feel/felt comfortable and safe being myself within the NE CASC community	49	Likert Scale	Strongly agree (1), Somewhat agree (2), Neither agree nor disagree (3), Somewhat agree (4), Strongly disagree (5)

My identity and background are/were supported and accepted in the NE CASC community	49	Likert Scale	Strongly agree (1), Somewhat agree (2), Neither agree nor disagree (3), Somewhat agree (4), Strongly disagree (5)
I do/did not feel safe expressing my thoughts and feelings in the NE CASC community.	49	Likert Scale	Strongly agree (1), Somewhat agree (2), Neither agree nor disagree (3), Somewhat agree (4), Strongly disagree (5)
My experiences at NE CASC have led me to become more understanding of differences among my colleagues.	49	Likert Scale	Strongly agree (1), Somewhat agree (2), Neither agree nor disagree (3), Somewhat agree (4), Strongly disagree (5)
My religious identity is/was respected by the NE CASC community	47	Likert Scale	Strongly agree (1), Somewhat agree (2), Neither agree nor disagree (3), Somewhat agree (4), Strongly disagree (5)
My political identity is/was respected by the NE CASC community.	47	Likert Scale	Strongly agree (1), Somewhat agree (2), Neither agree nor disagree (3), Somewhat agree (4), Strongly disagree (5)
The NE CASC creates a space where I feel/felt like I can have a voice and my ideas are taken seriously.	49	Likert Scale	Strongly agree (1), Somewhat agree (2), Neither agree nor disagree (3), Somewhat agree (4), Strongly disagree (5)
I change(d) parts of my personality and personal presentation when I go to work to avoid conflict.	49	Likert Scale	Strongly agree (1), Somewhat agree (2), Neither agree nor disagree (3), Somewhat agree (4), Strongly disagree (5)
I feel/felt my voice has been heard and respected in development of NE CASC efforts for DEIJ.	47	Likert Scale	Strongly agree (1), Somewhat agree (2), Neither agree nor disagree (3), Somewhat agree (4), Strongly disagree (5)
**NE CASC Policies**			
Have you ever reported an incident of discrimination and/or bias during your time at NE CASC?	49	Multiple choice	Yes, No
If yes (above), Were you satisfied with the way that NE CASC leadership handled the reported incident?	4	Multiple choice	Yes, No
I believe that NE CASC leadership would take me seriously if I reported an incident of discrimination and/or bias.	48	Likert Scale	Strongly agree (1), Somewhat agree (2), Neither agree nor disagree (3), Somewhat agree (4), Strongly disagree (5)

S1 Table: NECASC climate survey questions, question types, and answer options.

**Experiences with supervisors**			
My supervisor(s) is committed to and supports diversity, equity, and inclusion.	49	Likert Scale	Strongly agree (1), Somewhat agree (2), Neither agree nor disagree (3), Somewhat agree (4), Strongly disagree (5)
My supervisor(s) handles matters related to diversity, equity, and inclusion satisfactorily.	49	Likert Scale	Strongly agree (1), Somewhat agree (2), Neither agree nor disagree (3), Somewhat agree (4), Strongly disagree (5)
**Experiences as supervisors**			
Do you supervise anyone? (including students)	49	Multiple choice	Yes, No
If yes (above), Do you feel supported and well-resourced to mentor students from diverse backgrounds?	40	Likert Scale	Strongly agree (1), Somewhat agree (2), Neither agree nor disagree (3), Somewhat agree (4), Strongly disagree (5)
**Recruitment**			
NE CASC takes active measures to seek a diverse candidate poll when recruiting new:			
Undergraduate Fellows	18	Likert Scale	Strongly agree (1), Somewhat agree (2), Neither agree nor disagree (3), Somewhat agree (4), Strongly disagree (5), I don't know (6)
Graduate Fellows	24	Likert Scale	Strongly agree (1), Somewhat agree (2), Neither agree nor disagree (3), Somewhat agree (4), Strongly disagree (5), I don't know (6)
Post Doctoral Fellows	22	Likert Scale	Strongly agree (1), Somewhat agree (2), Neither agree nor disagree (3), Somewhat agree (4), Strongly disagree (5), I don't know (6)
Affiliated Researchers	22	Likert Scale	Strongly agree (1), Somewhat agree (2), Neither agree nor disagree (3), Somewhat agree (4), Strongly disagree (5), I don't know (6)
Principal Investigators	23	Likert Scale	Strongly agree (1), Somewhat agree (2), Neither agree nor disagree (3), Somewhat agree (4), Strongly disagree (5), I don't know (6)
Stakeholders	25	Likert Scale	Strongly agree (1), Somewhat agree (2), Neither agree nor disagree (3), Somewhat agree (4), Strongly disagree (5), I don't know (6)

NE CASC follows a clear and transparent process when recruiting new:			
Undergraduate Fellows	17	Likert Scale	Strongly agree (1), Somewhat agree (2), Neither agree nor disagree (3), Somewhat agree (4), Strongly disagree (5), I don't know (6)
Graduate Fellows	21	Likert Scale	Strongly agree (1), Somewhat agree (2), Neither agree nor disagree (3), Somewhat agree (4), Strongly disagree (5), I don't know (6)
Post Doctoral Fellows	22	Likert Scale	Strongly agree (1), Somewhat agree (2), Neither agree nor disagree (3), Somewhat agree (4), Strongly disagree (5), I don't know (6)
Affiliated Researchers	19	Likert Scale	Strongly agree (1), Somewhat agree (2), Neither agree nor disagree (3), Somewhat agree (4), Strongly disagree (5), I don't know (6)
Principal Investigators	20	Likert Scale	Strongly agree (1), Somewhat agree (2), Neither agree nor disagree (3), Somewhat agree (4), Strongly disagree (5), I don't know (6)
Stakeholders	19	Likert Scale	Strongly agree (1), Somewhat agree (2), Neither agree nor disagree (3), Somewhat agree (4), Strongly disagree (5), I don't know (6)
The NE CASC processes for awarding research and fellowship funding are clear and transparent.	32	Likert Scale	Strongly agree (1), Somewhat agree (2), Neither agree nor disagree (3), Somewhat agree (4), Strongly disagree (5), I don't know (6)
**Retention**			
I have considered leaving science or academia over the last year	41	Likert Scale	Strongly agree (1), Somewhat agree (2), Neither agree nor disagree (3), Somewhat agree (4), Strongly disagree (5)
I have considered leaving academia or science research over the last year due to my identity.	41	Likert Scale	Strongly agree (1), Somewhat agree (2), Neither agree nor disagree (3), Somewhat agree (4), Strongly disagree (5)
**Current engagement with DEIJ initiatives, both within and outside of the NE CASC**			

What types of DEIJ efforts have you been involved in in the past year (2020 - 2021)?	49	Select all that apply	Department Committees, Outreach for K-12, Book groups, Planning seminars and inviting diverse speakers, De-colonizing curriculum materials, Mentoring students and creating safe spaces, Recruiting faculty from diverse backgrounds to engage in research, Recruiting and supporting students from diverse backgrounds, Self-education, Training, Challenging norms by existing as resistance (lived experience), Providing emotional labor by teaching others about DEIJ issues
How many training events and meetings have you attended over the past year (2020 - 2021) that included content about DEIJ issues?	42	Multiple choice	None, More than 1 but less than 3, More than 3 but less than 5, More than 5 but less than 10, More than 10
How many training events and discussions have you led or organized over the past year (2020 - 2021) that included content about DEIJ issues?	23	Multiple choice	None, More than 1 but less than 3, More than 3 but less than 5, More than 5 but less than 10, More than 11
What types of DEIJ training have you engaged with over the last year?	49	Select all that apply	Antibias Training, Anti-Racist Training, Inclusive Pedagogy, An ongoing book group, A talk or lecture, A workshop, Lived experience (BIPOC, LGBTQ*, other marginalized group)
On average, how often have you engaged in self-education (outside of training events) about DEIJ issues?	42	Multiple choice	None, At least once a year, At least once a semester, At least once a month, A few times a month, At least once a week, A few times a week, Daily
Over the last year, what actions have you taken in your research, outreach, mentoring, or teaching to address DEIJ issues and build a more inclusive academic environment?		Open answer	NA
Please list any relevant organizations or initiatives you are involved in that may benefit other members of NE CASC		Open answer	NA
**Future engagement with DEIJ initiatives within the NE CASC**			

What types of DEIJ efforts do you have planned for the coming year?	47	Select all that apply	Department Committees, Outreach for K-12, Book groups, Planning seminars and inviting diverse speakers, De-colonizing curriculum materials, Mentoring students and creating safe spaces, Recruiting faculty from diverse backgrounds to engage in research, Recruiting and supporting students from diverse backgrounds, Self-education, Training, Challenging norms by existing as resistance (lived experience), Providing emotional labor by teaching others about DEIJ issues
What is your biggest concern for the NE CASC as it moves forward with DEIJ initiatives?	NA	Open answer	
What are the challenges you are most concerned about when it comes to moving forward with your own DEIJ efforts?			
Having support from a supervisor	22	Sliding scale	0 (easy, not a problem) to 10 (biggest challenge)
Finding time to pursue activities	27	Sliding scale	0 (easy, not a problem) to 10 (biggest challenge)
Having enough funding/monetary support to do DEIJ work	28	Sliding scale	0 (easy, not a problem) to 10 (biggest challenge)
Having enough support from NE CASC leadership	20	Sliding scale	0 (easy, not a problem) to 10 (biggest challenge)
Sustainability of DEIJ programming over multiple years	32	Sliding scale	0 (easy, not a problem) to 10 (biggest challenge)
Having support from the university	22	Sliding scale	0 (easy, not a problem) to 10 (biggest challenge)
What are the challenges you are most concerned about when it comes to moving forward with your own research?			
Provide additional funding for graduate students	30	Sliding scale	0 (easy, not a problem) to 10 (biggest challenge)
Provide additional funding for undergraduate students	26	Sliding scale	0 (easy, not a problem) to 10 (biggest challenge)
Improve office space	12	Sliding scale	0 (easy, not a problem) to 10 (biggest challenge)

Provide funding to support childcare needs	18	Sliding scale	0 (easy, not a problem) to 10 (biggest challenge)
Make efforts to improve Paid Time Off options	14	Sliding scale	0 (easy, not a problem) to 10 (biggest challenge)
Clarify responsibilities of different positions	22	Sliding scale	0 (easy, not a problem) to 10 (biggest challenge)
What can the NE CASC do to better support you and your DEIJ efforts?			
Provide funding to support existing efforts	27	Sliding scale	0 (not important at all) to 10 (most important)
Provide additional funding for graduate students	27	Sliding scale	0 (not important at all) to 10 (most important)
Provide additional funding for undergraduate students	23	Sliding scale	0 (not important at all) to 10 (most important)
Provide funding to support professional development opportunities	23	Sliding scale	0 (not important at all) to 10 (most important)
Make efforts to improve Paid Time Off options	15	Sliding scale	0 (not important at all) to 10 (most important)
Include DEIJ efforts in job responsibilities	24	Sliding scale	0 (not important at all) to 10 (most important)
Create clearer DEIJ policies	25	Sliding scale	0 (not important at all) to 10 (most important)
**Experiences working remotely during the COVID-19 pandemic and feelings about returning to the workplace**			
I have felt well supported by the NE CASC during my time working remotely during the COVID-19 pandemic.	42	Likert Scale	Strongly agree (1), Somewhat agree (2), Neither agree nor disagree (3), Somewhat agree (4), Strongly disagree (5)
I have had adequate access to the technology and resources I need to work remotely during the COVID-19 pandemic.	42	Likert Scale	Strongly agree (1), Somewhat agree (2), Neither agree nor disagree (3), Somewhat agree (4), Strongly disagree (5)
I feel more isolated working from home than in person.	42	Likert Scale	Strongly agree (1), Somewhat agree (2), Neither agree nor disagree (3), Somewhat agree (4), Strongly disagree (5)

I miss my colleagues and I miss working on a group environment.	42	Likert Scale	Strongly agree (1), Somewhat agree (2), Neither agree nor disagree (3), Somewhat agree (4), Strongly disagree (5)
I miss attending department events and informal get togethers in person (happy hours, seminars, networking sessions).	42	Likert Scale	Strongly agree (1), Somewhat agree (2), Neither agree nor disagree (3), Somewhat agree (4), Strongly disagree (5)
I feel more comfortable and productive working remotely.	42	Likert Scale	Strongly agree (1), Somewhat agree (2), Neither agree nor disagree (3), Somewhat agree (4), Strongly disagree (5)
Working remotely has allowed me to complete my work while also living in an environment that feels safe and comfortable for me.	42	Likert Scale	Strongly agree (1), Somewhat agree (2), Neither agree nor disagree (3), Somewhat agree (4), Strongly disagree (5)
Working remotely has allowed me to live in a location where I feel more comfortable in my identity.	42	Likert Scale	Strongly agree (1), Somewhat agree (2), Neither agree nor disagree (3), Somewhat agree (4), Strongly disagree (5)
Working remotely has allowed me to avoid microaggressions and other conflicts at work.	42	Likert Scale	Strongly agree (1), Somewhat agree (2), Neither agree nor disagree (3), Somewhat agree (4), Strongly disagree (5)
Zoom calls about DEIJ issues have been very taxing due to the level of effort requested of me.	41	Likert Scale	Strongly agree (1), Somewhat agree (2), Neither agree nor disagree (3), Somewhat agree (4), Strongly disagree (5)
Over the last year, I have had to do a lot of emotional labor around DEIJ issues.	42	Likert Scale	Strongly agree (1), Somewhat agree (2), Neither agree nor disagree (3), Somewhat agree (4), Strongly disagree (5)
Over the last year, I have been asked to support and participate in DEIJ events and efforts because of my identity.	42	Likert Scale	Strongly agree (1), Somewhat agree (2), Neither agree nor disagree (3), Somewhat agree (4), Strongly disagree (5)
Remote work has been difficult for me because of my caretaking responsibilities.	42	Likert Scale	Strongly agree (1), Somewhat agree (2), Neither agree nor disagree (3), Somewhat agree (4), Strongly disagree (5)
I am nervous about returning to work and in person events because of COVID	42	Likert Scale	Strongly agree (1), Somewhat agree (2), Neither agree nor disagree (3), Somewhat agree (4), Strongly disagree (5)
I'm nervous about returning to in person events and work due to my identity.	42	Likert Scale	Strongly agree (1), Somewhat agree (2), Neither agree nor disagree (3), Somewhat agree (4), Strongly disagree (5)

I am nervous about returning to work in person because I do not have a safe or comfortable office space.	42	Likert Scale	Strongly agree (1), Somewhat agree (2), Neither agree nor disagree (3), Somewhat agree (4), Strongly disagree (5)
I hope to continue working from home at least a few days a week even after people start return to work in person.	42	Likert Scale	Strongly agree (1), Somewhat agree (2), Neither agree nor disagree (3), Somewhat agree (4), Strongly disagree (5)
