# Supplementary material for: Centering voices of scientists from marginalized backgrounds to understand experiences in climate adaptation science and inform action
Source: PLoS One. 2025 Feb 21;20(2):e0318438. doi: 10.1371/journal.pone.0318438 (PMC11844896; doi:10.1371/journal.pone.0318438)
Supplement: S1 Appendix — Anonymized data from our study figures and analyses for each reported question and figure. (PDF) [file pone.0318438.s002.pdf]

Figure 1) Demographic Data Information (Anonymized)

Category	Responses (n)
Disability	
I do not have a disability	33
Yes, I have a disability	5
Other	1
Seen unanswered	3
Race	
White, Caucasian	35
Mixed Race	1
Hispanic, Latino	2
Black, African American	1
Asian or Asian American	0
S. Asian or S. Asian American	1
Indigenous American, Native American, AIAN (American Indian/Alaska Native)	1
Native Hawaiian, Pacific Islander	0
North African, Middle Eastern	0
Sexuality	
Heterosexual or straight	33
Gay	0
Lesbian	1
Bisexual	0
Queer	3
Pansexual	0
Asexual/Aromantic	1
Prefer not to disclose	1
Seen unanswered	3
Marginalized	
No	22
Yes	10
Seen unanswered	1
Gender	
Cisgender woman	22
Cisgender man	16
Transgender woman	0
Transgender man	0
Non-binary/Gender non-coforming	0
Prefer not to respond	1
Seen unanswered	3
Age	
younger (<45)	27
older (>45)	12

seen unanswered	3
Career stage	
fellow	21
non-fellow	19
seen unanswered	4

Figure 3) Feelings of Acceptance within the NE CASC community

**Figure 3A-3E**	My identity and background were supported and accepted in the NE CASC
**Figure 3F-3J**	NE CASC members with different backgrounds interact well with each other.
**Figure 3K-3O**	I felt comfortable and safe being myself within the NE CASC community.

Figure 3A-3E

3A) Total

My identity and background were supported and accepted in the NE CASC community.

Response	Count
Unfinished	14
Valid skip	0
I don't know.	0
Seen unanswered	2
Strongly disagree	2
Somewhat disagree	3
Neither agree nor disagree	2
Somewhat agree	8
Strongly agree	34

3B) Marginalized vs. Not-Marginalized

	Marginalizec	No	Other
Unfinished	0	0	14
Valid skip	0	0	0
I don't know.	0	0	0
Seen unanswered	0	0	2
Strongly disagree	2	0	0
Somewhat disagree	1	2	0
Neither agree nor disagree	1	0	1
Somewhat agree	4	3	1
Strongly agree	2	17	15

3C) 25-44 years vs. 45-74 years

	Older	Younger	Other
Unfinished	0	0	14
Valid skip	0	0	0
I don't know.	0	0	0
Seen unanswered	0	0	2
Strongly disagree	0	1	1
Somewhat disagree	0	2	1
Neither agree nor disagree	1	1	0
Somewhat agree	3	4	1
Strongly agree	8	19	7

3D) Fellow vs. Not-Fellow

	Fellow	not-fellow	Other
Unfinished	0	0	14
Valid skip	0	0	0
I don't know.	0	0	0
Seen unanswered	0	0	2
Strongly disagree	1	0	1
Somewhat disagree	2	0	1
Neither agree nor disagree	1	0	1
Somewhat agree	3	4	1
Strongly agree	13	14	7

3E) Cisgender Man vs. Cisgender Woman

	Cisgender Man	Cisgender Woman	Transgender Man	Transgender Woman	Non-binary/ Gender non-conforming	Other/ prefer not to respond
Unfinished	0	0	0	0	0	14
Valid skip	0	0	0	0	0	0
I don't know.	0	0	0	0	0	0
Seen unanswered	0	0	0	0	0	2
Strongly disagree	1	0	0	0	0	1

Somewhat disagree	0	2	0	0	0	1
Neither agree nor disagree	0	2	0	0	0	0
Somewhat agree	1	6	0	0	0	1
Strongly agree	14	12	0	0	0	8

Figure 3F-3J

3F) Total

NE CASC members with different backgrounds interact well with each other. (1)

Response	Count
Unfinished	9
Valid skip	0
I don't know.	0
Seen unanswered	4
Strongly disagree	1
Somewhat disagree	5
Neither agree nor disagree	10
Somewhat agree	16
Strongly agree	20

3G) Marginalized vs. Not-Marginalized

	Marginalizec	No	Other
Unfinished	0	0	9
Valid skip	0	0	0
I don't know.	0	0	0
Seen unanswered	0	0	4
Strongly disagree	0	0	1
Somewhat disagree	2	2	1
Neither agree nor disagree	5	2	3
Somewhat agree	0	8	8
Strongly agree	3	10	7

3H) 25-44 years vs. 45-74 years

	Older	Younger	Other
Unfinished	0	0	9

Valid skip	0	0	0
I don't know.	0	0	0
Seen unanswered	0	0	4
Strongly disagree	0	0	1
Somewhat disagree	2	3	0
Neither agree nor disagree	2	4	4
Somewhat agree	3	11	2
Strongly agree	5	9	6

3I) Fellow vs. Not-Fellow

	Fellow	not-fellow	Other
Unfinished	0	0	9
Valid skip	0	0	0
I don't know.	0	0	0
Seen unanswered	0	0	4
Strongly disagree	0	0	1
Somewhat disagree	3	1	1
Neither agree nor disagree	3	3	4
Somewhat agree	10	4	2
Strongly agree	4	10	6

3J) Cisgender Man vs. Cisgender Woman

	Cisgender Man	Cisgender Woman	Transgender Man	Transgender Woman	Non-binary/ Gender non-conforming	Other/ prefer not to respond
Unfinished	0	0	0	0	0	9
Valid skip	0	0	0	0	0	0
I don't know.	0	0	0	0	0	0
Seen unanswered	0	0	0	0	0	4
Strongly disagree	0	0	0	0	0	1
Somewhat disagree	0	5	0	0	0	0
Neither agree nor disagree	3	3	0	0	0	4
Somewhat agree	4	9	0	0	0	3

Strongly agree	9	5	0	0	0	6

Figure 3K-3O

3K) Total

I felt comfortable and safe being myself within the NE CASC community.

Response	Count
Unfinished	14
Valid skip	0
I don't know.	0
Seen unanswered	2
Strongly disagree	0
Somewhat disagree	7
Neither agree nor disagree	1
Somewhat agree	11
Strongly agree	30

3L) Marginalized vs. Not-Marginalized

	Marginalizec	No	Other
Unfinished	0	0	14
Valid skip	0	0	0
I don't know.	0	0	0
Seen unanswered	0	0	2
Strongly disagree	0	0	0
Somewhat disagree	5	2	0
Neither agree nor disagree	0	0	1
Somewhat agree	4	5	2
Strongly agree	1	15	14

3M) 25-44 years vs. 45-74 years

	Older	Younger	Other
Unfinished	0	0	14
Valid skip	0	0	0
I don't know.	0	0	0

Seen unanswered	0	0	2
Strongly disagree	0	0	0
Somewhat disagree	1	4	2
Neither agree nor disagree	0	1	0
Somewhat agree	5	6	0
Strongly agree	6	16	8

3N) Fellow vs. Not-Fellow

	Fellow	not-fellow	Other
Unfinished	0	0	14
Valid skip	0	0	0
I don't know.	0	0	0
Seen unanswered	0	0	2
Strongly disagree	0	0	0
Somewhat disagree	4	0	3
Neither agree nor disagree	1	0	0
Somewhat agree	6	5	0
Strongly agree	9	13	8

3O) Cisgender Man vs. Cisgender Woman

	Cisgender Man	Cisgender Woman	Transgender Man	Transgender Woman	Non-binary/ Gender non-conforming	Other/ prefer not to respond
Unfinished	0	0	0	0	0	14
Valid skip	0	0	0	0	0	0
I don't know.	0	0	0	0	0	0
Seen unanswered	0	0	0	0	0	2
Strongly disagree	0	0	0	0	0	0
Somewhat disagree	1	4	0	0	0	2
Neither agree nor disagree	0	1	0	0	0	0
Somewhat agree	4	7	0	0	0	0
Strongly agree	11	10	0	0	0	9

Figure 4) Responses to Goals Support

**4A-4E**	My research and research goals were supported by the NE CASC and the NE CASC community.
**4F-4J**	My professional and professional development goals were supported by the NE CASC
**4K-4O**	My outreach and stakeholder engagement goals were supported by the NE CASC community.

Figure 4A-4E

4A) Total

My research and research goals were supported by the NE CASC and the NE CASC community.

Response	Count
Unfinished	14
Valid skip	0
I don't know.	0
Seen unanswered	3
Strongly disagree	0
Somewhat disagree	3
Neither agree nor disagree	5
Somewhat agree	15
Strongly agree	25

4B) Marginalized vs. Not-Marginalized

	Marginalized	No	Other
Unfinished	0	0	14
Valid skip	0	0	0
I don't know.	0	0	0
Seen unanswered	0	0	3
Strongly disagree	0	0	0
Somewhat disagree	3	0	0
Neither agree nor disagree	2	2	1
Somewhat agree	2	6	7
Strongly agree	3	14	8

4C) 25-44 years vs. 45-74 years

	Older	Younger	Other
Unfinished	0	0	14
Valid skip	0	0	0
I don't know.	0	0	0
Seen unanswered	1	0	2
Strongly disagree	0	0	0
Somewhat disagree	2	1	0
Neither agree nor disagree	0	2	3
Somewhat agree	2	10	3
Strongly agree	7	14	4

4D) Fellow vs. Not-Fellow

	Fellow	not-fellow	Other
Unfinished	0	0	14
Valid skip	0	0	0
I don't know.	0	0	0
Seen unanswered	0	1	2
Strongly disagree	0	0	0
Somewhat disagree	1	1	1
Neither agree nor disagree	2	0	3
Somewhat agree	8	4	3
Strongly agree	9	12	4

4E) Cisgender Man vs. Cisgender Woman

	Cisgender Man	Cisgender Woman	Other
Unfinished	0	0	14
Valid skip	0	0	0
I don't know.	0	0	0
Seen unanswered	0	1	2
Strongly disagree	0	0	0
Somewhat disagree	0	3	0
Neither agree nor disagree	0	2	3
Somewhat agree	4	8	3
Strongly agree	12	8	5

Figure 4F-4J

4F) Total

My professional and professional development goals were supported by the NE CASC community.

Response	Count
Unfinished	14
Valid skip	0
I don't know.	0
Seen unanswered	2
Strongly disagree	0
Somewhat disagree	4
Neither agree nor disagree	4
Somewhat agree	16
Strongly agree	25

4G) Marginalized vs. Not-Marginalized

	Marginalized	No	Other
Unfinished	0	0	14
Valid skip	0	0	0
I don't know.	0	0	0
Seen unanswered	0	0	2

Strongly disagree	0	0	0
Somewhat disagree	3	1	0
Neither agree nor disagree	1	2	1
Somewhat agree	3	7	6
Strongly agree	3	12	10

4H) 25-44 years vs. 45-74 years

	Older	Younger	Other
Unfinished	0	0	14
Valid skip	0	0	0
I don't know.	0	0	0
Seen unanswered	0	0	2
Strongly disagree	0	0	0
Somewhat disagree	2	2	0
Neither agree nor disagree	1	2	1
Somewhat agree	4	9	3
Strongly agree	5	14	6

4I) Fellow vs. Not-Fellow

	Fellow	not-fellow	Other
Unfinished	0	0	14
Valid skip	0	0	0
I don't know.	0	0	0
Seen unanswered	0	0	2
Strongly disagree	0	0	0
Somewhat disagree	1	2	1
Neither agree nor disagree	2	1	1
Somewhat agree	9	4	3
Strongly agree	8	11	6

4J) Cisgender Man vs. Cisgender Woman

	Cisgender Man	Cisgender Woman	Other
Unfinished	0	0	14
Valid skip	0	0	0
I don't know.	0	0	0
Seen unanswered	0	0	2
Strongly disagree	0	0	0
Somewhat disagree	0	4	0
Neither agree nor disagree	0	3	1
Somewhat agree	4	8	4
Strongly agree	12	7	6

Figure 4K-4O

4K) Total

My outreach and stakeholder engagement goals were supported by the NE CASC community.

Response	Count
Unfinished	14
Valid skip	0
I don't know.	0
Seen unanswered	3
Strongly disagree	0
Somewhat disagree	1
Neither agree nor disagree	13
Somewhat agree	12
Strongly agree	22

4L) Marginalized vs. Not-Marginalized

	Marginalized	No	Other
Unfinished	0	0	14
Valid skip	0	0	0
I don't know.	0	0	0
Seen unanswered	0	0	3
Strongly disagree	0	0	0
Somewhat disagree	1	0	0
Neither agree nor disagree	3	3	7
Somewhat agree	3	4	5
Strongly agree	3	15	4

4M) 25-44 years vs. 45-74 years

	Older	Younger	Other
Unfinished	0	0	14
Valid skip	0	0	0
I don't know.	0	0	0
Seen unanswered	0	1	2
Strongly disagree	0	0	0
Somewhat disagree	1	0	0
Neither agree nor disagree	2	8	3
Somewhat agree	3	6	3
Strongly agree	6	12	4

4N) Fellow vs. Not-Fellow

	Fellow	not-fellow	Other
Unfinished	0	0	14
Valid skip	0	0	0
I don't know.	0	0	0
Seen unanswered	1	0	2
Strongly disagree	0	0	0
Somewhat disagree	0	0	1
Neither agree nor disagree	7	3	3
Somewhat agree	5	4	3
Strongly agree	7	11	4

4O) Cisgender Man vs. Cisgender Woman

	Cisgender Man	Cisgender Woman	Other
Unfinished	0	0	14
Valid skip	0	0	0
I don't know.	0	0	0
Seen unanswered	1	0	2
Strongly disagree	0	0	0
Somewhat disagree	0	1	0
Neither agree nor disagree	4	6	3
Somewhat agree	2	7	3
Strongly agree	9	8	5

Figure 4) Responses to Goals Support

**4A-4E**	My research and research goals were supported by the NE CASC and the NE CASC community.
**4F-4J**	My professional and professional development goals were supported by the NE CASC community.
**4K-4O**	My outreach and stakeholder engagement goals were supported by the NE CASC community.

Figure 4A-4E

4A) Total

My research and research goals were supported by the NE CASC and the NE CASC community.

Response	Count
Unfinished	14
Valid skip	0
I don't know.	0
Seen unanswered	3
Strongly disagree	0
Somewhat disagree	3
Neither agree nor disagree	5
Somewhat agree	15
Strongly agree	25

4B) Marginalized vs. Not-Marginalized

	Marginalized	No	Other
Unfinished	0	0	14
Valid skip	0	0	0
I don't know.	0	0	0
Seen unanswered	0	0	3

Strongly disagree	0	0	0
Somewhat disagree	3	0	0
Neither agree nor disagree	2	2	1
Somewhat agree	2	6	7
Strongly agree	3	14	8

4C) 25-44 years vs. 45-74 years

	Older	Younger	Other
Unfinished	0	0	14
Valid skip	0	0	0
I don't know.	0	0	0
Seen unanswered	1	0	2
Strongly disagree	0	0	0
Somewhat disagree	2	1	0
Neither agree nor disagree	0	2	3
Somewhat agree	2	10	3
Strongly agree	7	14	4

4D) Fellow vs. Not-Fellow

	Fellow	not-fellow	Other
Unfinished	0	0	14
Valid skip	0	0	0
I don't know.	0	0	0
Seen unanswered	0	1	2
Strongly disagree	0	0	0
Somewhat disagree	1	1	1
Neither agree nor disagree	2	0	3
Somewhat agree	8	4	3
Strongly agree	9	12	4

4E) Cisgender Man vs. Cisgender Woman

	Cisgender Man	Cisgender Woman	Transgender Man	Transgender Woman	Non-binary/ Gender non-conforming	Other/ prefer not to respond
Unfinished	0	0	0	0	0	14
Valid skip	0	0	0	0	0	0
I don't know.	0	0	0	0	0	0
Seen unanswered	0	1	0	0	0	2
Strongly disagree	0	0	0	0	0	0
Somewhat disagree	0	3	0	0	0	0
Neither agree nor disagree	0	2	0	0	0	3
Somewhat agree	4	8	0	0	0	3
Strongly agree	12	8	0	0	0	5

Figure 4F-4J

4F) Total

My professional and professional development goals were supported by the NE CASC community.

Response	Count
Unfinished	14
Valid skip	0
I don't know.	0
Seen unanswered	2
Strongly disagree	0
Somewhat disagree	4
Neither agree nor disagree	4
Somewhat agree	16
Strongly agree	25

4G) Marginalized vs. Not-Marginalized

	Marginalized	No	Other
Unfinished	0	0	14
Valid skip	0	0	0
I don't know.	0	0	0

Seen unanswered	0	0	2
Strongly disagree	0	0	0
Somewhat disagree	3	1	0
Neither agree nor disagree	1	2	1
Somewhat agree	3	7	6
Strongly agree	3	12	10

4H) 25-44 years vs. 45-74 years

	Older	Younger	Other
Unfinished	0	0	14
Valid skip	0	0	0
I don't know.	0	0	0
Seen unanswered	0	0	2
Strongly disagree	0	0	0
Somewhat disagree	2	2	0
Neither agree nor disagree	1	2	1
Somewhat agree	4	9	3
Strongly agree	5	14	6

4I) Fellow vs. Not-Fellow

	Fellow	not-fellow	Other
Unfinished	0	0	14
Valid skip	0	0	0
I don't know.	0	0	0
Seen unanswered	0	0	2
Strongly disagree	0	0	0
Somewhat disagree	1	2	1
Neither agree nor disagree	2	1	1
Somewhat agree	9	4	3
Strongly agree	8	11	6

4J) Cisgender Man vs. Cisgender Woman

	Cisgender Man	Cisgender Woman	Transgender Man	Transgender Woman	Non-binary/ Gender non-conforming	Other/ prefer not to respond
Unfinished	0	0	0	0	0	14
Valid skip	0	0	0	0	0	0
I don't know.	0	0	0	0	0	0
Seen unanswered	0	0	0	0	0	2
Strongly disagree	0	0	0	0	0	0
Somewhat disagree	0	4	0	0	0	0
Neither agree nor disagree	0	3	0	0	0	1
Somewhat agree	4	8	0	0	0	4
Strongly agree	12	7	0	0	0	6

Figure 4K-4O

4K) Total

My outreach and stakeholder engagement goals were supported by the NE CASC community.

Response	Count
Unfinished	14
Valid skip	0
I don't know.	0
Seen unanswered	3
Strongly disagree	0
Somewhat disagree	1
Neither agree nor disagree	13
Somewhat agree	12
Strongly agree	22

4L) Marginalized vs. Not-Marginalized

	Marginalized	No	Other
Unfinished	0	0	14
Valid skip	0	0	0
I don't know.	0	0	0

Seen unanswered	0	0	3
Strongly disagree	0	0	0
Somewhat disagree	1	0	0
Neither agree nor disagree	3	3	7
Somewhat agree	3	4	5
Strongly agree	3	15	4

4M) 25-44 years vs. 45-74 years

	Older	Younger	Other
Unfinished	0	0	14
Valid skip	0	0	0
I don't know.	0	0	0
Seen unanswered	0	1	2
Strongly disagree	0	0	0
Somewhat disagree	1	0	0
Neither agree nor disagree	2	8	3
Somewhat agree	3	6	3
Strongly agree	6	12	4

4N) Fellow vs. Not-Fellow

	Fellow	not-fellow	Other
Unfinished	0	0	14
Valid skip	0	0	0
I don't know.	0	0	0
Seen unanswered	1	0	2
Strongly disagree	0	0	0
Somewhat disagree	0	0	1
Neither agree nor disagree	7	3	3
Somewhat agree	5	4	3
Strongly agree	7	11	4

4O) Cisgender Man vs. Cisgender Woman

	Cisgender Man	Cisgender Woman	Transgender Man	Transgender Woman	Non-binary/ Gender non-conforming	Other/ prefer not to respond
Unfinished	0	0	0	0	0	14
Valid skip	0	0	0	0	0	0
I don't know.	0	0	0	0	0	0
Seen unanswered	1	0	0	0	0	2
Strongly disagree	0	0	0	0	0	0
Somewhat disagree	0	1	0	0	0	0
Neither agree nor disagree	4	6	0	0	0	3
Somewhat agree	2	7	0	0	0	3
Strongly agree	9	8	0	0	0	5

Figure 5) Leadership and DEI

**Figure 5A-5E**	My supervisor(s) is committed to and supports diversity, equity, and inclusion.
**Figure 5F-5J**	My supervisor(s) handles matters related to diversity, equity, and inclusion satisfactorily.
**Figure 5K-5O**	NE CASC leadership shows the importance of DEIJ though its actions.

Figure 5A-5E

Total

My supervisor(s) is committed to and supports diversity, equity, and inclusion.

Response	Count
Unfinished	14
Valid skip	0
I don't know.	0
Seen unanswere	2
Strongly disagree	1
Somewhat disagı	3
Neither agree no	3
Somewhat agree	14
Strongly agree	28

Marginalized vs. Not-Marginalized

	Marginalized	No	Other
Unfinished	0	0	14
Valid skip	0	0	0
I don't know.	0	0	0
Seen unanswerec	0	0	2
Strongly disagree	0	1	0
Somewhat disagr	0	1	2

Neither agree nor	1	0	2
Somewhat agree	4	5	5
Strongly agree	5	15	8

25-44 years vs. 45-74 years

	Older	Younger	Other
Unfinished	0	0	14
Valid skip	0	0	0
I don't know.	0	0	0
Seen unanswered	0	0	2
Strongly disagree	0	1	0
Somewhat disagr	0	2	1
Neither agree nor	1	1	1
Somewhat agree	2	11	1
Strongly agree	9	12	7

Fellow vs. Not-Fellow

	Fellow	not-fellow	Other
Unfinished	0	0	14
Valid skip	0	0	0
I don't know.	0	0	0
Seen unanswered	0	0	2
Strongly disagree	1	0	0
Somewhat disagr	2	0	1
Neither agree nor	1	1	1
Somewhat agree	8	5	1
Strongly agree	8	12	8

Gender

	Cisgender Man	Cisgender Woman	Transgender Man	Transgender Woman	Non-binary/ Gender non-conforming	Other/ prefer not to respond
Unfinished	0	0	0	0	0	14

Valid skip	0	0	0	0	0	0
I don't know.	0	0	0	0	0	0
Seen unanswered	0	0	0	0	0	2
Strongly disagree	0	1	0	0	0	0
Somewhat disagr	2	0	0	0	0	1
Neither agree nor	0	1	0	0	0	2
Somewhat agree	6	7	0	0	0	1
Strongly agree	8	13	0	0	0	7

Figure 5F-5J

Total

My supervisor(s) handles matters related to diversity, equity, and inclusion satisfactorily.

Response	Count
Unfinished	14
Valid skip	0
I don't know.	0
Seen unanswere	2
Strongly disagree	2
Somewhat disagr	3
Neither agree no	11
Somewhat agree	10
Strongly agree	23

Marginalized vs. Not-Marginalized

	Marginalized	No	Other
Unfinished	0	0	14
Valid skip	0	0	0
I don't know.	0	0	0
Seen unanswered	0	0	2
Strongly disagree	0	2	0
Somewhat disagr	2	0	1
Neither agree nor	3	3	5
Somewhat agree	2	5	3

Strongly agree	3	12	8

25-44 years vs. 45-74 years			
	Older	Younger	Other
Unfinished	0	0	14
Valid skip	0	0	0
I don't know.	0	0	0
Seen unanswerec	0	0	2
Strongly disagree	0	2	0
Somewhat disagr	0	2	1
Neither agree nor	3	6	2
Somewhat agree	2	7	1
Strongly agree	7	10	6

Fellow vs. Not-Fellow			
	Fellow	not-fellow	Other
Unfinished	0	0	14
Valid skip	0	0	0
I don't know.	0	0	0
Seen unanswerec	0	0	2
Strongly disagree	1	1	0
Somewhat disagr	2	0	1
Neither agree nor	4	5	2
Somewhat agree	6	2	2
Strongly agree	7	10	6

Cisgender Man vs. Cisgender Woman						
	Cisgender Man	Cisgender Woman	Transgender Man	Transgender Woman	Non-binary/ Gender non-conforming	Other/ prefer not to respond
Unfinished	0	0	0	0	0	14
Valid skip	0	0	0	0	0	0
I don't know.	0	0	0	0	0	0

Seen unanswerec	0	0	0	0	0	2
Strongly disagree	1	1	0	0	0	0
Somewhat disagr	2	0	0	0	0	1
Neither agree nor	2	6	0	0	0	3
Somewhat agree	3	6	0	0	0	1
Strongly agree	8	9	0	0	0	6

Figure 5K-5O

Total

NE CASC leadership shows the importance of DEIJ though its actions.

Response	Count
Unfinished	12
Valid skip	0
I don't know.	0
Seen unanswere	3
Strongly disagree	0
Somewhat disagı	8
Neither agree no	11
Somewhat agree	12
Strongly agree	19

Marginalized vs. Not-Marginalized

	Marginalized	No	Other
Unfinished	0	0	12
Valid skip	0	0	0
I don't know.	0	0	0
Seen unanswerec	0	0	3
Strongly disagree	0	0	0
Somewhat disagr	4	2	2
Neither agree nor	2	2	7
Somewhat agree	2	5	5
Strongly agree	2	13	4

25-44 years vs. 45-74 years			
	Older	Younger	Other
Unfinished	0	0	12
Valid skip	0	0	0
I don't know.	0	0	0
Seen unanswerec	0	0	3
Strongly disagree	0	0	0
Somewhat disagr	1	5	2
Neither agree nor	4	4	3
Somewhat agree	3	7	2
Strongly agree	4	11	4

Fellow vs. Not-Fellow			
	Fellow	not-fellow	Other
Unfinished	0	0	12
Valid skip	0	0	0
I don't know.	0	0	0
Seen unanswerec	0	0	3
Strongly disagree	0	0	0
Somewhat disagr	5	1	2
Neither agree nor	5	2	4
Somewhat agree	5	5	2
Strongly agree	5	10	4

Cisgender Man vs. Cisgender Woman						
	Cisgender Man	Cisgender Woman	Transgender Man	Transgender Woman	Non-binary/ Gender non-conforming	Other/ prefer not to respond
Unfinished	0	0	0	0	0	12
Valid skip	0	0	0	0	0	0
I don't know.	0	0	0	0	0	0
Seen unanswerec	0	0	0	0	0	3
Strongly disagree	0	0	0	0	0	0
Somewhat disagr	1	5	0	0	0	2

Neither agree nor	3	4	0	0	0	4
Somewhat agree	7	3	0	0	0	2
Strongly agree	5	10	0	0	0	4

Figure 6) Considering leaving science or academia

**Figure 6A-6E**	I have considered leaving science or academia over the last year
**Figure 6F-6J**	I have considered leaving academia or science research over the last year due to my identity.

Figure 6A-6E

Total

I have considered leaving science or academia over the last year

Response	Count
Unfinished	21
Valid skip	0
I don't know.	0
Seen unanswered	3
Strongly disagree	21
Somewhat disagree	5
Neither agree nor disagree	5
Somewhat agree	2
Strongly agree	8

Marginalized vs. Not-Marginalized

	Marginalize	No	Other
Unfinished	1	1	19
Valid skip	0	0	0
I don't know.	0	0	0
Seen unanswered	0	0	3
Strongly disagree	2	13	6
Somewhat disagree	1	3	1
Neither agree nor disagree	1	0	4
Somewhat agree	1	1	0
Strongly agree	4	4	0

25-44 years vs. 45-74 years

	Older	Younger	Other
Unfinished	0	0	21
Valid skip	0	0	0
I don't know.	0	0	0
Seen unanswered	1	0	2
Strongly disagree	5	14	2
Somewhat disagree	1	4	0
Neither agree nor disagree	1	4	0
Somewhat agree	1	1	0
Strongly agree	3	4	1

Fellow vs. Not-Fellow

	Fellow	not-fellow	Other
Unfinished	0	0	21
Valid skip	0	0	0
I don't know.	0	0	0
Seen unanswered	0	1	2
Strongly disagree	7	11	3
Somewhat disagree	3	2	0
Neither agree nor disagree	4	1	0
Somewhat agree	1	1	0
Strongly agree	5	2	1

Gender

	Cisgender Man	Cisgender Woman	Transgender Man	Transgender Woman	Non-binary/ Gender non-conforming	Other/ prefer not to respond
Unfinished	0	0	0	0	0	21
Valid skip	0	0	0	0	0	0
I don't know.	0	0	0	0	0	0

Seen unanswered	0	1	0	0	0	2
Strongly disagree	9	9	0	0	0	3
Somewhat disagree	1	4	0	0	0	0
Neither agree nor disagree	3	2	0	0	0	0
Somewhat agree	0	2	0	0	0	0
Strongly agree	3	4	0	0	0	1

Figure 6F-6J

Total

I have considered leaving academia or science research over the last year due to my identity.

Var1	Freq
Unfinished	21
Valid skip	0
I don't know.	0
Seen unanswered	3
Strongly disagree	26
Somewhat disagree	4
Neither agree nor disagree	5
Somewhat agree	4
Strongly agree	2

Marginalized vs. Not-Marginalized

	Marginalize	No	Other
Unfinished	1	1	19
Valid skip	0	0	0
I don't know.	0	0	0
Seen unanswered	0	0	3
Strongly disagree	3	17	6
Somewhat disagree	2	1	1
Neither agree nor disagree	0	1	4

Somewhat agree	2	2	0
Strongly agree	2	0	0

25-44 years vs. 45-74 years			
	Older	Younger	Other
Unfinished	0	0	21
Valid skip	0	0	0
I don't know.	0	0	0
Seen unanswered	1	0	2
Strongly disagree	9	15	2
Somewhat disagree	1	3	0
Neither agree nor disagree	0	5	0
Somewhat agree	1	3	0
Strongly agree	0	1	1

Fellow vs. Not-Fellow			
	Fellow	not-fellow	Other
Unfinished	0	0	21
Valid skip	0	0	0
I don't know.	0	0	0
Seen unanswered	0	1	2
Strongly disagree	7	16	3
Somewhat disagree	3	1	0
Neither agree nor disagree	5	0	0
Somewhat agree	4	0	0
Strongly agree	1	0	1

Gender						
	Cisgender Man	Cisgender Woman	Transgender Man	Transgender Woman	Non-binary/ Gender non-conforming	Other/ prefer not to respond
Unfinished	0	0	0	0	0	21

Valid skip	0	0	0	0	0	0
I don't know.	0	0	0	0	0	0
Seen unanswered	0	1	0	0	0	2
Strongly disagree	12	11	0	0	0	3
Somewhat disagree	1	3	0	0	0	0
Neither agree nor disagree	1	4	0	0	0	0
Somewhat agree	1	3	0	0	0	0
Strongly agree	1	0	0	0	0	1

Figure 7: Challenges in DEIJ efforts

What are the challenges you are most concerned about when it comes to moving forward with your own DEIJ efforts? Please move the slider for each challenge on a scale of 0 (easy, not

Finding time to pursue activities ()	
Total	
Challenges - Finding time to pursue activities	
Response	Count
Seen unanswered	6
Unfinished	20
Valid skip	14
0	1
1	1
2	0
3	1
4	2
5	3
6	2
7	2
8	3
9	7
10	5

Having enough funding/monetary support to do DEIJ work ()	
Total	
Challenges - Having enough funding/monetary support to do DEIJ work	
Response	Count
Seen unanswered	5
Unfinished	20
Valid skip	14
0	1
1	1
2	3
3	3
4	1
5	2
6	1
7	6
8	0
9	3
10	7

Having support from the university ()
Total
Challenges - Having support from the university

Response	Count
Seen unanswered	11
Unfinished	20
Valid skip	14
0	1
1	2
2	3
3	2
4	1
5	0
6	1
7	1
8	4
9	2
10	5

Having enough support from NE CASC leadership ()

Total

Challenges - Having enough support from NE CASC leadership

Response	Count
Seen unanswered	13
Unfinished	20
Valid skip	14
0	3
1	1
2	4
3	1
4	2
5	2
6	1
7	0
8	2
9	1
10	3

Having support from a supervisor ()

Total

Challenges - Having support from a supervisor

Response	Count
Seen unanswered	11
Unfinished	20
Valid skip	14
0	2
1	5
2	7
3	1

4	1
5	3
6	0
7	1
8	1
9	0
10	1

Sustainability of DEIJ programming over multiple years ()

Total

Challenges - Sustainability of DEIJ programming over multiple years

Response		Count
Seen unanswered		1
Unfinished		20
Valid skip		14
	0	0
	1	0
	2	0
	3	3
	4	1
	5	7
	6	1
	7	4
	8	5
	9	4
	10	7

Figure 8) What can NE CASC do to support DEIJ efforts?

**Figure 8A-E**	What can the NE CASC do to better support you and your DEIJ efforts? What are the challenges you are most concerned about when it comes to moving forward with your own DEIJ efforts? Please move the slider for each challenge on a scale of 0 (not important at all) to 10 (most important).

Fiugure 8A **Provide funding to support existing efforts**

Total

NE CASC can do(DEIJ efforts) - Provide funding to support existing efforts

Response	Count
Seen unanswered	6
Unfinished	20
Valid skip	14
0	1
1	0
2	1
3	2
4	2
5	5
6	1
7	2
8	3
9	3
10	7

Marginalized vs. Not-Marginalized

	Marginalized	No	Other
Seen unanswered	1	4	1
Unfinished	0	0	20
Valid skip	0	0	14
0	0	1	0
1	0	0	0
2	0	1	0
3	1	1	0
4	0	2	0
5	2	3	0
6	0	1	0
7	0	2	0
8	1	2	0
9	0	3	0
10	5	2	0

Fiugure 8B **Provide additional funding for undergraduate students**

Total

NE CASC can do(own DEIJ efforts) - Provide additional funding for undergraduate students

Response	Count
Seen unanswerec	10
Unfinished	20
Valid skip	14
0	1
1	0
2	3
3	1
4	1
5	4
6	2
7	2
8	3
9	1
10	5

Marginalized vs. Not-Marginalized

	Marginalized	No	Other
Seen unanswered	2	7	1
Unfinished	0	0	20
Valid skip	0	0	14
0	0	1	0
1	0	0	0
2	0	3	0
3	0	1	0
4	0	1	0
5	2	2	0
6	0	2	0
7	1	1	0
8	1	2	0
9	1	0	0
10	3	2	0

Fiugure 8C **Create clearer DEIJ policies**

Total

NE CASC can do(own DEIJ efforts) - Create clearer DEIJ policies

Response	Count
Seen unanswerec	9
Unfinished	20
Valid skip	14
0	1
1	1
2	0
3	1
4	0
5	7

6	1
7	2
8	3
9	1
10	7

Marginalized vs. Not-Marginalized

	Marginalized	No	Other
Seen unanswered	1	7	1
Unfinished	0	0	20
Valid skip	0	0	14
0	0	1	0
1	1	0	0
2	0	0	0
3	0	1	0
4	0	0	0
5	2	5	0
6	1	0	0
7	1	1	0
8	2	1	0
9	0	1	0
10	2	5	0

Fiugure 8D **Provide funding to support professional development opportunities**

Total

NE CASC can do(own DEIJ efforts) - Provide funding to support professional development opportunities

Response	Count
Seen unanswerec	10
Unfinished	20
Valid skip	14
0	2
1	1
2	1
3	2
4	0
5	3
6	2
7	5
8	3
9	2
10	2

Marginalized vs. Not-Marginalized

	Marginalized	No	Other
Seen unanswered	2	7	1

Unfinished	0	0	20
Valid skip	0	0	14
0	1	1	0
1	1	0	0
2	1	0	0
3	0	2	0
4	0	0	0
5	0	3	0
6	1	1	0
7	2	3	0
8	1	2	0
9	1	1	0
10	0	2	0

Fiugure 8E Provide additional funding for graduate students

Total

NE CASC can do(own DEIJ efforts) - Provide additional funding for graduate students

Response	Count
Seen unanswerec	6
Unfinished	20
Valid skip	14
0	2
1	0
2	2
3	1
4	1
5	3
6	4
7	2
8	5
9	0
10	7

Marginalized vs. Not-Marginalized

	Marginalized	No	Other
Seen unanswered	2	3	1
Unfinished	0	0	20
Valid skip	0	0	14
0	0	2	0
1	0	0	0
2	0	2	0
3	0	1	0
4	0	1	0
5	2	1	0
6	2	2	0
7	1	1	0

8	0	5	0
9	0	0	0
10	3	4	0
