# Supplementary material for: Centering voices of scientists from marginalized backgrounds to understand experiences in climate adaptation science and inform action
Source: PLoS One. 2025 Feb 21;20(2):e0318438. doi: 10.1371/journal.pone.0318438 (PMC11844896; doi:10.1371/journal.pone.0318438)
Supplement: Attachment — Submitted filename: R1_PONE-D-24-07943 2.pdf [file pone.0318438.s004.pdf]

PLOS ONE

Centering voices of scientists from marginalized backgrounds to understand experiences in climate adaptation science and inform action

--Manuscript Draft--

Manuscript Number:	PONE-D-24-07943R1
Article Type:	Research Article
Full Title:	Centering voices of scientists from marginalized backgrounds to understand experiences in climate adaptation science and inform action
Short Title:	Centering marginalized voices in climate adaptation
Corresponding Author:	Meghna N. Marjadi, PhD<br>University of Massachusetts Amherst<br>New Bedford, MA UNITED STATES OF AMERICA
Keywords:	diversity; equity; inclusion; DEI; climate survey; underrepresented scientists
Abstract:	Identifying and building solutions to help people and ecosystems adapt to climate change requires participation of all people. Unfortunately, Science, Technology, Engineering, and Mathematics (STEM) fields, including environmental sciences, continue to lack diversity. To address this issue, many institutions have increased programming to recruit and retain people from historically marginalized backgrounds into STEM fields. Institutions use surveys to evaluate the experiences of community members and identify areas for improvement; however, surveys often summarize and reflect majority perspectives and disregard voices of historically marginalized individuals. In June 2021, we surveyed graduate students, postdocs, faculty, staff, and researchers affiliated with the Northeast Climate Adaptation Science Center (NE CASC) to evaluate their experiences of diversity, equity, and inclusion (DEI) using Likert-based and long-answer questions. We analyzed the results as a whole, but also focused on the responses of people who self-identified as members of a marginalized group ("marginalized respondents") in climate adaptation science to center their voices. Marginalized respondents reported being motivated to enter climate adaptation science to improve society and the environment rather than for intellectual curiosity, which motivated one third of non-marginalized respondents. Once in science, marginalized respondents reported feeling less supported and comfortable at work and were more likely to have considered leaving science and academia in the last year. Long-answer responses of marginalized respondents indicated distrust in the ability of leadership and existing DEI initiatives to effectively tackle systemic issues and emphasized the importance of focusing on equity and inclusion before recruitment. Marginalized respondents identified additional funding to support existing DEI efforts and undergraduates as priorities. By allowing participants to self-identify as part of a marginalized group, we were able to highlight experiences and needs without risking exposure based on race, gender, disability, or sexual orientation. This approach can be applied to other small organizations with limited demographic diversity.
Order of Authors:	Meghna N. Marjadi, Ph.D.
	Rebecca A. Smith, Ph.D.
	Hsin Fei Tu, Ph.D.
	Asha M Ajmani, M.Sc.
	Addie Rose Holland, M.Sc.
	Bianca E Lopez, Ph.D.
	Toni Lyn Morelli, Ph.D.
	Bethany A. Bradley, Ph.D.
Response to Reviewers:	Reviewer #1:<br>1.This is a sound manuscript that adequately presents gaps and data that support the conclusions stated.<br>a.Thank you

Powered by Editorial Manager® and ProduXion Manager® from Aries Systems Corporation

	2.The title is informative and contains relevant and meaningful keywords.<br>a.Thank you<br>3.The final sentence of lines 97-99 is missing a reference, but the introduction adequately summarizes the existing knowledge about the topic and gaps that exist in the literature.<br>a.Thank you. We have added references to support this sentence.<br>4.Study aims are clear though the hypothesis could be framed more clearly.<br>a.We feel that the question-based framing at the end of the introduction is most appropriate for this study because we did not have hypotheses about how community members were experiencing and interacting with NE CASC.<br>5.The methodology while mostly clear needs clarification. What methodology was used for the qualitative coding (line 202)?<br>a.We followed grounded theory methodology to complete our coding. We have added more details to the qualitative analysis section of the results.<br>6.Also, please elaborate on why the Kruskal-Wallis rank sum test was used (line 204).<br>a.We used the Kruskal-Wallis test to compare the numbers of responses in each category for the marginalized and non-marginalized groups. We used this non-parametric test to accommodate our small dataset which had an unequal distribution across groups. We have added clarification in this section of the methods.<br>7.The results and discussion were clear and all figures appeared accurate and were appropriately descriptive.<br>a.Thank you<br><br><br>Reviewer #2: This study provided DEI insights in an academic institution setting. A survey was conducted with the Northeast Climate Adaptation Science Center consortium institutions to understand challenges faced by their community members, especially those from underrepresented groups. The study includes some interesting results related to the different motivations presented in the marginalized and non-marginalized groups. The paper can benefit from improvements in the following areas:<br>1.The introduction section only introduced the importance of understanding diversity in scientific and academic spaces, but failed to demonstrate the knowledge gaps in the area, and how this current study fills in these knowledge gaps. A literature review of similar previous studies in understanding DEI issues in scientific settings is completely missing. What approach has been taken by previous studies in understanding DEI issues, and what did they find as barriers to retention of scientists from historically marginalized backgrounds?<br>a.We focused on describing study results in our introduction rather than comparing methodologies. We have included an additional paragraph (paragraph 8) in the introduction to clarify the methods for some of the studies we cited earlier in the introduction. We have discussed barriers to retention in paragraph 6 and 7 of the introduction. We have also discussed these barriers in the context of our results in the discussion. Other surveys have been campus wide, undergraduate focused, or conducted across many STEM disciplines. Our study is unique in three ways: 1) focus on a smaller community with similar research goals and 2) consideration of different career levels, 3) focusing on marginalized communities with an intersectional lens.<br>2.The introduction also seems to misalign with the actual research conducted. The latter has an emphasis of career stage and age diversity in addition to race and gender diversity, which was mainly discussed in the introduction section. Similarly, the “barriers to retention of scientists from historically marginalized backgrounds” mentioned as a goal in the introduction section isn’t really addressed by this research.<br>a.We agree that the introduction focuses on race and gender diversity - this was intentional given that our results show that experiences of NE CASC community members primarily differed based on whether respondents identified as part of a marginalized group or whether they identified as cis-women. In the former case, people from marginalized groups tended to feel less supported and were more likely to have considered leaving science. In the latter case, cis-women also tended to feel less supported. Because we did not see consistent differences based on age or career stage, we elected not to frame the introduction based on these identifiers.<br>i.Our interpretation of the results related to whether respondents feel accepted and supported in the NE CASC community (Fig 2) and in science more generally (Fig 3) is that these feelings of inclusion are directly related to retention of people from historically marginalized groups. We have added a paragraph to the introduction to better connect feelings of support and belonging to retention of scientists in STEM.

	3.Was the survey able to engage any of the tribal members involved in the Center? What specific efforts have been made to engage tribal members in the survey, especially given the Center's emphasis on regional tribal nations?<br>a.One participant at the center identified as Indigenous American (as reported in the demographic section). We do not have demographic information for all people affiliated with the center, so we do not know how many Indigenous people did not participate. For this survey, we aimed to engage all members of the center equally to better understand experiences of the NE CASC community from all backgrounds. Much of the past and ongoing Tribal engagement work involves working with Tribal Nations and organizations whose members are not part of the NE CASC community (i.e. not directly supported with funding from the Center). However, partner organizations were not included in the survey.<br>4.What kinds of DEI initiatives have already been carried out by the Center? What is the Center's vision related to DEI? What motivated the survey? This kind of background is important for the readers to understand the context of and the responses to the survey.<br>a.This survey was among the initial DEI initiatives conducted by the Center. The survey was conducted in spring 2021 and the initial funding of 'DEI Fellows' began in fall 2020. The survey was motivated by the online workshop held in December 2020, where a discussion among community members revealed that baseline data about demographics and experiences in science and at NE CASC were necessary to evaluate whether future DEI actions had any effect. We have added background text on NE CASC's DEI activities and motivation to the beginning of the survey development and implementation section.<br>5.Figure 1: This figure should include breakdowns in terms of age and career stage, as these are important groups being discussed in the subsequent analyses. Similarly, the gender figure needs to have breakdowns of cis-male and female.<br>a.Thanks for this suggestion. We have expanded Figure 1 to include the breakdowns of age, career stage, and gender.<br>6.What constituents disabled? Physical or mental? This is not clear.<br>a.We included all types of disabilities in this category. The question in the survey was: "Please indicate any disability status that you may have" and the answers were: "Yes I have a disability" and "No I do not have a disability". We have included a clarifying sentence in the Demographic Information section of the Methods.<br>7.The narrative in the results section repeats the information that has already been presented in the figures. It is more important to discuss the analysis and the implications in the results, rather than simply restating the figures.<br>a.While some results text and figures might be redundant, we prefer to keep the results text because it allows us to include all of the p values associated with the Kruskal-Wallis tests. The format of Plos ONE has a separate results and discussion section, so we have discussed the analysis and implications in the discussion section.<br>8.What are the implications of the differences in terms of motivation for pursuing climate adaptation science? How does it relate to the retention of the underrepresented climate scientists?<br>a.Scientists from marginalized groups who have social motivations for their climate adaptation research may leave the field if they do not have the resources or support to do that research or if the field does not recognize the importance of social motivations in climate adaptation research. Other research (Schusler et al. 2021) has demonstrated that differences in motivations may contribute to students in environmental science feeling unwelcome and potentially leaving the field. Based on our results, this could also happen for researchers at later stages in their careers. Additionally, traditional funding mechanisms may also reduce opportunities to conduct research with social implications and motivations. We have revised this paragraph in the discussion to make the connections clearer.<br>9.How does the support for community members influence retention? What are the underlying reasons for the marginalized groups to feel less supported?<br>a.Researchers who identified as marginalized were more likely to feel less supported than their non-marginalized peers and also more likely to have considered leaving academia in the last year. While we cannot derive causation, lack of support may be related to considering leaving the field. To make this connection clearer, we have revised the paragraph that starts: "Discomfort and distrust, in addition to systemic inequities, may be driving marginalized scientists out of the field." Additionally, within our own study we do not have long term data to track whether participants left CASC or the field and the survey was anonymous.

	b.We have discussed some of the underlying reasons that scientists may not feel supported in our discussion and literature review. For example, if they feel their motivations for research do not align with department goals. Our survey goal was to assess how community members felt at the center. Some of the underlying issues are related to systemic problems in academia and science, which we have discussed in both the introduction and discussion.<br><br>10.What does “commitment to DEI” entail? What kind of commitment already exists? This is the part that really needs some background information about the institutional context of the NE CASC from the DEI perspective.<br>a.This is a good question - respondents might have interpreted this question in different ways. Based on open ended responses, it is likely that commitment to DEI was often interpreted as support for activities that build inclusivity, support for trainings and other opportunities that help people learn about diverse perspectives on science, and a long-term commitment to continue to support such activities. We added text to the introduction (in response to comment 4) to better describe the DEI activities supported by NE CASC leading up to this survey - these activities were focused on funding for ‘DEI Fellows’, whose efforts led to this survey and many years of Tribal engagement. NE CASC had written a response condemning the murder of George Floyd, but did not have another stated commitment to DEI prior to this survey. We have added some interpretation of ‘commitment to DEI’ to the discussion.<br>11.“Funding undergraduate students” is hard to understand again without the context. Is there a high percentage of unpaid undergraduate researchers within NE CASC currently? If so, what are the reasons for these students not to be compensated?<br>a.We have added additional context in the discussion paragraph that starts “Scientists from marginalized groups ranked funding for undergraduate students as a top priority, while scientists from non-marginalized groups ranked funding for undergraduate students as their lowest priority and ranked DEI policy as their top priority.” Unpaid undergraduate opportunities are prevalent in environmental and natural sciences due to funding limitations and the perception among some researchers that they are a ‘rite of passage’. Recruiting diverse undergraduate students contributes to a more diverse workforce and can be difficult when many unpaid opportunities exclude diverse researchers. We have rearranged the paragraph and added more context to explain this issue.
**Additional Information:**	
**Question**	**Response**
**Financial Disclosure**<br><br>Enter a financial disclosure statement that describes the sources of funding for the work included in this submission. Review the submission guidelines for detailed requirements. View published research articles from *PLOS ONE* for specific examples.<br><br>This statement is required for submission and **will appear in the published article** if the submission is accepted. Please make sure it is accurate.	Yes

Powered by Editorial Manager® and ProduXion Manager® from Aries Systems Corporation

**Funded studies**<br>Enter a statement with the following details:<br>• Initials of the authors who received each award<br>• Grant numbers awarded to each author<br>• The full name of each funder<br>• URL of each funder website<br>• Did the sponsors or funders play any role in the study design, data collection and analysis, decision to publish, or preparation of the manuscript?<br><br>Did you receive funding for this work?	
Please add funding details.<br>as follow-up to "**Financial Disclosure**<br><br>Enter a financial disclosure statement that describes the sources of funding for the work included in this submission. Review the submission guidelines for detailed requirements. View published research articles from *PLOS ONE* for specific examples.<br><br>This statement is required for submission and **will appear in the published article** if the submission is accepted. Please make sure it is accurate.<br><br>**Funded studies**<br>Enter a statement with the following details:<br>• Initials of the authors who received each award<br>• Grant numbers awarded to each author<br>• The full name of each funder<br>• URL of each funder website<br>• Did the sponsors or funders play any role in the study design, data collection and analysis, decision to publish, or preparation of the manuscript?<br><br>Did you receive funding for this work?"	This research was supported in part by the U.S. Geological Survey, Northeast Climate Adaptation Science Center (NE CASC) through Grant No. G19AC00091. Additionally, MNM was supported by a Switzer Foundation fellowship while working on this project and is currently supported by NOAA Cooperative Institute for the North Atlantic Region (Grant No. NA19OAR4320074).
Please select the country of your main research funder (please select carefully as in some cases this is used in fee calculation).	UNITED STATES - US

as follow-up to "**Financial Disclosure**<br><br>Enter a financial disclosure statement that describes the sources of funding for the work included in this submission. Review the submission guidelines for detailed requirements. View published research articles from *PLOS ONE* for specific examples.<br><br>This statement is required for submission and **will appear in the published article** if the submission is accepted. Please make sure it is accurate.<br><br>**Funded studies**<br>Enter a statement with the following details:<br>• Initials of the authors who received each award<br>• Grant numbers awarded to each author<br>• The full name of each funder<br>• URL of each funder website<br>• Did the sponsors or funders play any role in the study design, data collection and analysis, decision to publish, or preparation of the manuscript?<br><br>Did you receive funding for this work?"	
**Competing Interests**<br><br>Use the instructions below to enter a competing interest statement for this submission. On behalf of all authors, disclose any competing interests that could be perceived to bias this work—acknowledging all financial support and any other relevant financial or non-financial competing interests.<br><br>This statement is required for submission and **will appear in the published article** if the submission is accepted. Please make sure it is accurate and that any funding sources listed in your Funding Information later in the submission form are also declared in your Financial Disclosure statement.	This was a community based research project. All the authors have or are members of the NECASC community and have been either directly or indirectly funded by the center to complete this study or other research. The authors do not declare any other conflicts of interest related to this publication.

View published research articles from *PLOS ONE* for specific examples.<br><br>**NO authors have competing interests**<br><br>Enter: *The authors have declared that no competing interests exist.*<br><br>**Authors with competing interests**<br><br>Enter competing interest details beginning with this statement:<br><br>*I have read the journal's policy and the authors of this manuscript have the following competing interests: [insert competing interests here]*<br><br>* typeset	
**Ethics Statement**<br><br>Enter an ethics statement for this submission. This statement is required if the study involved:<br><br>• Human participants<br>• Human specimens or tissue<br>• Vertebrate animals or cephalopods<br>• Vertebrate embryos or tissues<br>• Field research<br><br>Write "N/A" if the submission does not require an ethics statement.<br><br>General guidance is provided below. Consult the submission guidelines for detailed instructions. **Make sure that all information entered here is included in the Methods section of the manuscript.**	The survey was conducted in accordance with the University of Massachusetts Internal Review Board (no. 2705). Survey consent from IRB was obtained by written application. Survey participants consented to participation on the first page of the survey after reading some information about the survey.

**Format for specific study types**<br><br>**Human Subject Research (involving human participants and/or tissue)**<br>• Give the name of the institutional review board or ethics committee that approved the study<br>• Include the approval number and/or a statement indicating approval of this research<br>• Indicate the form of consent obtained (written/oral) or the reason that consent was not obtained (e.g. the data were analyzed anonymously)<br><br>**Animal Research (involving vertebrate animals, embryos or tissues)**<br>• Provide the name of the Institutional Animal Care and Use Committee (IACUC) or other relevant ethics board that reviewed the study protocol, and indicate whether they approved this research or granted a formal waiver of ethical approval<br>• Include an approval number if one was obtained<br>• If the study involved *non-human primates*, add *additional details* about animal welfare and steps taken to ameliorate suffering<br>• If anesthesia, euthanasia, or any kind of animal sacrifice is part of the study, include briefly which substances and/or methods were applied<br><br>**Field Research**<br><br>Include the following details if this study involves the collection of plant, animal, or other materials from a natural setting:<br>• Field permit number<br>• Name of the institution or relevant body that granted permission	
**Data Availability**<br><br>Authors are required to make all data underlying the findings described fully available, without restriction, and from the time of publication. PLOS allows rare exceptions to address legal and ethical concerns. See the PLOS Data Policy and FAQ for detailed information.	No - some restrictions will apply

A Data Availability Statement describing where the data can be found is required at submission. Your answers to this question constitute the Data Availability Statement and **will be published in the article**, if accepted.<br><br>**Important:** Stating 'data available on request from the author' is not sufficient. If your data are only available upon request, select 'No' for the first question and explain your exceptional situation in the text box.<br><br>Do the authors confirm that all data underlying the findings described in their manuscript are fully available without restriction?	
**Describe where the data may be found in full sentences. If you are copying our sample text, replace any instances of XXX with the appropriate details.**<br><br>• If the data are **held or will be held in a public repository**, include URLs, accession numbers or DOIs. If this information will only be available after acceptance, indicate this by ticking the box below. For example: *All XXX files are available from the XXX database (accession number(s) XXX, XXX.*).<br>• If the data are all contained **within the manuscript and/or Supporting Information files**, enter the following: *All relevant data are within the manuscript and its Supporting Information files.*<br>• If neither of these applies but you are able to provide **details of access elsewhere**, with or without limitations, please do so. For example:<br><br>*Data cannot be shared publicly because of [XXX]. Data are available from the XXX Institutional Data Access / Ethics Committee (contact via XXX) for researchers who meet the criteria for access to confidential data.*<br><br>*The data underlying the results presented in the study are available from (include the name of the third party*	We have shared our survey in the appendices and our methods for survey analysis in the main text. We are unable to share raw data and metadata from this project since it is a survey that included demographic information which could identify individuals and sharing of such data would violate our IRB approval. We have shared anonymized data for our analyses and figures in a supplemental file.

and contact information or URL).<br>• This text is appropriate if the data are owned by a third party and authors do not have permission to share the data.<br><br>* typeset	
Additional data availability information:	

Manuscript

Click here to access/download;Manuscript;Marjadi_et_al_PloS_MainBody_Re

Centering voices of scientists from marginalized backgrounds to understand experiences in climate adaptation science and inform action

Short title: Centering marginalized voices in climate adaptation

Meghna N. Marjadi[1,5,#a*], Rebecca A. Smith[2,5], Hsin Fei Tu[3], Asha M. Ajmani[4,5], Addie Rose Holland[5,8], Bianca E. Lopez[4,5,6], Toni Lyn Morelli[4,1,7], Bethany A. Bradley[4,5]

[1]Graduate Program in Organismic and Evolutionary Biology, University of Massachusetts, Amherst, MA, 01003;

[2]Department of Geosciences, University of Massachusetts, Amherst, Massachusetts, United States of America

[3]Department of Sociology, University of Massachusetts, Amherst, Massachusetts, United States of America

[4]Department of Environmental Conservation, University of Massachusetts, Amherst, Massachusetts, United States of America

[5]Northeast Climate Adaptation Science Center, University of Massachusetts, Amherst, Amherst, Massachusetts, United States of America

[6]American Association for the Advancement of Science, 1200 New York Ave, NW Washington, District of Columbia, United States of America

[7]U.S. Geological Survey, Northeast Climate Adaptation Science Center, Amherst, Massachusetts, United States of America

[8]Center for Braiding Indigenous Knowledges and Science, University of Massachusetts, Amherst, Amherst Massachusetts, United States of America

[#a]Current: School of Marine Sciences and Technology, University of Massachusetts, Dartmouth, New Bedford, Massachusetts, United States of America

*Corresponding author
E-mail: mmarjadi@umassd.edu (MNM)

Keywords: diversity; equity; inclusion; DEI; climate survey; underrepresented scientists;

Abstract

Identifying and building solutions to help people and ecosystems adapt to climate change requires participation of all people. Unfortunately, Science, Technology, Engineering, and Mathematics (STEM) fields, including environmental sciences, continue to lack diversity. To address this issue, many institutions have increased programming to recruit and retain people from historically marginalized backgrounds into STEM fields. Institutions use surveys to evaluate the experiences of community members and identify areas for improvement; however, surveys often summarize and reflect majority perspectives and disregard voices of historically marginalized individuals. In June 2021, we surveyed graduate students, postdocs, faculty, staff, and researchers affiliated with the Northeast Climate Adaptation Science Center (NE CASC) to evaluate their experiences of diversity, equity, and inclusion (DEI) using Likert-based and long-answer questions. We analyzed the results as a whole, but also focused on the responses of people who self-identified as members of a marginalized group ("marginalized respondents") in climate adaptation science to center their voices. Marginalized respondents reported being motivated to enter climate adaptation science to improve society and the environment rather than for intellectual curiosity, which motivated one third of non-marginalized respondents. Once in science, marginalized respondents reported feeling less supported and comfortable at work and were more likely to have considered leaving science and academia in the last year. Long-answer responses of marginalized respondents indicated distrust in the ability of leadership and existing DEI initiatives to effectively tackle systemic issues and emphasized the importance of focusing on equity and inclusion before recruitment. Marginalized respondents identified additional funding to support existing DEI efforts and undergraduates as priorities. By allowing participants to self-identify as part of a marginalized group, we were able to highlight experiences and needs

without risking exposure based on race, gender, disability, or sexual orientation. This approach can be applied to other small organizations with limited demographic diversity.

Introduction

The environmental sciences suffer from a diversity problem, with lower proportions of Black, Indigenous, people of color (BIPOC), lesbian, gay, bisexual, transgender, or queer (LGBTQ+) people, people living with disabilities, and women relative to the general population (1–4). Retaining scholars from groups underrepresented in environmental sciences is a key aspect of addressing this lack of diversity (5) and many institutions have created surveys to understand how they can improve conditions and create a more equitable and inclusive workplace (6,7). Centering the voices of scientists from historically marginalized groups enables action that directly addresses barriers to an equitable and inclusive workplace. However, since people from historically marginalized groups comprise a small minority of the workplace for most environmental science fields (8,9), their needs may be overshadowed by majority opinion in averaged survey responses.

Environmental challenges are multifaceted and solutions require collaboration across disciplines and backgrounds (10). The need for diverse perspectives and collaborations is underscored by evidence that climate change has already and will continue to have the most detrimental effects on people from historically marginalized groups, who are often excluded from scientific and policy discussions (11). Environmental science fields, which include ecology, hydrology, climate science, and geosciences, are among the least diverse disciplines in Science, Technology, Engineering, and Mathematics (8,12), lacking both racial and LGBTQ+ diversity in both academic (1,4,9,13) and non-academic contexts (9,14,15). For example,

although racial and ethnic minorities comprised 29% of the US STEM workforce and 38% of the US population in 2018 (16), only 10.5% of the workforce in environmental science and conservation fields identified as members of a racial or ethnic minority group (5% Black, 0.7% Asian, 4.8% Latino/Hispanic) (17). Similarly, only 16% of staff in US environmental agencies, foundations, and nonprofits identified as non-white (9). While data are lacking for environmental fields specifically, LGBTQ+ people have been noted as underrepresented in STEM fields by an estimated 17-21% (13) and commonly face negative workplace experiences as STEM professionals (15,18).

Other demographics, like gender, national origin, religion, disability status, and financial background, also likely influence people's sense of belonging and long-term participation in STEM and environmental science fields. For example, cisgender women receive similar numbers of STEM PhDs as cisgender men, but represent only 24% of leadership positions in higher education (19). Similarly, while women comprise 60% of new hires in environmental organizations, men dominate top leadership positions across environmental organizations (9). Perhaps not surprisingly given their majority status, white, able-bodied, heterosexual men report feeling more included and respected, and have more career opportunities in STEM than people from all other racial, disability status, sexual orientation, and gender groups (14).

Further, scientific and academic spaces often fail to center intersectional identities; for example, a queer person of color with a disability may face discrimination that a straight or able-bodied person of color does not (20). Disparities are often heightened for women of color and other individuals who sit at the intersection of identities. For example, from 1973 to 2016, Native American, Black, Hispanic or Latino women together received only 1.46% of PhDs in geosciences (12). Similarly, Asian men hold twice as many faculty positions and three times as

many tenured faculty positions as Asian women, resulting in the largest gender disparity across all racial groups in academic environmental disciplines (4,21).

Lack of diverse representation in environmental sciences is not caused by lack of interest, but instead a lack of opportunity and support. Outdated and spurious claims that underrepresented students, especially BIPOC students, are disinterested in nature and the environmental sciences (22,23) are baseless and have been disproven in multiple studies (24–26). In one study, 91.2% of students from racial and ethnic minority groups reported feeling somewhat or very connected to nature (26). In fact, high percentages of BIPOC students report interest in working with federal environmental agencies and nonprofits (Taylor, 2018b). Although minority enrollment and performance in STEM fields are comparable to those of white students, a significant percentage of Asian, Black, Latino/Hispanic, and Native American students do not complete STEM degrees (17,27). In the environmental sciences, historical legacies of exclusion, hierarchical power dynamics, and/or unsafe fieldwork or learning conditions may contribute to students leaving STEM programs (28). Additionally, retention issues may reflect systemic failures of university environments and curricula to meet the needs of diverse student interests (5,29). Even when they do attain STEM degrees, students from racial and other minority groups face additional barriers as they pursue advanced degrees and move forward in their careers (28).

Attrition in the environmental science fields may be related to the many barriers to equity and inclusion, which include bias, discrimination, and harassment in both educational environments and the workplace (28). Within ecology and environmental sciences, students of color have reported discrimination, lack of relatability, and limited discussions of race as promoting feelings of isolation and exclusion in the environmental sciences (5). More broadly,

Black and Latino/Hispanic STEM undergraduate students experience microaggressions and other racist incidents that can cause lasting psychological impacts and reduce productivity, dampen interest, and ultimately cause students to change fields (30,31). Cantor et al. (7) reported that 59% of female undergraduate students in STEM fields in the U.S. have experienced harassment. Retention of members of historically marginalized communities in science begins with identifying and dismantling barriers at early career stages (32,33).

Feeling a sense of belonging – defined as “the feeling of security and support when there is a sense of acceptance, inclusion, and identity for a member of a certain group (6)” – has been associated with increased retention for students in STEM majors (32) and faculty across academic fields (33). For undergraduate students, representation within STEM subdisciplines, interpersonal relationships, perceived competence, and science identity contributed to feelings of belonging and the desire to stay in their majors (32). Similarly, a census study revealed that women left academic positions at higher rates than men and were more likely than men to leave positions because they felt “pushed out” or unsupported in their role, compared to men who were more likely to feel “pulled” to a better opportunity (33).

University campuses have employed institution-wide “climate surveys” to collect demographic information and assess workplace dynamics (34). Since surveys are often deployed across the entire institution (34,35), they do not account for department-specific dynamics (6). Similarly, many studies on belonging and retention in STEM have been conducted across disparate STEM fields (30,31,36) and may not reflect the unique experiences of researchers in environmental sciences, which includes some of the least diverse STEM fields (4,8,12). Many articles on diversity in the environmental sciences have used compilations of existing data to answer questions about demographic trends (1,2,9,12) but do not use survey or interview tools to

investigate the underlying reasons for those trends. Although a few studies have used interviews and surveys to address belonging and retention for undergraduate students in ecology and environmental sciences (5,26,27), no studies have been published in the peer-reviewed literature recently that evaluated graduate student, postdoctoral fellow, or faculty experiences at a departmental or programmatic level based on marginalized or underrepresented identity. ~~Barrile et al. (6) used a survey to~~ evaluate ~~experiences in a zoology department and considered differences in experiences~~ across c~~areer levels, but did not analyze demographic characteristic~~s. Evaluating departmental, programmatic, and field specific dynamics for researchers at different career stages may prove valuable in better understanding experiences of scientists from diverse backgrounds in environmental sciences.

doesn't really provide anything for the reader

We surveyed students, postdoctoral scholars, faculty, staff, and other researchers affiliated with the Northeast Climate Adaptation Science Center (NE CASC) to better understand their experiences in the program. Ultimately, we planned to use survey results to identify and address barriers to retention of scientists from historically marginalized backgrounds in the NECASC and in environmental science fields. The NECASC includes scientists in environmental sciences–including ecologists, hydrologists, social scientists, and geoscientists–with a focus on climate adaptation science and climate science research. Other DEI related studies in environmental science and ecology have been focused on undergraduate students (5,26,27,32) or professionals outside of academia (9).

As a cross-institutional network, the NE CASC represents only a small portion of most respondents' experiences in science. Academic departments, colleges, universities may play a more prominent role in student, postdoctoral scholar, and faculty experiences. Nevertheless,

actions of the center to improve equity and inclusion could support retention of community members from historically marginalized groups. We collected responses to a series of Likert scale and open-ended survey questions and listened to individual stories through workshops. This study aims to center and amplify the voices of individuals from historically marginalized backgrounds and focuses on questions related to 1) the motivation of people affiliated with the NE CASC (hereafter, NE CASC consortium members) for entering careers related to climate adaptation, 2) how supported community members feel as individuals and in their work, including their likelihood of leaving scientific research, and 3) barriers to and priorities for DEI action.

Methods

Case study site and community

The Climate Adaptation Science Centers (CASCs) are a partnership between the U.S. Geological Survey (USGS) and consortium institutions (including universities, Tribal organizations, government agencies, and non-governmental organizations) to develop science to help fish, wildlife, water, land, and people adapt to a changing climate. The NE CASC is hosted by the University of Massachusetts Amherst. As of June 2021, when this survey was conducted, the NE CASC consortium institutions included the University of Vermont, Cornell University, Woodwell Climate Research Center, USFS Northern Research Station, Columbia University, the College of Menominee Nation, University of Wisconsin, University of Missouri, and Michigan State University. The NE CASC community includes graduate students (both M.S. and Ph.D.), postdoctoral scholars, university faculty, tribal organization staff, federal and university

administrative staff, and research scientists. Between its inception in 2012 and when this survey was conducted in 2021, a total of 177 individuals have been associated with the NE CASC consortium (115 graduate students and postdoctoral scholars, collectively referred to as fellows, 43 funded principal investigators, and 19 staff). Graduate students and postdoctoral fellows join the NE CASC Fellows program when they are funded on a NE CASC project. Undergraduate students may support NE CASC projects, but few have formal involvement with the NE CASC. We grouped both currently and formerly affiliated community members to include voices spanning the NE CASC's existence. To guide work on retaining members of the community and making activities more equitable and inclusive, the NE CASC sought to understand challenges faced by their community members, especially those from underrepresented groups, and to identify priority areas in need of support.

Community-based participatory research process and survey development

Beginning in the fall semester of 2020, the NE CASC has supported new DEI initiatives by funding graduate student 'DEI Fellows', some of whom focused on understanding and improving the NE CASC's recruitment and retention of students and staff from underrepresented groups. Until that time, the NE CASC's DEI organizational goals and activities related largely to training early career scientists in ethical engagement principles, building engagement with regional Tribal Nations, prioritizing climate adaptation projects relevant to Tribal Nations, and including Native students in the NE CASC Fellows Program in partnership with a Tribal college (the College of Menominee Nation) and through the Bureau of Indian Affairs (BIA) Pathways

internships (NE CASC Tribal Nation Partners website, 2013-2018 NE CSC Strategic Science Agenda).

To begin understanding how the NE CASC could support new DEI initiatives, the DEI Fellows led a workshop on December 2, 2020, where community members identified and discussed DEI priorities. At the workshop, 22 members of the NE CASC community discussed the need for a survey to assess existing diversity, identify needs, and prioritize actions. As an outcome of the workshop, a community climate survey was developed and the NE CASC formed a DEI committee to plan and oversee diversity, equity, and inclusion efforts for the NE CASC. Members of the NE CASC community and DEI committee were involved in survey development and implementation.

Survey development and implementation

Our survey was motivated by the outcomes of the December workshop, where it was noted that NE CASC did not have any baseline data of existing diversity among community members, nor information about priority areas for future DEI work.. We created the initial survey with guidance from campus climate surveys that were freely available online, including surveys administered by the University of Massachusetts, Amherst, the University of Michigan, and the University of California, Los Angeles. We tailored our survey to the NE CASC consortium and included specific questions about climate adaptation research and the NE CASC's processes around recruitment, hiring, and mentoring (S1 Table). The NE CASC's leadership and DEI committee reviewed and tested the survey. The survey was implemented in Qualtrics and sent to the e-mail addresses of 177 consortium members who were previously or currently affiliated with the NE CASC (as of June 2021). The survey employed Likert scale,

multiple choice, and open answer questions (S1 Table) and was estimated to take about 30 minutes to complete. We began soliciting survey responses on June 4th, 2021 and accepted responses until July, 9th 2021. We sent three reminder emails to the community during that time. The survey was conducted in accordance with the University of Massachusetts Internal Review Board (no. 2705).

Demographic information

Although the survey was anonymous, we collected demographic information that could have made some individuals identifiable. To protect privacy, we worked with the University of Massachusetts Amherst's Institute for Social Science Research (ISSR) to anonymize the dataset and ensure that the NE CASC leadership only viewed the results in aggregate. Due to the small sample size, we were unable to split the dataset based on race, sexuality, or disability status without compromising the identities of respondents. All participants self-identified their demographic information and could answer yes or no to report if they had any sort of disability (either ~~mental or physical~~ visible or invisible). One of our survey questions asked respondents whether they identify as a member of a marginalized community in science (Yes, No). The group responding 'yes' (hereafter, marginalized) was large enough that individuals could not be identified and was used as our primary grouping for comparing their experiences to the experiences of people that responded 'no' (not marginalized).

Additional groupings included age (Younger: <45, Older: ≥45), gender (cisgender man, cisgender woman), and career stage (graduate student or postdoctoral (fellow), later-career faculty or staff (non-fellow)). We only considered self-reported demographic information. Thus, participants could only be grouped if they answered the corresponding demographic survey

questions, which were at the end of the survey and were skipped by some respondents. Responses lacking demographic data were included in the analysis of total responses but excluded in group comparisons. As a result, the number of total respondents to the survey and the number of participants included in grouped data differs by question.

Quantitative analysis

Likert-scale values were converted to numeric as follows: strongly disagree = 1, somewhat disagree = 2, neither agree nor disagree = 3, somewhat agree = 4, strongly agree = 5. We applied the non-parametric Mann-Whitney U-test to compare distributions of Likert-scores between groups. The Mann-Whitney U-test is appropriate because the data are not normally distributed, groups are unequally sized, and sample sizes are small (37). Due to low statistical power in small sample sizes, we only report statistical results for questions that had at least 8 responses for each category tested (38). Each question was analyzed independently. We interpreted a p-value ≤ 0.05 as a significant difference between groups and a p-value ≤ 0.10 as a somewhat significant difference between groups. Plots were created in R computing language (39) using the ggplot2 package (40).

Qualitative Analysis

We conducted a qualitative analysis for three of the open-~~answered~~ ended questions in the survey, for which we had received adequate numbers of responses. We followed grounded theory methodology to complete our coding (41). Three researchers worked on examining short responses, identifying themes, and assigning representative codes to those themes. We then met as a group to discuss coding approaches, find overlapping themes and codes, and identify which

themes and codes best represented the data. We then re-coded each of the responses into the new codes. We completed our coding in Microsoft Excel spreadsheets.

To better understand the motivations of the NE CASC community for entering the field of climate adaptation science, we asked the open ended question: “Why did you choose to pursue climate adaptation research?”. We coded responses into categories without demographic information; an associate at the ~~ISSR~~ later added the demographic information to the responses.

To compare motivations between (self-identified) marginalized and non-marginalized groups, we used a Kruskal-Wallis rank sum test to test differences in the numbers of responses in each category for the two groups (35). The Kruskal-Wallis test was appropriate because our dataset was small and the groups were uneven.

To better understand concerns about DEI actions supported by the NE CASC moving forward, we asked the question: “What is your biggest concern for the NE CASC as it moves forward with DEI initiatives?” To focus on the needs of people who identified as marginalized, we asked: “What can the NE CASC do to support you as a member of a marginalized or underrepresented group?” We did not analyze these qualitative data statistically, but instead used the responses to illustrate the concerns and needs of community members who identified as marginalized.

Results

Demographics of the NE CASC community

We collected 42 responses to the survey (a 24% response rate). Most respondents (28; 67%) were affiliated with NE CASC at the time of the survey, with the remaining (14, 33%)

being previously affiliated. Overall, 32 (76%) respondents answered the question of whether they self-identified as a member of a marginalized group; of these, 10 (30%) identified as marginalized and 22 (67%) did not identify as marginalized; the remaining survey respondents did not answer this question (10; 24%) (Fig 1). We collected more responses from cisgender women (22; 52%) than cisgender men (16; 38%), with some respondents choosing to skip the question about gender (3; 7%) or selecting that they preferred not to respond (1; 2%). The majority of respondents identified as white (35; 83.2%), with the remaining respondents identifying as Indigenous American (1; 2.4%), South Asian (1; 2.4%), Black (1; 2.4%), Latino/Hispanic (2; 4.8%), and mixed race (1; 2.4%); the remaining respondents (1; 2.4%) did not complete the demographic proportion of the survey. The majority of respondents identified as not having a disability (33; 79%) and as heterosexual (33; 79%) (Fig 1). As this is the first time the NE CASC has collected self-reported demographic data, we have no other demographic data from the NE CASC to compare our responses to the overall community.

Fig 1: Demographic breakdown of respondents to the survey. The majority of respondents identified as A) white, B) not disabled, and C) heterosexual. D) Ten respondents self-identified as being a member of a marginalized or underrepresented group in climate adaptation science. In addition to considering responses based on marginalized vs. non-marginalized groups, we were also able to compare responses for E) older vs. younger, F) fellows (students and postdocs) vs. non-fellows (PIs and staff), and G) cis-men vs. cis-women (the only two gender categories specified in the demographic responses).

Motivation for pursuing climate adaptation science

Our qualitative analysis of the question of personal motivation to pursue climate adaptation science yielded three major categories of reasoning for pursuing climate adaptation research. 1) “Research Interest or Intellectual Curiosity" included responses that were focused on academic research impacts or topical interest and had no connections to specific social or conservation implications (e.g., “It is fundamental to my research interests”; “It was a natural progression of my education and interests”). 2) “Environmental Conservation and Management” included responses that focused on concerns for the future of the planet, conserving resources, and preserving biodiversity (e.g., “I believe that climate change is one of the biggest threats to non-human species in millions of years”; “I am passionate about my research system (forests), and climate change poses a threat to the persistence of these ecosystems”). 3) “Societal Impact” captured responses that considered relationships between climate adaptation and human wellbeing, including references to community and human welfare (e.g., “It impacts my community, family, and future”; “Because it is a way I can use my scientific knowledge to aid others”; “Because it impacts all areas of research and livelihoods”).

People who identified as marginalized had somewhat significantly different responses from people who identified as not marginalized (Kruskal-Wallis, $p = 0.052$, Fig 2). No respondents from the marginalized group reported a motivation stemming from research or intellectual curiosity alone. There were no significant differences between other groups (e.g., age, career stage, gender).

Fig 2. **Participant motivations for pursuing climate adaptation science.** Reasons for pursuing climate adaptation science were grouped into three categories. There was a somewhat significant

difference in responses between people who identified as marginalized and people who identified as not marginalized (Kruskal-Wallis, p = .052). n refers to the sample size of each group.

Support for community members

Acceptance in science and interactions within the scientific community

Overall, the majority of respondents reported feeling supported in the NE CASC community; however, our results indicated that respondents who identify as marginalized have less positive experiences (Fig 3). Respondents strongly or somewhat agreed with the statements “My identity and background are/were supported and accepted in the NE CASC community” (Fig 3A), “NE CASC members with different backgrounds interact well with each other” (Fig 3F), and “I felt comfortable and safe being myself within the NE CASC community” (Fig 3K). However, community members who identified as marginalized reported less agreement with all three statements (*p* = 0.003, Fig 3B; *p* = 0.076, Fig 3G; *p* = 0.001, Fig 3L). Cisgender women reported less agreement than cisgender men, with significantly fewer “strongly agree” responses to the first and second statements (*p* = 0.050, Fig 3E; *p* = 0.033, Fig 3J). Early career fellows reported less agreement than non-fellows, with fewer “strongly agree” responses to the second and third statements (*p* = 0.071, Fig 3I; *p* = 0.044, Fig 3). There were no significant differences in agreement with age groups (Fig 3C, H, M).

Fig 3: Participant responses to questions related to acceptance within the NE CASC community. People who identified as marginalized, fellows, and women often reported feeling less supported, less comfortable with community interactions, and less comfortable being themselves. Significant differences (p < 0.05) are indicated with a *, somewhat significant

differences ($p < 0.10$) are indicated with a †. Numbers in boxplots indicate the total number of respondents. “Total” refers to all survey responses, regardless of demographic breakdown; “marg.” and “not marg.” refer to the self-identified marginalized and not-marginalized demographic responses; “<45 yr” and “≥45 yr” refer to the age breakdown; “fell.” and “non-fell.” refers to NE CASC fellows (graduate students and postdoctoral scholars) and non-fellows; “cis. w.” and “cis. m.” refers to the cisgender women and cisgender men demographic breakdowns, with no other genders self-identified in the survey.

Support for research, training, and engagement

Overall, our results indicated that NE CASC community members felt well supported. However, similar to responses related to acceptance, people who identified as marginalized felt significantly less supported as climate adaptation scientists in the NE CASC community (Fig 4). Respondents strongly or somewhat agreed with the statements “my research and research goals were supported by the NE CASC” (Fig 4A) and “my professional and professional development goals were supported by the NE CASC” (Fig 4F). Generally, respondents only somewhat agreed with the statement “my outreach and stakeholder engagement goals were supported by the NE CASC” (stakeholder engagement is a focus of NE CASC work; Fig 4K). In all cases, people who identified as marginalized were less likely to feel supported ($p = 0.019$, Fig 4B; $p = 0.092$, Fig 4G; $p = 0.035$, Fig 4L). Women also reported feeling significantly less supported than men in both research ($p = 0.015$, Fig 4E) and professional development ($p = 0.004$, Fig 4J).

Fig 4. **Responses to how well (A-E) research, (F-J) professional development, and (K-O) stakeholder engagement goals were supported by the NE CASC.** In all cases, people who

identified as marginalized were less likely to agree that they were supported. Significant differences ($p < 0.05$) are indicated with a *, somewhat significant differences ($p < 0.10$) are indicated with a †. Numbers in boxplots indicate the number of respondents.

Commitment to DEI from supervisors and leadership

Overall, respondents strongly or somewhat agreed that their direct supervisors and NE CASC leadership were committed to diversity, equity, and inclusion (Fig 5). While respondents strongly agreed that their supervisors were committed to DEI (Fig 5A), they only somewhat agreed that NE CASC leadership was committed to DEI (Fig 5K). Younger people (respondents <45 years old) and fellows were somewhat less likely to strongly agree that their direct supervisor was committed to DEI ($p = 0.097$, Fig 5C; $p = 0.075$, Fig 5D). There were no significant differences between other groups in terms of how well direct supervisors were perceived to handle matters of DEI (Fig 5G-J). People who identified as marginalized and fellows were significantly less likely to agree that NE CASC leadership is committed to DEI ($p = 0.016$, Fig 5L; $p = 0.022$, Fig 5N).

Fig 5. Respondent perceptions of direct supervisor commitment to diversity, equity, and inclusion (A-E), how well supervisors handled matters related to diversity, equity, and inclusion (F-J), and whether leadership was perceived as supporting diversity (K-O). People who identified as marginalized were significantly less likely to view diversity as a priority to leadership. Significant differences ($p < 0.05$) are indicated with a *, somewhat significant differences ($p < 0.10$) are indicated with a †. Numbers in boxplots indicate the number of respondents.

Likelihood of leaving scientific research or academia

Overall, respondents strongly disagreed that they have considered leaving academia or scientific research over the last year, including considering leaving due to their identity (Fig 6). Consistent with previous questions, people who identified as marginalized and fellows showed less disagreement, with the median response for people who identified as marginalized being somewhat agreeing with considering leaving science (p = 0.045; Fig 6B). People who identified as marginalized and fellows also showed less disagreement with the statement that they have considered leaving scientific research due to their identity (p = 0.022, Fig 6G; p < 0.001, Fig 6I).

Fig 6. Responses to whether respondents had recently considered leaving academia (A-E), including considering leaving due to their identity (F-J). People who identified as marginalized and fellows were significantly more likely to have considered leaving academia, including due to their identity. Significant differences (p < 0.05) are indicated with a *, somewhat significant differences (p < 0.10) are indicated with a †. Numbers in boxplots indicate the number of respondents.

Barriers and priorities for DEI action

Barriers

There were insufficient data to compare Likert scale responses to “What are the challenges you are most concerned about when it comes to moving forward with your own DEI efforts?” between groups. However, there was a consistent pattern in challenges to DEI efforts across all respondents; sustainability of DEI programming, available time, available funding, and support from the participant’s respective academic institution were generally reported as larger

challenges. Support from direct supervisors was reported as being a significantly smaller barrier than all other challenges except for support from NE CASC leadership (Fig 7).

Fig 7. Challenges faced by NE CASC community members for advancing their own DEI efforts ordered from largest to smallest. Box plots include all respondents (both marginalized and not marginalized) due to low sample sizes (white numbers). Letters at the top represent significant (a vs. b; $p<0.05$) or non-significant (a vs. a) differences in the size of the challenge.

In coding open answer responses to the question about concerns and challenges of institutional DEI work, we identified three major themes among answers from individuals who identified as marginalized. First, respondents were concerned about lack of capacity. This included concerns that graduate students of color shoulder the burden of DEI work in science fields; that there is an absence of planning for sustainable DEI work; and that there is a lack of available funding to support long-term DEI efforts. Second, respondents wrote that a focus on equity was equally or more important than a focus on increasing diversity, for example: "[My] biggest concern is that [institutions] will continue to focus on diversity, without making any progress on equity, inclusion, and justice. This translates into continuing to bring people from marginalized backgrounds into a hostile workplace/environment." Finally, distrust of institutions and current DEI work was seen in comments such as, "I'm not confident that the current members of NE CASC feel the urgency/have the capacity of improving their cultural competency in a timely manner that's relevant for current/future students", and "…the community are more interested in performative activism than actually dismantling the structural racism within the organization and within the greater climate science academic community."

Future priorities

Overall, responses to the question of future priorities for DEI efforts were low (n = 15 to 27, response rate varied by question based on priority subcategory). Respondents who identified as marginalized ranked “funding existing DEI efforts”, “funding undergraduate students”, and “including DEI efforts in job responsibilities” among their top priorities for future actions, whereas community members from non-marginalized backgrounds ranked “creating clearer DEI policy” as their top priority (Fig 8). “Funding undergraduate students” was a significantly higher priority for marginalized than non-marginalized groups ($p = 0.043$; Fig 8B). When comparing between priorities, people who identified as marginalized ranked “supporting professional development activities” lower than “funding existing DEI efforts” ($p = 0.064$) and “funding undergraduate students” ($p = 0.081$). There were no significant differences between priorities for people who did not identify as marginalized.

Fig 8. DEI priorities for marginalized and not-marginalized participants. Priorities for supporting DEI efforts ordered by median response by people who identified as coming from historically marginalized groups. Respondents who identified as coming from a historically marginalized group were significantly more likely to select the funding of undergraduate students as a high priority. Significant p-values are indicated with a *. Numbers in the boxplots indicate the number of respondents.

In response to an open-ended question asking people who identified as marginalized what they needed in order to feel more supported in their work, respondents focused on building capacity and equity. For example, respondents stated the NE CASC should “be clearer about

funding opportunities”, hire a professional DEI consultant, and “pay more for the cultural taxation of having to teach our over-represented colleagues.” To make the workplace more equitable and inclusive, one respondent suggested efforts to “train faculty and staff better in inclusivity to create a more welcoming environment.” Another response similarly highlighted the need to focus on equity before diversity, “I think too much of the focus of this work is about ‘how can we do better in the future,’ but that aspiration is not built on an adequate foundation of a deep understanding of what is causing minoritized people to struggle in climate adaptation science. NE CASC could do more to acknowledge the challenges faced by underrepresented groups, which includes acknowledging how our systems perpetuate those challenges.”

Discussion

Our survey shows that the NE CASC community as a whole feels supported. However, in both peeling away the majority perspective from Likert-based survey responses and ~~in~~ focusing on anonymous long-answer responses, this analysis highlights how community members from marginalized groups feel less welcomed and supported in environmental sciences. People who identified as marginalized reported significantly less positive experiences from their non-marginalized peers. I~~n particular,~~ people who identified as marginalized, women, and early career fellows reported less agreement that scientists from different backgrounds in the NE CASC community interacted well with each other. People who identified as marginalized and women reported less agreement that they felt their identities were accepted in the NE CASC community, ~~while~~ people who identified as marginalized and fellows reported less agreement that they felt comfortable being themselves in the NE CASC community. While NE CASC is

only a portion of most respondent's experience with science, these results clearly demonstrate that our science institutions are not creating the same experiences for all people.

Long-answer responses from survey participants indicated lack of trust in leadership and in existing DEI efforts to address issues of systemic racism. Further, responses identified concern that recruiting diverse community members would continue to be prioritized over efforts towards creating a more equitable, inclusive, and just workplace for current community members. Indeed, according to a synthesis of over forty years of publicly available demographic data in the geosciences across degree-levels, Bernard and Cooperdock (2018) highlighted that efforts focused primarily on recruitment and increasing diversity have failed to improve demographics of underrepresented minorities in the geosciences in four decades, and that programs need to revise strategies of improving diversity in the field (12). Previous research across scientific fields has shown that people from historically marginalized groups often experience uncomfortable working environments and have little trust in institutions to address systemic racism and inequities (Bernard & Cooperdock, 2018; Marín-Spiotta et al., 2020; Ruf, 2020; Schusler et al., 2021; Taylor, 2018), potentially indicating that efforts focused on recruitment often fail to address issues of diversity in the field by not addressing barriers to retention.

When asked how supported they felt in both their professional and service and outreach works, participants expressed differing perspectives. People ~~from marginalized backgrounds~~ and cisgender women both agreed less that their research goals and professional goals were supported when compared to non-marginalized scientists and cisgender men. However, cisgender women felt equally as supported as men in their service and outreach work, while marginalized people reported feeling less supported in outreach and service work. Service work is often expected of women, and may reduce how supported they feel ~~as scientists~~ (42). Climate

surveys of other ecology and STEM departments have also revealed perceptions among participants of unequal participation in service work (6,43). Within STEM departments, racially minoritized women are more likely than racially minoritized men to engage in diversity-related service work, and have reported feeling unsupported in this role (43). Despite comprising the majority of our respondents, cisgender women reported significantly lower feelings of acceptance and support for their research when compared to cisgender men. This information would be lost if representation (52% of survey respondents were women) was our only consideration in assessing community wellness. Our results demonstrate that representation does not preclude inequities among historically marginalized groups, suggesting that efforts focused solely on representation, recruitment, and increasing diversity may still fail to create equitable working conditions, which are crucial to retention of diverse scientists across a range of backgrounds.

While all respondents, both those who identified as marginalized and those who did not, expressed confidence that their direct supervisors were committed to diversity, equity, and inclusion, they were less confident about the commitment of institutional leadership to advancing DEI through actions. While ‘commitment to DEI’ is somewhat open to interpretation, based on written responses, this might have been interpreted as support for DEI actions and/or long-term commitment to work on breaking down barriers to a diverse, equitable, and inclusive scientific enterprise. For people from historically marginalized groups, distrust of leadership may stem from their historical exclusion from the ecology and environmental science fields (28,29), the field’s historical colonization of natural spaces and sciences without consideration for resident and indigenous perspectives and practices (29), leadership focus on performative rather than sustainable actions (43), and co-opting of grassroots efforts by administrators (43). Rebuilding

trust may require demonstrated actions by institutional leadership to support sustainable programming and long term commitment to inclusive and equitable spaces. This may include a focus on enhancing positive interactions with current faculty, staff, and researchers to foster inclusive environments (44,45). In their long answers, respondents suggested hiring a professional DEI consultant to support efforts, potentially to help create program plans. In a national study of environmental NGOs, organizations with developed program plans that included concrete goals, incentives, and community buy-in were more successful in their diversity outcomes (46). Thus, development of long-term activities that support diversity, equity, and inclusion in the NE CASC community should lead to more positive experiences in climate adaptation science and better retention of researchers and staff. For example, since this survey, NE CASC has set up a DEI committee that meets regularly; consistently funded graduate DEI fellows to build a more inclusive community; established a travel grant to support graduate students and postdoctoral scholars participation in conferences and workshops in order to increase DEI; and has co-produced a statement describing its long-term commitment and actions towards improving DEI.

Discomfort and distrust, in addition to systemic inequities, may be driving marginalized scientists out of the field. Building community and actively creating inclusive environments can help to increase belonging and ultimately retention (45,47). For example, interactions with near-peer and expert in-group mentors can help to increase retention of people from marginalized groups (36,48–50). Respondents who identified as marginalized were more likely than other groups to agree that they had considered leaving academia in the last year, although these respondents also disagreed that they had considered leaving due to their identity or interest. As we have noted above, scientists from marginalized backgrounds also felt less supported than

their non-marginalized peers. While we cannot derive causation, feeling less supported may contribute to scientists who identified as marginalized considering leaving academia. These community members may ~~essentially be saying "it's not me, it's *you*" and are~~ be considering leaving science and academia due to an unsupportive working environment or a failure of the field to support research goals and interests. For example, despite high levels of interest, capability, and enrollment in environmental science and ecology, undergraduate BIPOC students have high rates of attrition in these majors (17), which may be related to reported discrimination, feelings of isolation, and dissatisfaction with social perspectives and social consciousness in delivered curricula (5,29). While attrition was not quantified in this study, our results indicate that similar barriers may exist for graduate students, postdoctoral scholars, staff, and principal investigators that are part of the survey community of this study.

Our results suggest that socially minded priorities persist for marginalized scientists beyond undergraduate science courses. Shifting focus to applications of research that investigate impacts of environmental issues on humans and non-human beings, rather than abstract scientific "coolness", could help to attract scientists from more backgrounds (2). Respondents from marginalized groups were more likely to have socially minded reasons for pursuing climate and environmental science compared their non-marginalized peers. Incorporating these motivations into curriculum development, seminar series, and departmental discussions may improve inclusion of social impact interests and promote overall retention. Similar to Schusler et al. (5), we found that people from marginalized groups cited either social or conservation related reasons for pursuing their research rather than intellectual curiosity alone. In contrast, one-third of non-marginalized scientists cited intellectual curiosity as their primary reason for pursuing the field. Differences in priorities may lead to a misalignment in activities aimed at recruiting and

supporting people from marginalized groups in ecology and environmental sciences, which may prevent scientists from feeling professionally satisfied and prompt them to leave the field. Unfortunately, the history of ecology and environmental science has been highly colonial; natural spaces were often brutally seized from Indigenous peoples in the name of “exploration” and “scientific discovery” (29,51). For example, geologic mapping was a primary weapon of colonization, wherein resource maps provided by geologists to the U.S. government were used as intellectual weapons to justify the forced removal of Indigenous groups (despite evidence in colonizer geologic notes that Indigenous groups held superior knowledge in regional topography and ecology). The scientists who engaged in these colonial practices are often held up as trailblazers in required coursework (51). Thus, while people from marginalized groups share an interest in the ecology and environmental sciences, curriculum failures and others’ perceptions of who belongs in the field may deter diverse scientists from entering or staying in environmental sciences (29). As our and other studies show, marginalized scientists are more likely to have socially and community minded reasons for pursuing environmental research (5), but social and environmental justice disciplines have traditionally been considered separate from ecological and environmental departments (5,29), which means that marginalized scientists who are interested in climate and environmental science but also have social motivations may prefer departments with a stronger interdisciplinary research focus. BIPOC students and scholars have cited efforts to decolonize environmental science curricula and incorporate social perspectives as one way to address issues of inclusion (5,29). Our results suggest that socially minded priorities persist for marginalized scientists beyond undergraduate science courses. Shifting focus to applications of research to humans and non-human beings, rather than abstract scientific “coolness”, could help to attract scientists from more backgrounds (2).

Scientists from marginalized groups ranked funding for undergraduate students as a top priority, while scientists from non-marginalized groups ranked funding for undergraduate students as their lowest priority and ranked DEI policy as their top priority. Many NE CASC researchers supervise and rely on undergraduate students to support their research as research and field assistants, though few of these undergraduate researchers are formally involved in NE CASC activities. For undergraduate students, internships and technician experiences are crucial to advancement in natural science careers as these experiences are often considered in admission to graduate schools and other scientific positions (12,52–54). Unpaid and underpaid undergraduate positions are prevalent in the environmental sciences due to funding limitations and outdated attitudes among researchers that unpaid work is a rite of passage (53,55); this practice may exclude people who may have inadequate financial support, different abilities, or responsibilities at home (12,56), which reduces diversity in the field. A nationwide survey of undergraduate students demonstrated that pay below minimum wage was the largest barrier to field and research experiences, followed by incompatible scheduling and non-inclusive work environments (54). Even when paid positions are available, they are extremely competitive and often require previous experience; the ability to take an unpaid position may reflect a person's connections within the field and has been associated with a greater likelihood of persisting in STEM (53). In our survey, scientists from marginalized groups may have seen a lack of funded undergraduate research opportunities as a barrier to their own career paths or to their attempts to hire research assistants from diverse groups.Scientists from non-marginalized backgrounds may see unpaid internships as a rite of passage based on their own experiences and may fail to consider their negative impacts (53,55). In response to these survey results, NE CASC has begun participating in a USGS-funded paid summer research program for undergraduates called

Climate Adaptation Scientists of Tomorrow and to date has hosted nine undergraduate researchers at UMass. Increased funding for undergraduate students may support access to research opportunities and field experiences for students facing systemic financial barriers.

Sustainable university DEI programs require faculty involvement and sustained funding, as graduate students are only transiently involved. Inclusion of DEI service work in job responsibilities along with recognition and reward structures for participation in DEI service work may help spur other individuals to get involved. In our study, people from marginalized backgrounds called for more funding for DEI work while also noting concerns with the sustainability of DEI work that they perceived to be led primarily by graduate students of color. Unequal participation in service work is a known issue in academia; in a study of 140 institutions, being a female faculty member was associated with performing significantly more service work across academic rank, race/ethnicity, and field (42). Within STEM departments, Perez et al. (43) found that racially minoritized (Black, Latino/Hispanic, Native American, Asian American, Multiracial) women graduate students were “compelled to initiate change and engage in labor to advance diversity, equity, and inclusion because they could not wait on faculty to act”, and that this investment “negatively affected their well-being and academic success, limited their access to professional opportunities, and unintentionally, perpetuated faculty inaction” (43). Graduate students in these roles also noted unacknowledged emotional labor, like addressing microaggressions and mentoring other students. As a result, racially minoritized women find themselves in a no-win situation: if they do not act they must work in a toxic environment, and if they do act they are chastised for spending too much time on DEI initiatives or their accomplishments are co-opted by faculty (43).

It is important to note that while this study references the broad category of “identifies as marginalized”, and the singular categories of gender, age, and career stage (fellow vs. non-fellow), the role of intersectionality in the experiences of survey participants is a crucial one that could not be quantitatively examined while maintaining survey confidentiality. Despite lacking a method for quantitative evaluation, we emphasize the potential role of intersectionality in the responses of this survey. Interpreting the results from a solely gender or racial viewpoint would potentially exclude the multidimensional experiences of identities of both racial and gender minorities (20). Furthermore, gender analysis has historically centered the experiences of white women, as identified by Crenshaw (20) as the “implicit grounding of white female experiences.” We acknowledge in this study that “gender” as a category may disproportionately center the experiences of white women, while “identify as marginalized” as a category may in a similar way center the experiences of one minority group over those holding intersectional identities that may be “multiply-burdened” (20). Therefore, while we continue to discuss “gender” and “identify as marginalized” categories from the survey, we consider the results of the survey with intersectionality in mind, so as to not discuss issues from singularly gender or racial frameworks that would overlook multidimensional challenges faced by intersectional identities.

Conclusions

By allowing individuals to self-identify as historically marginalized in the fields of climate adaptation, ecology, and environmental science, and by centering our analyses on these individuals, we were able to address intersectionality of experiences and highlight voices of historically marginalized members of the NE CASC community and identify some priorities for action. This approach may be especially useful in smaller organizations. While grouping

"marginalized" people into one group can have the disadvantage of generalizing and assuming a monolithic experience, in cases of small or organizations with very low representation, we argue that using this tool can help to center voices of marginalized people without identifying individuals. This said, our marginalized category prevents closer examination of specific experiences of people from marginalized backgrounds who will have unique experiences: for example, a person with a physical disability will face different barriers to inclusion than a BIPOC person. Similarly, this category is limited because we do not know if everyone who is traditionally grouped as "marginalized or underrepresented" based on any demographic factor, also identified as "marginalized or underrepresented" in the survey. Thus, while this term can support intersectional analysis, it does limit interpretation. Further, when demographic data are used to isolate "marginalized" individuals, institutions become responsible for deciding who is heard and who is not. Allowing survey participants to self-identify as coming from a historically marginalized group allows an assessment of how respondents from these backgrounds feel without displaying identifiable data or making assumptions about background and experience. We used this method to highlight the voices and needs of the NE CASC community, which allowed for more targeted action steps to support DEI goals. Our methods may be useful for other small organizations who want to complete similar climate studies.

Informed by this survey, NE CASC leadership have committed to long-term action in support of DEI. These actions include continuing to fund graduate DEI fellows to focus on building opportunities for peer-mentoring and leading discussion groups of DEI-related research, adding funds for a travel grant to subsidize conference travel for fellows from historically marginalized groups, continuing to survey the NE CASC community to understand changing priorities on DEI, hiring a consultant to evaluate and suggest strategies to improve how PIs

mentor graduate students and postdocs, and hosting undergraduate research students from historically marginalized groups through the USGS' 'Climate Adaptation Scientists of Tomorrow' program. While NE CASC represents only a portion of most students' experience in science, the center aims to increase support and sense of belonging in the hopes of creating a similarly positive experience for all community members.

Acknowledgements

All opinions expressed in this paper are the authors and do not necessarily reflect the policies and views of DOI or USGS. Any use of trade, firm or product names is for descriptive purposes only and does not imply endorsement by the U.S. Government. We are grateful to the NECASC consortium for supporting this work and participating in our survey. We would also like to thank the staff at the University of Massachusetts Office of Equity and Inclusion inspiring our use of a survey and reviewing initial plans for survey execution.

Author Contributions

Meghna N. Marjadi contributed to conceptualization, survey development, analysis, figure creation, writing, and editing. Rebecca A. Smith contributed to analysis, figure creation, writing, and editing. Hsin Fei Tu contributed to survey data organization, analysis, writing, and editing. Asha M. Ajmani contributed to survey development, writing, and editing. Addie Rose Holland contributed to survey development, writing, and editing. Bianca E. Lopez contributed to survey development, writing, and editing. Toni Lyn Morelli contributed to survey development, writing, and editing. Bethany A. Bradley contributed to conceptualization, survey development, analysis, figures, writing, and editing.

Conflict of Interest Statement:

This was a community based research project. All the authors have or are members of the NECASC community and have been either directly or indirectly funded by the center to complete this study or other research. The authors do not declare any other conflicts of interest related to this publication.

References

1. Beck C, Boersma K, Tysor CS, Middendorf G. Diversity at 100: women and underrepresented minorities in the ESA. Front Ecol Environ. 2014;12(8):434–6.https://tos.org/oceanography/article/unpaid-internships-are-a-barrier-to-diverse-and-equitable-recruitment-in-marine-science
2. Bowser G, Cid CR. Developing the ecological scientist mindset among underrepresented students in ecology fields. Ecol Appl. 2021;31(6):e02348.
3. Cech EA. The intersectional privilege of white able-bodied heterosexual men in STEM. Sci Adv. 2022 Jun 15;8(24):eabo1558.
4. Kou-Giesbrecht S. Asian Americans: The Forgotten Minority in Ecology. Bull Ecol Soc Am. 2020;101(3):1–5.
5. Schusler TM, Espedido CB, Rivera BK, Hernández M, Howerton AM, Sepp K, et al. Students of colour views on racial equity in environmental sustainability. Nat Sustain. 2021;
6. Barrile GM, Bernard RF, Wilcox RC, Becker JA, Dillon ME, Thomas-Kuzilik RR, et al. Equity, community, and accountability: Leveraging a department-level climate survey as a tool for action. PLOS ONE. 2023 Aug 17;18(8):e0290065.
7. Cantor D, Fisher B, Chibnall S, Townsend R, Lee H, Bruce C, et al. Report on the AAU Campus Climate Survey on Sexual Assault and Sexual Misconduct. Rockville, Maryland: The Association of American Universities, Westat; 2019 p. 439.
8. Ruf J. Why Environmental Studies is Among the Least Diverse Fields in STEM. Diverse: Issues in Higher Education. 2020 Feb 6;36(25):16–8.
9. Taylor DE. The State of Diversity in Environmental Organizations: Mainstream NGOs, Foundations and Government Agencies [Internet]. Green 2.0; 2014. Available from:

www.diversegreen.org/report

10. Díaz S, Settele J, Brondízio ES, Ngo HT, Agard J, Arneth A, et al. Pervasive human-driven decline of life on Earth points to the need for transformative change. Science. 2019;366(1327).

11. Otto IM, Reckien D, Reyer CPO, Marcus R, Le Masson V, Jones L, et al. Social vulnerability to climate change: a review of concepts and evidence. Reg Environ Change. 2017 Aug 1;17(6):1651–62.

12. Bernard RE, Cooperdock EHG. No progress on diversity in 40 years. Nat Geosci. 2018 May;11(5):292–5.

13. Freeman J. LGBTQ scientists are still left out. Nature. 2018 Jul;559(7712):27–8.

14. Cech EA. LGBT Professionals' Workplace Experiences in STEM-Related Federal Agencies. In 2015 [cited 2023 Sep 22]. p. 26.1094.1-26.1094.10. Available from: https://peer.asee.org/lgbt-professionals-workplace-experiences-in-stem-related-federal-agencies

15. Cech EA, Pham MV. Queer in STEM Organizations: Workplace Disadvantages for LGBT Employees in STEM Related Federal Agencies. Soc Sci. 2017 Mar;6(1):12.

16. Landivar LC. Disparities in STEM Employment by Sex, Race, and Hispanic Origin [Internet]. Washington, D.C.: U.S. Census Bureau; 2013 [cited 2023 Sep 25]. Report No.: ACS-24. Available from: https://www.census.gov/library/publications/2013/acs/acs-24.html

17. Taylor DE. Enhancing racial diversity in the Association for Environmental Studies and Sciences. J Environ Stud Sci. 2018 Dec 1;8(4):379–84.

18. Cech EA, Waidzunas TJ. Systemic inequalities for LGBTQ professionals in STEM. Sci Adv. 2021 Jan 15;7(3):eabe0933.

19. Directorate-General for Research and Innovation (European Commission). She figures 2021: tracking progress on the path towards gender equality in research and innovation [Internet]. LU: Publications Office of the European Union; 2021 [cited 2023 Sep 22]. Available from: https://data.europa.eu/doi/10.2777/602295
20. Crenshaw K. Demarginalizing the Intersection of Race and Sex: A Black Feminist Critique of Antidiscrimination Doctrine, Feminist Theory and Antiracist Politics. Univ Chic Leg Forum. 1989;1989(1):139–67.
21. Taylor DE. Race, gender, and faculty diversity in environmental disciplines. In: E. Taylor D, editor. Environment and Social Justice: An International Perspective [Internet]. Emerald Group Publishing Limited; 2010 [cited 2023 Sep 25]. p. 385–407. (Research in Social Problems and Public Policy; vol. 18). Available from: https://doi.org/10.1108/S0196-1152(2010)0000018015
22. Kellert SR. American Attitudes Toward and Knowledge of Animals: An Update. In: Fox MW, Mickley LD, editors. Advances in Animal Welfare Science 1984 [Internet]. Dordrecht: Springer Netherlands; 1985 [cited 2023 Nov 17]. p. 177–213. Available from: http://link.springer.com/10.1007/978-94-009-4998-0_11
23. Stamps SM, Stamps MB. Race, Class and Leisure Activities of Urban Residents. J Leis Res. 1985 Jan 1;17(1):40–56.
24. Miriti MN, Rawson AJ, Mansfield B. The history of natural history and race: Decolonizing human dimensions of ecology. Ecol Appl. 2023;33(1):e2748.
25. Mohai P. Dispelling Old Myths: African American. Environ Sci Policy Sustain Dev. 2003 Jun 1;45(5):10–26.
26. Taylor DE. Racial and Ethnic Differences in Connectedness to Nature and Landscape

Preferences Among College Students. Environ Justice. 2018 Jun;11(3):118–36.

27. Taylor DE. Racial and ethnic differences in the students' readiness, identity, perceptions of institutional diversity, and desire to join the environmental workforce. J Environ Stud Sci. 2018 Jun 1;8(2):152–68.

28. Marín-Spiotta E, Barnes RT, Berhe AA, Hastings MG, Mattheis A, Schneider B, et al. Hostile climates are barriers to diversifying the geosciences. Adv Geosci. 2020 Jul 28;53:117–27.

29. Chaudhury A, Colla S. Next steps in dismantling discrimination: Lessons from ecology and conservation science. Conserv Lett. 2021 Mar;14(2):e12774.

30. Leath S, Chavous T. Black Women's Experiences of Campus Racial Climate and Stigma at Predominantly White Institutions: Insights from a Comparative and Within-Group Approach for STEM and Non-STEM Majors. J Negro Educ. 2018;87(2):125–39.

31. McGee EO. Devalued Black and Latino Racial Identities: A By-Product of STEM College Culture? - Ebony O. McGee, 2016. Am Educ Res J. 2016;53(6):16261662.

32. Rainey K, Dancy M, Mickelson R, Stearns E, Moller S. Race and gender differences in how sense of belonging influences decisions to major in STEM. Int J STEM Educ 2018 51. 2018 Apr;5(1):1–14.

33. Spoon K, LaBerge N, Wapman KH, Zhang S, Morgan AC, Galesic M, et al. Gender and retention patterns among U.S. faculty. Sci Adv. 2023 Oct 20;9(42):eadi2205.

34. Krause KH, Woofter R, Haardörfer R, Windle M, Sales JM, Yount KM. Measuring campus sexual assault and culture: A systematic review of campus climate surveys. Psychol Violence. 2019;9(6):611–22.

35. Pritchard AJ, DeKeseredy WS, Nolan J, Hall-Sanchez A. Who Speaks First? Analyzing

Response Waves in a Large-Scale Campus Climate Survey. J Aggress Maltreatment Trauma. 2019 Aug 9;28(7):888–901.

36. Stout JG, Dasgupta N, Hunsinger M, McManus MA. STEMing the tide: Using ingroup experts to inoculate women's self-concept in science, technology, engineering, and mathematics (STEM). J Pers Soc Psychol. 2011;100(2):255–70.
37. Miller DC, Salkind NJ. Handbook of Research Design and Social Measurement [Internet]. Sage Publications Inc.; 2002 [cited 2023 Sep 25]. Available from: https://us.sagepub.com/en-us/nam/handbook-of-research-design-and-social-measurement/book10296
38. de Winter JCF. Using the Student's t-test with extremely small sample sizes. Pract Assess Res Eval [Internet]. 2019 Nov 25;18(1). Available from: https://scholarworks.umass.edu/pare/vol18/iss1/10
39. R Core Team. R: A language and environment for statistical computing. [Internet]. Vienna, Austria: R Foundation for Statistical Computing; 2023. Available from: https://www.R-project.org/
40. Wickham H. ggplot2: Elegant Graphics for Data Analysis. [Internet]. Springer-Verlag. New York.; 2016. Available from: https://ggplot2.tidyverse.org.
41. Charmaz K. Constructing grounded theory. London ; Thousand Oaks, Calif: Sage Publications; 2006. 208 p.
42. Guarino CM, Borden VMH. Faculty Service Loads and Gender: Are Women Taking Care of the Academic Family? Res High Educ. 2017 Sep 1;58(6):672–94.
43. Perez RJ, Motshubi R, Rodriguez SL. "We are a huge source of labor": Exploring STEM Graduate Students' Roles in Changing Departmental Climate. Rev High Educ. 2022;46(1):33–66.

44. Chaudhary VB, Berhe AA. Ten simple rules for building an antiracist lab. PLOS Comput Biol. 2020 Oct;16(10):e1008210.

45. Golden N, Devarajan K, Balantic C, Drake J, Hallworth MT, Morelli TL. Ten simple rules for productive lab meetings. PLOS Comput Biol. 2021 May 27;17(5):e1008953.

46. Beasley MA. Beyond Diversity: A roadmap to building an inclusive organization [Internet]. Diverse Green; 2017. Available from: https://diversegreen.org/wp-content/uploads/2021/01/BeyondDiversity_Report_060517-1.pdf

47. Yosso T, Smith W, Ceja M, Solórzano D. Critical Race Theory, Racial Microaggressions, and Campus Racial Climate for Latina/o Undergraduates. Harv Educ Rev. 2010 Jan 29;79(4):659–91.

48. Anadu J, Ali H, Jackson C. Ten Steps to Protect BIPOC Scholars in the Field. Eos [Internet]. 2020 Oct 14 [cited 2023 Nov 17];101. Available from: https://eos.org/opinions/ten-steps-to-protect-bipoc-scholars-in-the-field

49. Wolfe BA, Riggs EM. Macrosystem Analysis of Programs and Strategies to Increase Underrepresented Populations in the Geosciences. J Geosci Educ. 2017 Nov;65(4):577–93.

50. Wu DJ, Thiem KC, Dasgupta N. Female peer mentors early in college have lasting positive impacts on female engineering students that persist beyond graduation. Nat Commun. 2022 Nov 11;13(1):6837.

51. Pico T. The Darker Side of John Wesley Powell. Scientific American [Internet]. 2019 Sep 9 [cited 2023 Nov 17]; Available from: https://blogs.scientificamerican.com/voices/the-darker-side-of-john-wesley-powell/

52. Beltran RS, Marnocha E, Race A, Croll DA, Dayton GH, Zavaleta ES. Field courses narrow demographic achievement gaps in ecology and evolutionary biology. Ecol Evol. 2020

Jun;10(12):5184–96.
53. Fournier AMV, Holford AJ, Bond AL, Leighton MA. Unpaid work and access to science professions. Rosenbloom JL, editor. PLOS ONE. 2019 Jun 19;14(6):e0217032.
54. Jensen AJ, Bombaci SP, Gigliotti LC, Harris SN, Marneweck CJ, Muthersbaugh MS, et al. Attracting Diverse Students to Field Experiences Requires Adequate Pay, Flexibility, and Inclusion. BioScience. 2021 Jul 1;71(7):757–70.
55. Fournier AMV, Bond AL. Volunteer field technicians are bad for wildlife ecology: Volunteer Field Technicians Are Bad for Wildlife Ecology. Wildl Soc Bull. 2015 Dec;39(4):819–21.
56. Kreuser A, Bishop A, Meyer-Gutbrod E. FROM THE TOS JEDI COMMITTEE: Unpaid Internships Are a Barrier to Diverse and Equitable Recruitment in Marine Science. Oceanography [Internet]. 2023 May 25 [cited 2023 Aug 14]; Available from: https://tos.org/oceanography/article/unpaid-internships-are-a-barrier-to-diverse-and-equitable-recruitment-in-marine-science

Supporting Information

S1 File. Survey questions, format, and answers. PDF of Survey questions, formats and answers.

S2 File. Anonymized survey data. Anonymized data from our study figures and analyses for each reported question and figure.

Fig 1

Click here to access/download;Figure;Fig1.jpg

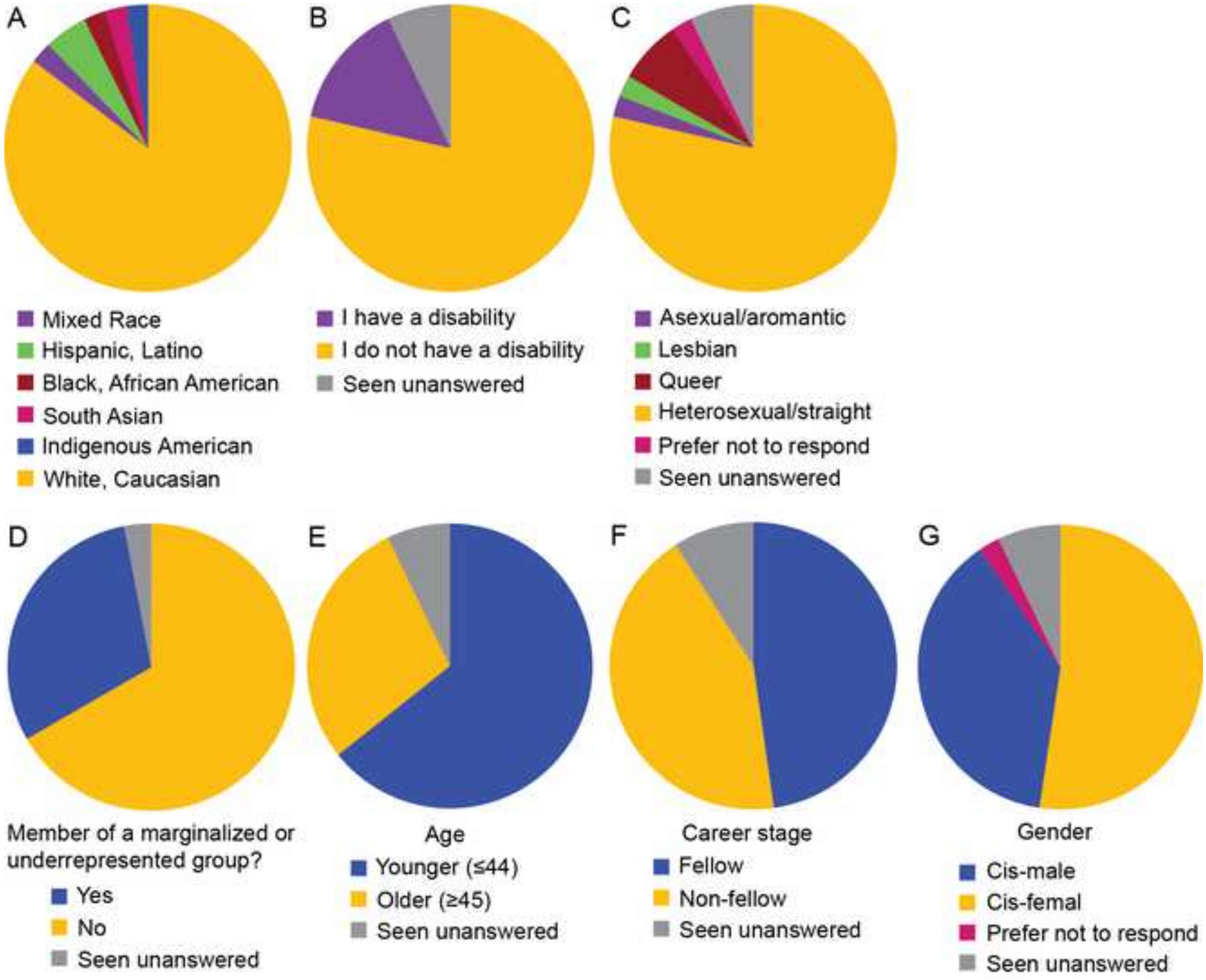

Fig 2

Click here to access/download;Figure;Fig2.tif

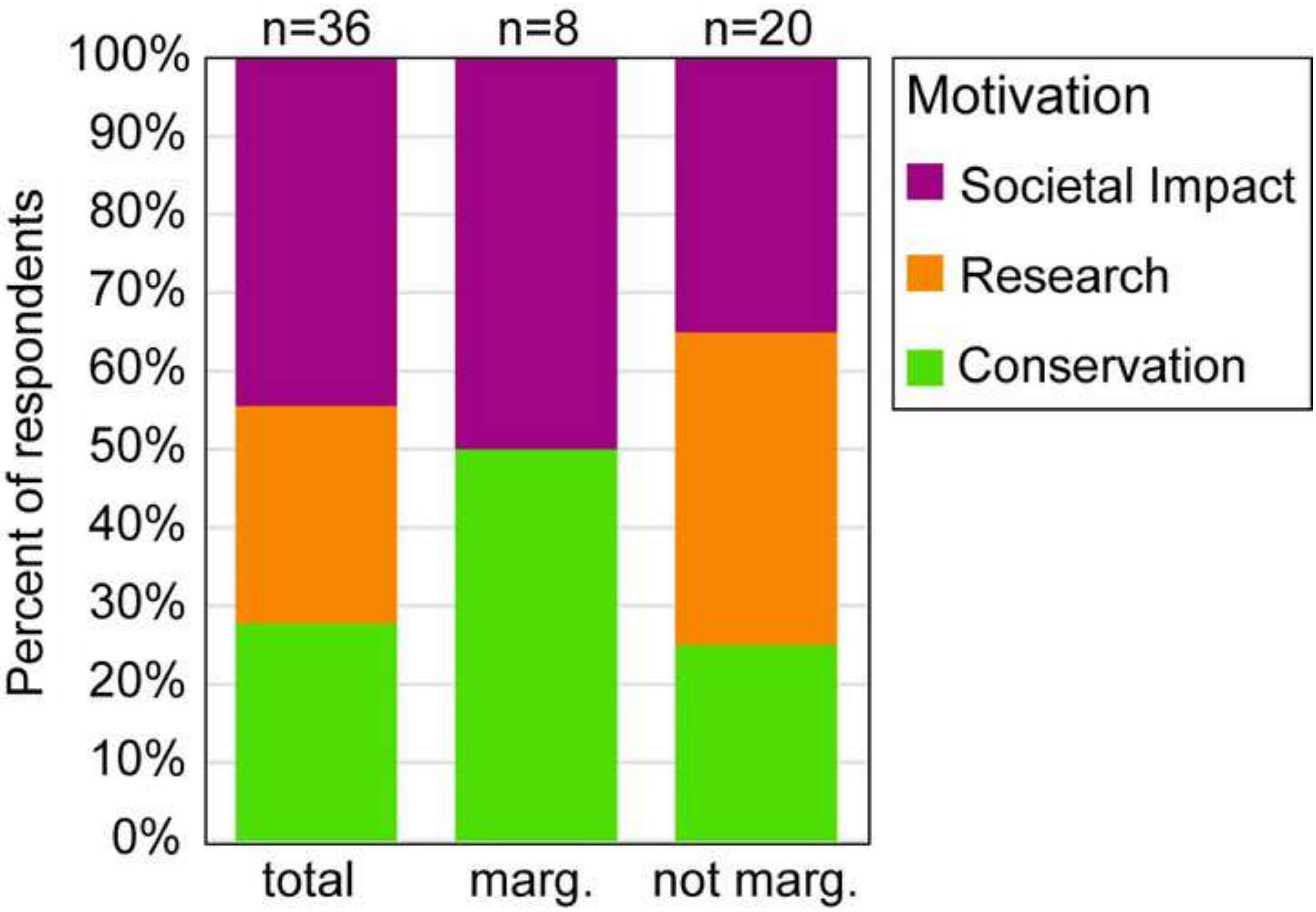

Fig 3 Click here to access/download;Figure;Fig3.tif

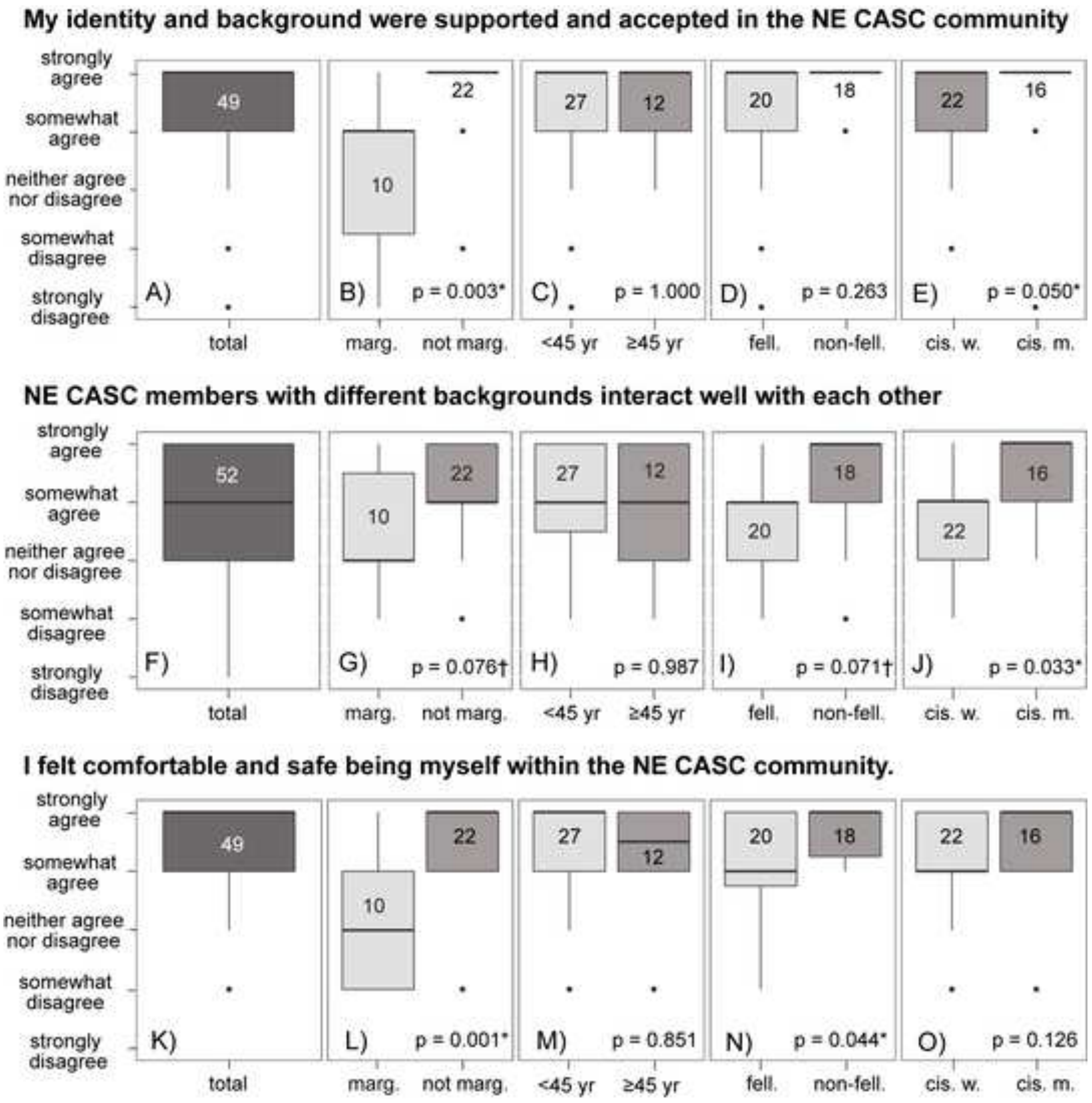

Fig 4

Click here to access/download;Figure;Fig4.tif

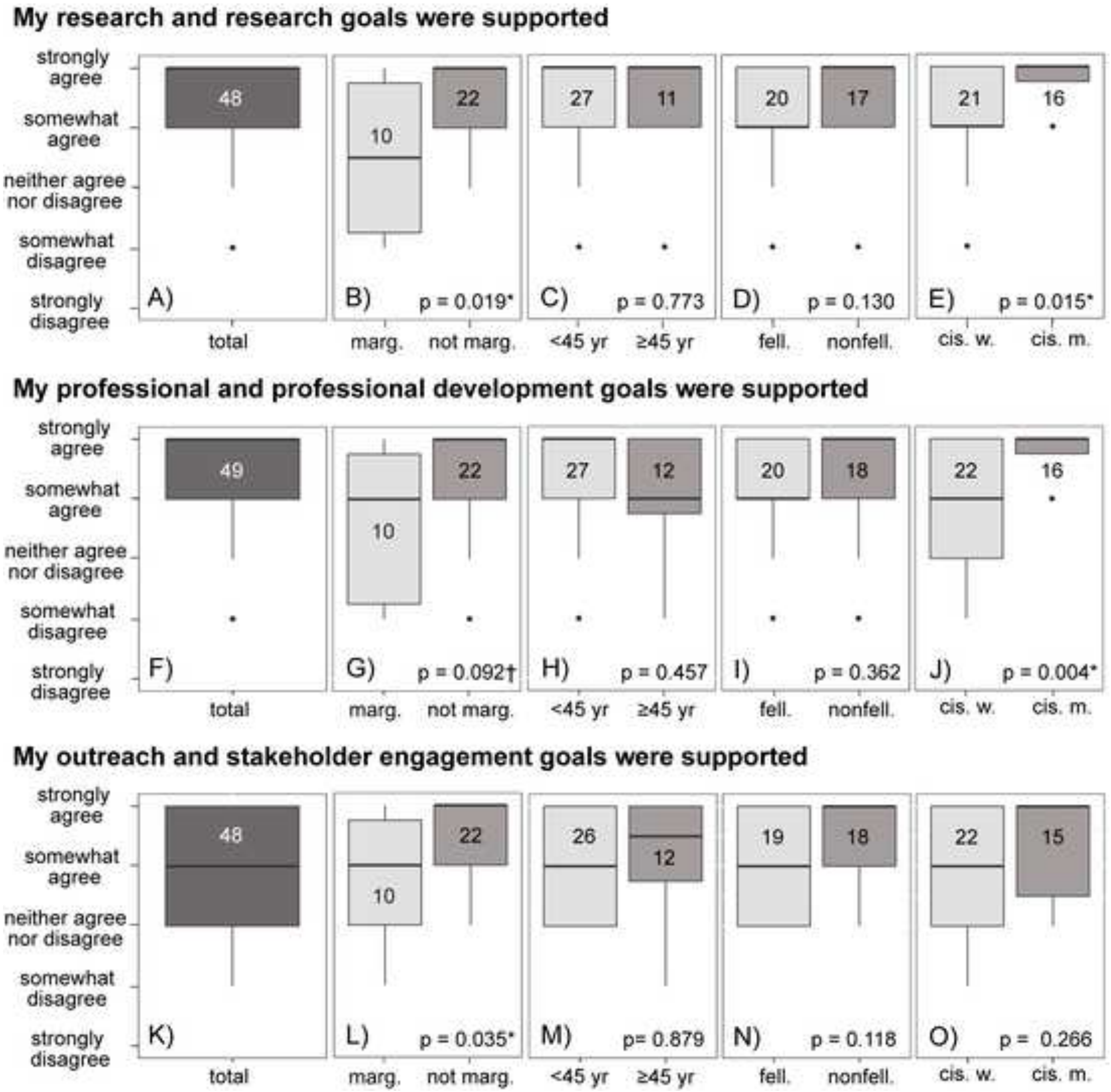

Click here to access/download;Figure;Fig5.tif

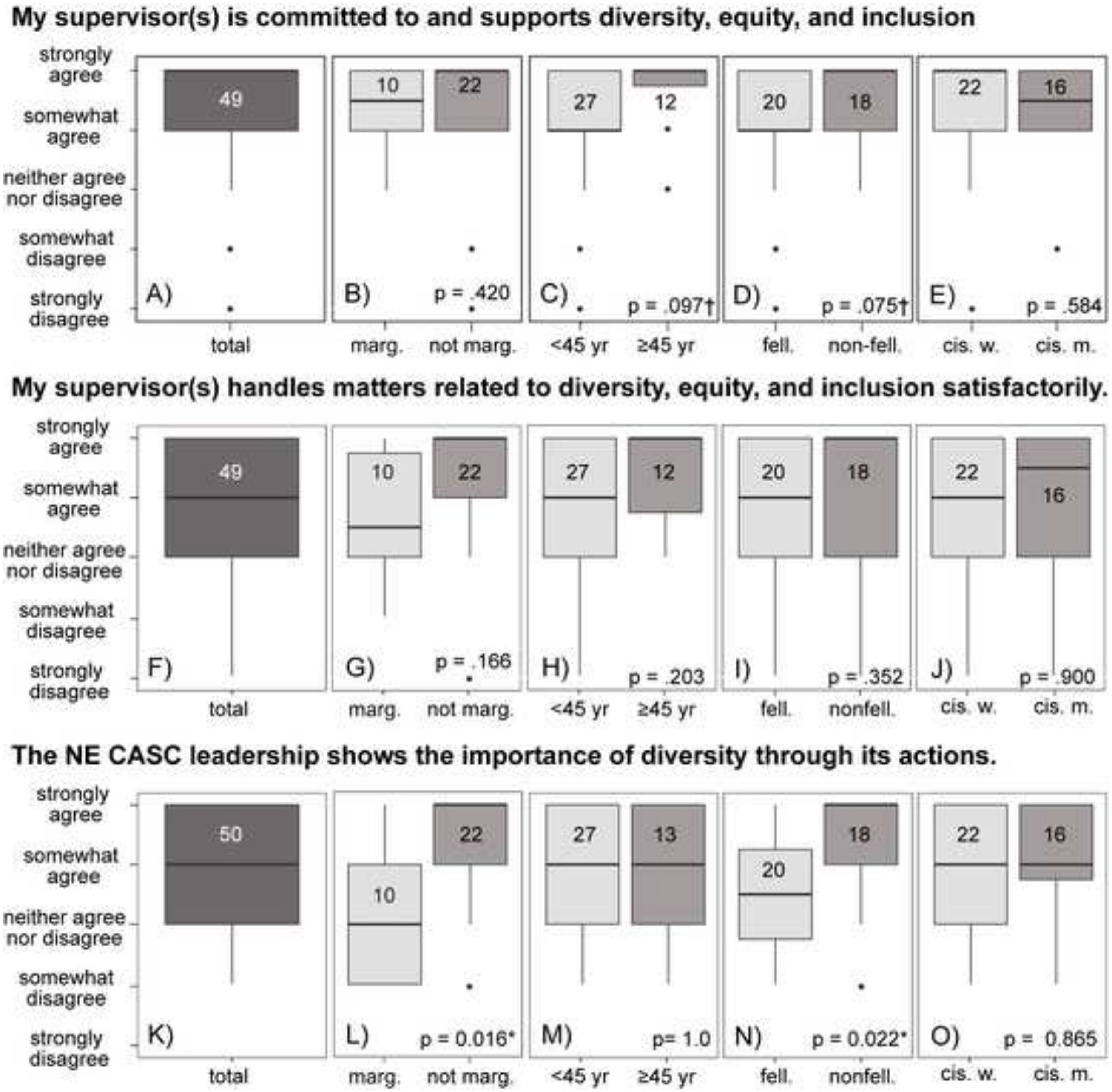

Fig 6

Click here to access/download;Figure;Fig6.tif

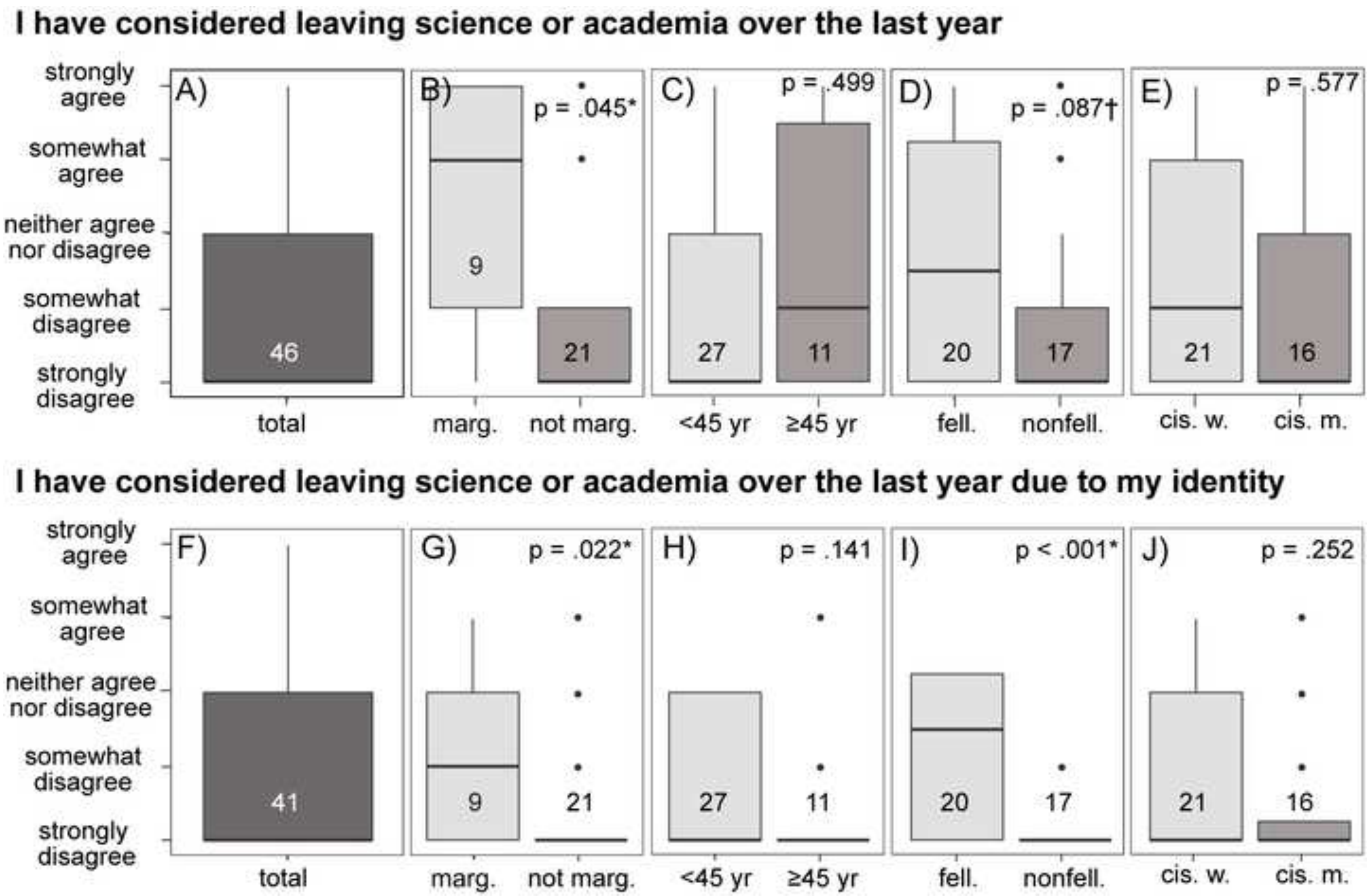

Fig 7

Click here to access/download;Figure;Fig7.tif

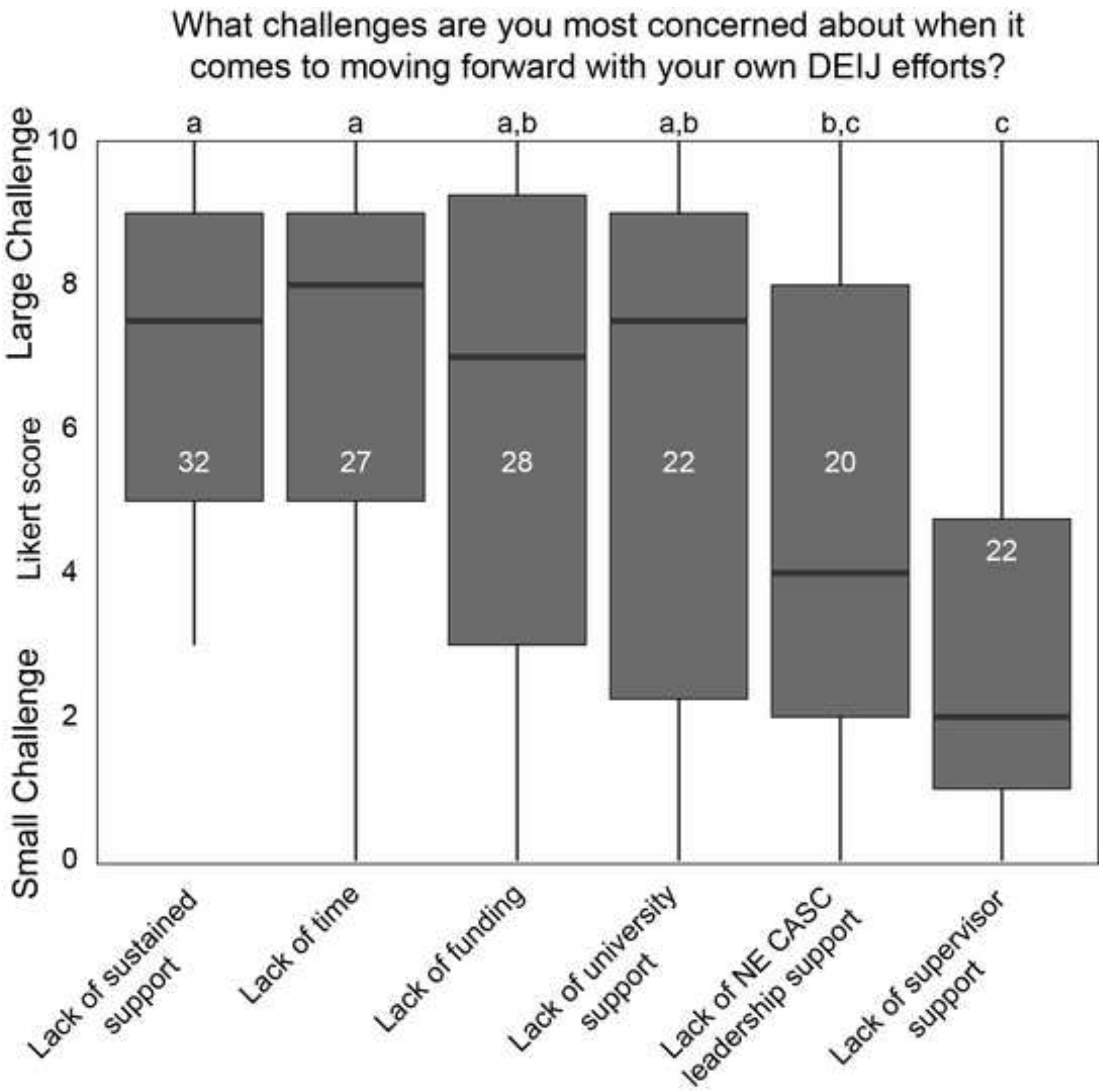

Fig 8

Click here to access/download;Figure;Fig8.tif

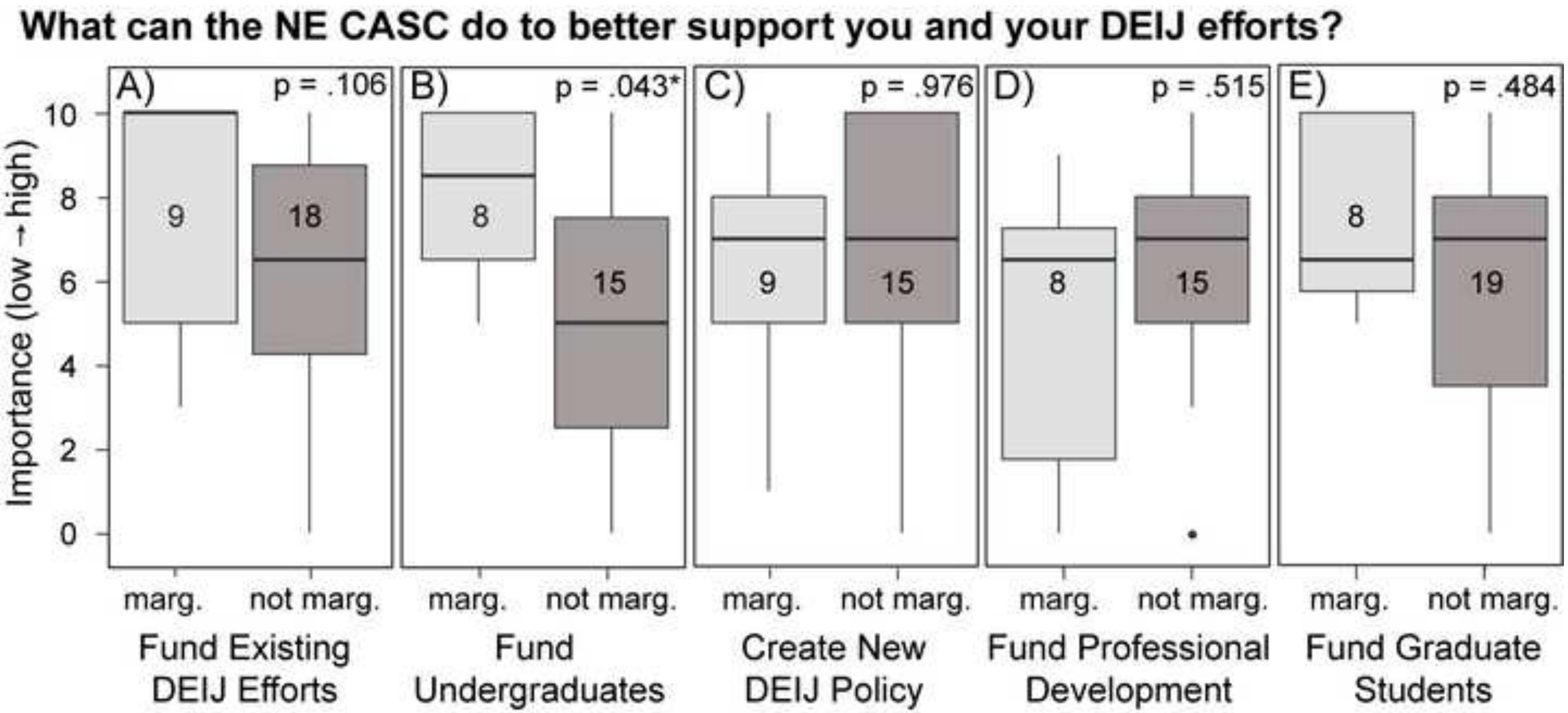

Click here to access/download
Supporting Information
S1_File.pdf

Click here to access/download
Supporting Information
S2_File.pdf

Revised Manuscript with Track Changes

Centering voices of scientists from marginalized backgrounds to understand experiences in climate adaptation science and inform action

Short title: Centering marginalized voices in climate adaptation

Meghna N. Marjadi[1,5,#a*], Rebecca A. Smith[2,5], Hsin Fei Tu[3], Asha M. Ajmani[4,5], Addie Rose Holland[5,8], Bianca E. Lopez[4,5,6], Toni Lyn Morelli[4,51,7], Bethany A. Bradley[4,5]

[1]Graduate Program in Organismic and Evolutionary Biology, University of Massachusetts, Amherst, MA, 01003; Current: School of Marine Sciences and Technology, University of Massachusetts, Dartmouth, 836 South Rodney French Boulevard, New Bedford, MA 02744

[2]Department of Geosciences, University of Massachusetts, Amherst, MA, 01003 Massachusetts, United States of America

[3]Department of Sociology, University of Massachusetts, Amherst, MA, 01003Massachusetts, United States of America

[4]Department of Environmental Conservation, University of Massachusetts, Amherst, MA, 01003Massachusetts, United States of America

[5]Northeast Climate Adaptation Science Center, University of Massachusetts, Amherst, MA, 01003Amherst, Massachusetts, United States of America

[6]American Association for the Advancement of Science, 1200 New York Ave, NW Washington, DC 20005District of Columbia, United States of America

[7]U.S. Geological Survey, Northeast Climate Adaptation Science Center, Amherst, MA, 01003Massachusetts, United States of America

[8]Center for Braiding Indigenous Knowledges and Science, University of Massachusetts, Amherst, Amherst Massachusetts, United States of America

[#a]Current: School of Marine Sciences and Technology, University of Massachusetts, Dartmouth, New Bedford, Massachusetts, United States of America

*Corresponding author:
E-mail: mmarjadi@umassd.edummarjadi@umassd.edu (MNM)

Style Definition: Heading 1
Style Definition: Heading 2
Style Definition: Heading 3
Style Definition: Heading 4
Style Definition: Heading 5
Style Definition: Heading 6
Formatted
Formatted: Superscript
Formatted: Font: Not Bold
Formatted: Default Paragraph Font, Font: Arial, 11 pt, Font color: Black
Formatted: Font color: Black

Keywords: diversity~~,~~; equity~~, and~~; inclusion; DEI; climate ~~surveys; unrepresented~~survey; underrepresented scientists;

Formatted: Font color: Black

Formatted: Font color: Black

Formatted: Font color: Black

Formatted: Font color: Black

Formatted: Font color: Black

Abstract

Identifying and building solutions to help people and ecosystems adapt to climate change requires ~~the voices and efforts~~participation of all people. Unfortunately, ~~science, technology, engineering, and math~~Science, Technology, Engineering, and Mathematics (STEM) fields, including environmental sciences~~ and ecology~~, continue to ~~suffer from a ~~lack ~~of ~~diversity. To address this ~~lack of diversity~~issue, many institutions have increased programming to recruit and retain people from historically marginalized backgrounds into STEM fields. Institutions use surveys to evaluate the experiences of community members and identify areas for improvement; however, surveys ~~in less diverse fields could focus on the~~often summarize and reflect majority ~~perspective,~~perspectives and ~~cause~~disregard voices of historically marginalized individuals~~ to go unheard.~~. In June~~ of~~ 2021, we surveyed graduate students, postdocs, faculty, staff, and researchers affiliated with the Northeast Climate Adaptation Science Center (NE CASC) to evaluate their experiences of diversity, equity, and inclusion (DEI) using Likert-based and long-answer questions. We ~~evaluated~~analyzed the results as a whole, but also focused on the responses of people who self-identified as ~~coming from~~members of a marginalized ~~background~~group (“marginalized respondents”) in climate adaptation science~~, aiming~~ to center ~~these~~their voices~~ to understand experiences in science and identify key areas for improvement.~~. Marginalized respondents reported ~~that they were~~being motivated to enter climate adaptation science to improve society and the environment ~~and not~~rather than for intellectual curiosity, which ~~was a motivator for~~motivated one third of non-marginalized respondents. Once in science ~~fields~~, marginalized respondents reported feeling less supported and comfortable ~~being themselves in their ~~at work~~ environment~~ and were more likely to have considered leaving science

and academia in the last year. Long-answer responses of marginalized respondents indicated distrust in the ability of leadership and existing DEI ~~efforts~~initiatives to ~~address issues of~~effectively tackle systemic ~~racism,~~issues and emphasized the importance of focusing on equity and inclusion before recruitment. Marginalized respondents identified additional funding ~~for~~to support existing DEI efforts and ~~funding for~~ undergraduates as ~~top~~ priorities~~ for further action - both of which have been prioritized by NE CASC.~~. By allowing participants to self-identify as part of a marginalized group, we were able to highlight experiences and needs without ~~risk of identifying individuals~~risking exposure based on race, gender, disability, or sexual orientation. This approach can be applied to other small organizations with limited demographic diversity.

Introduction

~~Ecology and~~The environmental sciences suffer from a diversity problem, with lower proportions of Black, Indigenous, people of color (BIPOC), lesbian, gay, bisexual, transgender, or queer (LGBTQ+) people, people living with disabilities, and women relative to the general population (1~~–3~~4). Retaining scholars from groups underrepresented in ~~ecology and~~ environmental ~~science~~sciences is a key aspect of addressing this lack of diversity (~~4,~~5) and many institutions have created surveys to understand how they can improve conditions and create a more equitable and inclusive workplace (6,7). Centering the voices of scientists from historically marginalized groups enables action that directly addresses barriers to an equitable and inclusive workplace. However, since people from historically marginalized groups comprise a small minority of the workplace for most ~~ecology,~~ environmental ~~sciences, and geoscience~~science fields (8,9), their needs may be overshadowed by majority opinion in averaged survey responses.

Environmental ~~and ecological~~ challenges are multifaceted and solutions require collaboration across disciplines and ~~background (4~~backgrounds (10). The need for diverse perspectives and collaborations is underscored by evidence that ~~predicted~~ climate ~~changes have~~change has already and will continue to have the most detrimental effects ~~for~~on people from historically marginalized groups, who ~~may not be adequately represented in~~are often excluded from scientific and policy discussions (~~10). Ecology, environmental sciences~~11). Environmental science fields, which include ecology, hydrology, climate science, and geosciences, are among the least diverse disciplines in Science, Technology, Engineering, and Mathematics (~~9,11~~8,12), lacking both racial and LGBTQ+ diversity in both academic (1,~~3,12~~4,9,13) and non-academic

contexts (~~8~~9,14,15). For example, although racial and ethnic minorities comprised 29% of the US STEM workforce and 38% of the US population in 2018 (16), only 10.5% of the workforce in environmental science and conservation fields identified as members of a racial or ethnic minority group (5% Black, 0.7% Asian, 4.8% ~~Latinx) (13).~~Latino/Hispanic) (17). Similarly, only 16% of staff in US environmental agencies, foundations, and nonprofits identified as non-white ~~minorities (8~~(9). While data are lacking for environmental fields specifically, LGBTQ+ people ~~are~~have been noted as underrepresented in STEM fields by an estimated 17-21% (~~12~~13) and commonly face negative workplace experiences as STEM professionals (15,~~17~~18).

Other demographics, like gender, national origin, religion, disability status, and financial background, also likely influence people's sense of belonging and long-term participation in STEM and environmental science fields. For example, cisgender women receive similar numbers of STEM PhDs as cisgender men, but represent only 24% of leadership positions in higher education (~~18~~19). Similarly, while women comprise 60% of new hires in environmental organizations, men dominate top leadership positions across environmental organizations (~~8~~9). Perhaps not surprisingly given their majority status, white, able-bodied, heterosexual men report feeling more included and respected, and have more career opportunities in STEM than people from all other racial, disability status, sexual orientation, and gender groups (14).

Further, scientific and academic spaces often fail to center intersectional identities; for example, a queer person of color with a disability may face discrimination that a straight or able-bodied person of color does not (~~19~~20). Disparities are often heightened for women of color and other individuals who sit at the intersection of identities. For example, from -1973 to 2016, Native American, Black, Hispanic or Latino women together received only 1.46% of PhDs in geosciences (~~11~~12). Similarly, Asian men hold twice as many faculty positions and three times

as many tenured faculty positions as Asian women, resulting in the largest gender disparity across all racial groups in academic environmental disciplines (~~3,20~~4,21).

Lack of diverse representation in ~~ecology and~~ environmental sciences is not caused by lack of interest~~.~~, but instead a lack of opportunity and support. Outdated and spurious claims that ~~minority~~underrepresented students, especially BIPOC students, are disinterested ~~and disconnected with~~in nature ~~ecology~~ and the environmental sciences (~~21,~~22~~)~~ ,23) are baseless and have been disproven in multiple studies (~~23–25). Further, high percentages of students of color report interest in working with federal environmental agencies and nonprofits; in~~24–26). In one study, 91.2% of students from racial and ethnic minority groups reported feeling somewhat or very connected to nature (~~25).~~26). In fact, high percentages of BIPOC students report interest in working with federal environmental agencies and nonprofits (Taylor, 2018b). Although minority enrollment~~,~~ and performance~~, and aptitude~~ in STEM fields are comparable to those of white students, a significant percentage of Asian, Black, ~~Latinx~~Latino/Hispanic, and Native American students do not complete STEM degrees (~~26). These~~17,27). In the environmental sciences, historical legacies of exclusion, hierarchical power dynamics, and/or unsafe fieldwork or learning conditions may contribute to students leaving STEM programs (28). Additionally, retention issues may reflect systemic failures of university environments and ~~environmental science~~ curricula to meet the needs of diverse ~~students~~ student interests (5,~~27~~29). Even when they do attain STEM degrees, students from racial and other minority groups face additional barriers as they pursue advanced degrees and move forward in their careers (28). ~~Lack of diversity in environmental fields continues due in part to historical legacies of exclusion, hierarchical power dynamics, and unsafe fieldwork conditions (28).~~

Attrition in the environmental science~~, ecology, and geoscience~~ fields may be related to the many barriers to equity and inclusion, which include bias, discrimination, and harassment in both educational environments and the workplace (28). Within ecology and environmental sciences, students of color have reported discrimination, lack of relatability, and limited discussions of race as promoting feelings of isolation and exclusion in the environmental sciences (5). ~~Additionally,~~More broadly, Black and ~~Latinx~~Latino/Hispanic STEM undergraduate students experience microaggressions and other racist incidents that can cause lasting psychological impacts and reduce productivity, dampen interest, and ultimately cause students to change fields (~~29,~~30~~). Similarly,~~,31), Cantor et al. (~~2019~~7) reported that 59% of female undergraduate students in STEM fields in the U.S. have experienced harassment. Retention of members of historically marginalized communities in science begins with identifying and dismantling barriers at early career stages~~.~~ (32,33).

~~In order to identify and address barriers to retention of scientists from historically marginalized backgrounds, we~~Feeling a sense of belonging – defined as “the feeling of security and support when there is a sense of acceptance, inclusion, and identity for a member of a certain group (6)” – has been associated with increased retention for students in STEM majors (32) and faculty across academic fields (33). For undergraduate students, representation within STEM subdisciplines, interpersonal relationships, perceived competence, and science identity contributed to feelings of belonging and the desire to stay in their majors (32). Similarly, a census study revealed that women left academic positions at higher rates than men and were more likely than men to leave positions because they felt “pushed out” or unsupported in their role, compared to men who were more likely to feel “pulled” to a better opportunity (33).

Formatted: Highlight

University campuses have employed institution-wide “climate surveys” to collect demographic information and assess workplace dynamics (34). Since surveys are often deployed across the entire institution (34,35), they do not account for department-specific dynamics (6). Similarly, many studies on belonging and retention in STEM have been conducted across disparate STEM fields (30,31,36) and may not reflect the unique experiences of researchers in environmental sciences, which includes some of the least diverse STEM fields (4,8,12). Many articles on diversity in the environmental sciences have used compilations of existing data to answer questions about demographic trends (1,2,9,12) but do not use survey or interview tools to investigate the underlying reasons for those trends. Although a few studies have used interviews and surveys to address belonging and retention for undergraduate students in ecology and environmental sciences (5,26,27), no studies have been published in the peer-reviewed literature recently that evaluated graduate student, postdoctoral fellow, or faculty experiences at a departmental or programmatic level based on marginalized or underrepresented identity. Barrile et al. (6) used a survey to evaluate experiences in a zoology department and considered differences in experiences across career levels, but did not analyze demographic characteristics. Evaluating departmental, programmatic, and field specific dynamics for researchers at different career stages may prove valuable in better understanding experiences of scientists from diverse backgrounds in environmental sciences.

We surveyed students, postdoctoral scholars, faculty, staff, and other researchers affiliated with the Northeast Climate Adaptation Science Center (NE CASC) to better understand their experiences in the ~~experiences~~program. Ultimately, we planned to use survey results to identify and address barriers to retention of ~~all members. While~~scientists from historically

marginalized backgrounds in the NECASC and in environmental science fields. The NECASC includes scientists in environmental sciences–including ecologists, hydrologists, social scientists, and geoscientists–with a focus on climate adaptation science and climate science research. Other DEI related studies in environmental science and ecology have been focused on undergraduate students (5,26,27,32) or professionals outside of academia (9).

As a cross-institutional network, the NE CASC represents only a small portion of most respondents' experiences in science. Academic departments, colleges, universities may play a more prominent role in student, postdoctoral scholar, and faculty experiences. Nevertheless, actions of the center to improve equity and inclusion could support retention of community members from historically marginalized groups. We collected responses to a series of Likert scale and open-ended survey questions and listened to individual stories through workshops. This study aims to center and amplify the voices of individuals from historically marginalized backgrounds and focuses on questions related to 1) the motivation of people affiliated with the NE CASC (hereafter, NE CASC ~~community~~consortium members) for entering careers related to climate adaptation~~ science fields~~, 2) how supported community members feel as individuals and in their work, including their likelihood of leaving scientific research, and 3) barriers to and priorities for DEI action.

Methods

Case study site and community

Formatted: Font color: Black

The Climate Adaptation Science Centers (CASCs) are a partnership between the U.S. Geological Survey (USGS) and consortium institutions (including universities, Tribal

organizations, government agencies, and non-governmental organizations) to develop science to help fish, wildlife, water, land, and people adapt to a changing climate. The NE CASC is hosted by the University of Massachusetts Amherst. As of June 2021, when this survey was conducted, the NE CASC consortium institutions included the University of Vermont, Cornell University, Woodwell Climate Research Center, USFS Northern Research Station, Columbia University, the College of Menominee Nation, University of Wisconsin, University of Missouri, and Michigan State University. The NE CASC community includes graduate students (both M.S. and Ph.D.), postdoctoral scholars, university faculty, tribal organization staff, federal and university administrative staff, and research scientists. Between its inception in 2012 and when this survey was conducted in 2021, a total of 177 individuals have been associated with the NE CASC ~~community~~consortium (115 graduate students and postdoctoral scholars, collectively referred to as fellows, 43 funded principal investigators, and 19 staff). Graduate students and postdoctoral fellows join the NE CASC Fellows program when they are funded on a NE CASC project. Undergraduate students may support NE CASC projects, but few have formal involvement with the NE CASC. We grouped both currently and formerly affiliated community members to include voices spanning the NE CASC's existence. To guide work on retaining members of the community and making activities more equitable and inclusive, the NE CASC sought to understand challenges faced by their community members, especially those from underrepresented groups, and to identify priority areas in need of support.

Community~~-~~-based participatory research process and survey development

Beginning in the fall semester of 2020, the NE CASC has supported new DEI initiatives by funding graduate student 'DEI Fellows', some of whom focused on understanding and improving the NE CASC's recruitment and retention of students and staff from underrepresented groups. ~~At~~Until that time, the NE CASC's DEI organizational goals and activities related largely to training early career scientists in ethical engagement principles, building engagement with regional Tribal Nations, prioritizing climate adaptation projects relevant to Tribal Nations, ~~training early career scientists in ethical engagement principles~~, and including Native students in the NE CASC Fellows Program in partnership with a Tribal college (the College of Menominee Nation) and through the Bureau of Indian Affairs (BIA) Pathways internships (NE CASC Tribal Nation Partners website, 2013-2018 NE CSC Strategic Science Agenda).

To begin understanding how the NE CASC could support new DEI initiatives, the DEI Fellows ~~held~~led a workshop on December 2, 2020, where community members identified and discussed DEI priorities. At the workshop, 22 members of the NE CASC community discussed the need for a survey to assess existing diversity, identify needs, and prioritize actions. As an outcome of the workshop, a community climate survey was developed and the NE CASC formed a DEI committee to plan and oversee diversity, equity, and inclusion efforts for the NE CASC. Members of the NE CASC community and DEI committee were involved in survey development and implementation.

Formatted: Font color: Black

Formatted: Font color: Black

Survey development and implementation

Formatted: Font color: Black

Our survey was motivated by the outcomes of the December workshop, where it was noted that NE CASC did not have any baseline data of existing diversity among community members, nor information about priority areas for future DEI work.. We created the initial survey with guidance from campus climate surveys that were freely available online, including surveys administered by the University of Massachusetts, Amherst, the University of Michigan, and the University of California, Los Angeles. We tailored our survey to the NE CASC communityconsortium and included specific questions about climate adaptation research and the NE CASC's processes around recruitment, hiring, and mentoring (S1 Table). The NE CASC's leadership and DEI committee reviewed and tested the survey. The survey was implemented in Qualtrics and sent to the e-mail addresses of 177 communityconsortium members who were previously or currently affiliated with the NE CASC (as of June 2021). The survey employed Likert scale, multiple choice, and open answer questions (S1 Table) and was estimated to take about 30 minutes to complete. We began soliciting survey responses on June 4th, 2021 and accepted responses until July, 9th 2021. We sent three reminder emails to the community during that time. The survey was conducted in accordance with the University of Massachusetts Internal Review Board (no. 2705).

Demographic information

Formatted: Font color: Black

Although the survey was anonymous, we collected demographic information that could have made some individuals identifiable. To protect privacy, we worked with the University of Massachusetts Amherst's Institute for Social Science Research (ISSR) to anonymize the dataset and ensure that the NE CASC leadership only viewed the results in aggregate. Due to the small

sample size, we were unable to split the dataset based on race, sexuality, or disability status without compromising the identities of respondents. ~~However, one~~All participants self-identified their demographic information and could answer yes or no to report if they had any sort of disability (either mental or physical). One of our survey questions asked respondents whether they identify as a member of a marginalized community in science (Yes, No). The group responding 'yes' (hereafter, marginalized) was large enough that individuals could not be identified and was used as our primary grouping for comparing their experiences to the experiences of people that responded 'no' (not marginalized).

Additional groupings included age (Younger: <45, Older: ≥45), gender (cisgender man, cisgender woman), and career stage (graduate student or postdoctoral (fellow), later-career faculty or staff (non-fellow)). We only considered self-reported demographic information. Thus, participants could only be grouped if they answered the corresponding demographic survey questions, which were at the end of the survey and were skipped by some respondents. Responses lacking demographic data were included in the analysis of total responses but excluded in group comparisons. As a result, the number of total respondents to the survey and the number of participants included in grouped data differs by question.

Formatted: Font: Gungsuh

Quantitative analysis

Formatted: Font color: Black

Likert-scale values were converted to numeric as follows: strongly disagree = 1, somewhat disagree = 2, neither agree nor disagree = 3, somewhat agree = 4, strongly agree = 5. We applied the non-parametric Mann-Whitney U-test to compare distributions of Likert-scores between groups. The Mann-Whitney U-test is appropriate because the data are not normally distributed, groups are unequally sized, and sample sizes are small ~~(31~~ (37). Due to low

statistical power in small sample sizes, we only report statistical results for questions that had at least 8 responses for each category tested (~~32~~38). Each question was analyzed independently. We interpreted a p-value ≤ 0.05 as a significant difference between groups and a p-value ≤ 0.10 as a somewhat significant difference between groups. Plots were created in R computing language (~~33~~39) using the ggplot2 package (~~34~~40).

Formatted: Font: Gungsuh

Qualitative Analysis

Formatted: Font color: Black

We conducted a qualitative analysis for three of the open-answered questions in the survey, for which we had received adequate numbers of responses. We followed grounded theory methodology to complete our coding (41). Three researchers worked on examining short responses, identifying themes, and assigning representative codes to those themes. We then met as a group to discuss coding approaches, find overlapping themes and codes, and identify which themes and codes best represented the data. We then re-coded each of the responses into the new codes. We completed our coding in Microsoft Excel spreadsheets.

To better understand the motivations of the NE CASC community for entering the field of climate adaptation science, we asked the open ended question: "Why did you choose to pursue climate adaptation research?". We coded responses into categories without demographic information; an associate at the ISSR later added the demographic information to the responses. ~~We compared motivations between (self-identified) marginalized and non-marginalized groups using a Kruskal-Wallis rank sum test (35).~~

To compare motivations between (self-identified) marginalized and non-marginalized groups, we used a Kruskal-Wallis rank sum test to test differences in the numbers of responses in

each category for the two groups (35). The Kruskal-Wallis test was appropriate because our dataset was small and the groups were uneven.

To better understand concerns about DEI actions supported by the NE CASC moving forward, we asked the question: "What is your biggest concern for the NE CASC as it moves forward with DEI initiatives?" To focus on the needs of people who identified as marginalized, we asked: "What can the NE CASC do to support you as a member of a marginalized or underrepresented group?" We did not analyze these qualitative data statistically, but instead used the responses to illustrate the concerns and needs of community members who identified as marginalized.

Results

Demographics of the NE CASC community

Formatted: Font color: Black

We collected 42 responses to the survey (a 24% response rate). Most respondents (28; 67%) were affiliated with NE CASC at the time of the survey, with the remaining (14, 33%) being previously affiliated. Overall, 32 (76%) respondents answered the question of whether they self-identified as a member of a marginalized group; of these, 10 (30%) identified as marginalized and 22 (67%) did not identify as marginalized~~ and~~; the remaining survey respondents did not answer ~~the~~this question (~~3~~10; 24%) (Fig 1). We collected more responses from cisgender women (22; 52%) than cisgender men (16; 38%), with some respondents choosing to skip the question about gender (3; 7%) or selecting that they preferred not to respond (1; 2%). The majority of respondents identified as white (35; 83.2%), with the remaining respondents identifying as Indigenous American (1; 2.4%), South Asian (1; 2.4%), Black (1;

2~~%), Latinx~~.4%), Latino/Hispanic (2; ~~5~~4.8%), and mixed race (1; 2.4%); the remaining respondents (1; 2.4%) did not complete the demographic proportion of the survey. The majority of respondents identified as not having a disability (33; 79%) and as heterosexual (33; 79%) (~~Fig 1).~~Fig 1). As this is the first time the NE CASC has collected self-reported demographic data, we have no other demographic data from the NE CASC to compare our responses to the overall community.

Fig 1: Demographic breakdown of respondents to the survey. The majority of respondents identified as A) white, B) not disabled, and C) heterosexual. D) Ten respondents self-identified as being a member of a marginalized or underrepresented group in climate adaptation science. In addition to considering responses based on marginalized vs. non-marginalized groups, we were also able to compare responses for E) older vs. younger, F) fellows (students and postdocs) vs. non-fellows (PIs and staff), and G) cis-men vs. cis-women (the only two gender categories specified in the demographic responses).

Motivation for pursuing climate adaptation science

Formatted: Font color: Black

Our qualitative analysis of the question of personal motivation to pursue climate adaptation science yielded three major categories of reasoning for pursuing climate adaptation research. 1) “Research Interest or Intellectual Curiosity" included responses that were focused on academic research impacts or topical interest and had no connections to specific social or conservation implications (e.g., “It is fundamental to my research interests”; “It was a natural progression of my education and interests”). 2) “Environmental Conservation and Management” included responses that focused on concerns for the future of the planet, conserving resources, and preserving biodiversity (e.g., “I believe that climate change is one of the biggest threats to

non-human species in millions of years"; "I am passionate about my research system (forests), and climate change poses a threat to the persistence of these ecosystems"). 3) "Societal Impact" captured responses that considered relationships between climate adaptation and human wellbeing, including references to community and human welfare (e.g., "It impacts my community, family, and future"; "Because it is a way I can use my scientific knowledge to aid others"; "Because it impacts all areas of research and livelihoods").

People who identified as marginalized had somewhat significantly different responses from people who identified as not marginalized (Kruskal-Wallis, *p* = 0.052, Fig 2).Fig 2). No respondents from the marginalized group reported a motivation stemming from research or intellectual curiosity alone. There were no significant differences between other groups (e.g., age, career stage, gender).

Fig 2. **Participant motivations for pursuing climate adaptation science.** Reasons for pursuing climate adaptation science were grouped into three categories. There was a somewhat significant difference in responses between people who identified as marginalized and people who identified as not marginalized (Kruskal-Wallis, p = .052). n refers to the sample size of each group.

Support for community members

Formatted: Font color: Black

Acceptance in science and interactions within the scientific community

Overall, the majority of respondents reported feeling supported in the NE CASC community; however, our results indicated that respondents who identify as marginalized have less positive experiences (Fig 3).Fig 3). Respondents strongly or somewhat agreed with the statements "My identity and background are/were supported and accepted in the NE CASC

community" (Fig 3A), "NE CASC members with different backgrounds interact well with each other" (Fig 3F), and "I felt comfortable and safe being myself within the NE CASC community" (Fig 3K). However, community members who identified as marginalized reported less agreement with all three statements (p = 0.003, Fig 3B; p = 0.076, Fig 3G; p = 0.001, Fig 3L). Cisgender women reported less agreement than cisgender men, with significantly fewer "strongly agree" responses to the first and second statements (p = 0.050, Fig 3E; p = 0.033, Fig 3J). Early career fellows reported less agreement than non-fellows, with fewer "strongly agree" responses to the second and third statements (p = 0.071, Fig 3I; p = 0.044, Fig 3). There were no significant differences in agreement with age groups (Fig 3C, H, M).

Fig 3: Participant responses to questions related to acceptance within the NE CASC community. People who identified as marginalized, fellows, and women often reported feeling less supported, less comfortable with community interactions, and less comfortable being themselves. Significant differences (p < 0.05) are indicated with a *, somewhat significant differences (p < 0.10) are indicated with a †. Numbers in boxplots indicate the total number of respondents. "Total" refers to all survey responses, regardless of demographic breakdown; "marg." and "not marg." refer to the self-identified marginalized and not-marginalized demographic responses; "<45 yr" and "≥45 yr" refer to the age breakdown; "fell." and "non-fell." refers to NE CASC fellows (graduate students and postdoctoral scholars) and non-fellows ~~breakdown~~; "cis. w." and "cis. m." refers to the cisgender women and cisgender men demographic breakdowns, with no other genders self-identified in the survey.

Formatted: Font: Arial Unicode MS

Formatted: Font: Gungsuh

Formatted: Font: Gungsuh

Formatted: Font: Gungsuh

Support for research, training, and engagement

Overall, our results indicated that NE CASC community members felt well supported. However, similar to responses related to acceptance, people who identified as marginalized felt significantly less supported as climate adaptation scientists in the NE CASC community (~~Fig 4).~~Fig 4). Respondents strongly or somewhat agreed with the statements “my research and research goals were supported by the NE CASC” (Fig 4A) and “my professional and professional development goals were supported by the NE CASC” (Fig 4F). Generally, respondents only somewhat agreed with the statement “my outreach and stakeholder engagement goals were supported by the NE CASC” (stakeholder engagement is a focus of NE CASC work; Fig 4K). In all cases, people who identified as marginalized were less likely to feel supported ($p = 0.019$, Fig 4B; $p = 0.092$, Fig 4G; $p = 0.035$, Fig 4L). Women also reported feeling significantly less supported than men in both research ($p = 0.015$, Fig 4E) and professional development ($p = 0.004$, Fig 4J).

Fig 4. Responses to how well (A-E) research, (F-J) professional development, and (K-O) stakeholder engagement goals were supported by the NE CASC. In all cases, people who identified as marginalized were less likely to agree that they were supported. Significant differences (p < 0.05) are indicated with a *, somewhat significant differences (p < 0.10) are indicated with a †. Numbers in boxplots indicate the number of respondents.

Formatted: Font: Arial Unicode MS

Commitment to DEI from supervisors and leadership

Overall, respondents strongly or somewhat agreed that their direct supervisors and NE CASC leadership were committed to diversity, equity, and inclusion (Fig 5). While respondents strongly agreed that their supervisors were committed to DEI (Fig 5A), they only somewhat

agreed that NE CASC leadership was committed to DEI (Fig 5K). Younger people (respondents <45 years old) and fellows were somewhat less likely to strongly agree that their direct supervisor was committed to DEI (*p* = 0.097, Fig 5C; *p* = 0.075, Fig 5D). There were no significant differences between other groups in terms of how well direct supervisors were perceived to handle matters of DEI (Fig 5G-J). People who identified as marginalized and fellows were significantly less likely to agree that NE CASC leadership is committed to DEI (*p* = 0.016, Fig 5L; *p* = 0.022, Fig 5N).

Fig 5. Respondent perceptions of direct supervisor commitment to diversity, equity, and inclusion (A-E), how well supervisors handled matters related to diversity, equity, and inclusion (F-J), and whether leadership was perceived as supporting diversity (K-O). People who identified as marginalized were significantly less likely to view diversity as a priority to leadership. Significant differences ($p < 0.05$) are indicated with a *, somewhat significant differences ($p < 0.10$) are indicated with a †. Numbers in boxplots indicate the number of respondents.

Likelihood of leaving scientific research or academia

Overall, respondents strongly disagreed that they have considered leaving academia or scientific research over the last year, including considering leaving due to their identity (~~Fig 6).~~Fig 6). Consistent with previous questions, people who identified as marginalized and fellows showed less disagreement, with the median response for people who identified as marginalized being somewhat agreeing with considering leaving science (*p* = 0.045; Fig 6B). People who identified as marginalized and fellows also showed less disagreement with the statement that

Formatted: Font: Arial Unicode MS

Formatted: Font: 12 pt

Formatted: Font: 12 pt

they have considered leaving scientific research due to their identity (p = 0.022, Fig 6G; p < 0.001, Fig 6I).

Fig 6. Responses to whether respondents had recently considered leaving academia (A-E), including considering leaving due to their identity (F-J). People who identified as marginalized and fellows were significantly more likely to have considered leaving academia, including due to their identity. Significant differences ($p < 0.05$) are indicated with a *, somewhat significant differences ($p < 0.10$) are indicated with a †. Numbers in boxplots indicate the number of respondents.

Formatted: Font: Arial Unicode MS

Barriers and priorities for DEI action

Formatted: Font color: Black

Barriers

There were insufficient data to compare Likert scale responses to “What are the challenges you are most concerned about when it comes to moving forward with your own DEI efforts?” between groups. However, there was a consistent pattern in challenges to DEI efforts across all respondents; sustainability of DEI programming, available time, available funding, and support from the participant’s respective academic institution were generally reported as larger challenges. Support from direct supervisors was reported as being a significantly smaller barrier than all other challenges except for support from NE CASC leadership (Fig 7).

Field Code Changed

Fig 7. Challenges faced by NE CASC community members for advancing their own DEI efforts ordered from largest to smallest. Box plots include all respondents (both marginalized

and not marginalized) due to low sample sizes (white numbers). Letters at the top represent significant (a vs. b; $p<0.05$) or non-significant (a vs. a) differences in the size of the challenge.

In coding open answer responses to the question about concerns and challenges of institutional DEI work, we identified three major themes among answers from individuals who identified as marginalized. First, respondents were concerned about lack of capacity. This included concerns that graduate students of color shoulder the burden of DEI work in science fields,; that there is an absence of planning for sustainable DEI work,; and that there is a lack of available funding to support long-term DEI efforts. Second, respondents wrote that a focus on equity was equally or more important than a focus on increasing diversity, for example: “[My] biggest concern is that [institutions] will continue to focus on diversity, without making any progress on equity, inclusion, and justice. This translates into continuing to bring people from marginalized backgrounds into a hostile workplace/environment.” Finally, distrust of institutions and current DEI work was seen in comments such as, “I'm not confident that the current members of NE CASC feel the urgency/have the capacity of improving their cultural competency in a timely manner that's relevant for current/future students”, and “…the community are more interested in performative activism than actually dismantling the structural racism within the organization and within the greater climate science academic community.”

Future priorities

Overall, responses to the question of future priorities for DEI efforts were low (n = 15 to 27, response rate varied by question based on priority subcategory). Respondents who identified as marginalized ranked “funding existing DEI efforts”, “funding undergraduate students”,”, and “including DEI efforts in job responsibilities” among their top priorities for future actions,

whereas community members from non-marginalized backgrounds ranked “creating clearer DEI policy” as their top priority (~~Fig 8).~~Fig 8). “Funding undergraduate students” was a significantly higher priority for marginalized than non-marginalized groups (*p* = 0.043; ~~Fig 8B).~~Fig 8B). When comparing between priorities, people who identified as marginalized ranked “supporting professional development activities” lower than “funding existing DEI efforts” (*p* = 0.064) and “funding undergraduate students” (*p* = 0.081). There were no significant differences between priorities for people who did not identify as marginalized.

Fig 8. DEI priorities for marginalized and not-marginalized participants. Priorities for supporting DEI efforts ordered by median response by people who identified as coming from historically marginalized groups. Respondents who identified as coming from a historically marginalized group were significantly more likely to select the funding of undergraduate students as a high priority. Significant p-values are indicated with a *. Numbers in the boxplots indicate the number of respondents.

In response to an open-ended question asking people who identified as marginalized what they needed in order to feel more supported in their work, respondents focused on building capacity and equity. For example, respondents stated the NE CASC should “be clearer about funding opportunities”, hire a professional DEI consultant, and “pay more for the cultural taxation of having to teach our over-represented colleagues.” To make the workplace more equitable and inclusive, one respondent suggested efforts to “train faculty and staff better in inclusivity to create a more welcoming environment.” Another response similarly highlighted the need to focus on equity before diversity, “I think too much of the focus of this work is about

‘how can we do better in the future,’ but that aspiration is not built on an adequate foundation of a deep understanding of what is causing minoritized people to struggle in climate adaptation science. NE CASC could do more to acknowledge the challenges faced by underrepresented groups, which includes acknowledging how our systems perpetuate those challenges.”

Discussion

Our survey shows that the NE CASC community as a whole feels supported. However, in both peeling away the majority perspective from Likert-based survey responses and in focusing on anonymous long-answer responses, this analysis highlights how community members from marginalized groups feel less welcomed and supported in environmental sciences. People who identified as marginalized reported significantly less positive experiences from their non-marginalized peers. In particular, people who identified as marginalized, women, and early career fellows reported less agreement that scientists from different backgrounds in the NE CASC community interacted well with each other. People who identified as marginalized and women reported less agreement that they felt their identities were accepted in the NE CASC community, while people who identified as marginalized and fellows reported less agreement that they felt comfortable being themselves in the NE CASC community. While NE CASC is only a portion of most respondent’s experience with science, these results clearly demonstrate that our science institutions are not creating the same experiences for all people.

Long-answer responses from survey participants indicated lack of trust in leadership and in existing DEI efforts to address issues of systemic racism. Further, responses identified concern that recruiting diverse community members would continue to be prioritized over efforts towards creating a more equitable, inclusive, and just workplace for current community members. Indeed,

according to a synthesis of over forty years of publicly available demographic data in the geosciences across degree-levels, Bernard and Cooperdock (2018) highlighted that efforts focused primarily on recruitment and increasing diversity have failed to improve demographics of underrepresented minorities in the geosciences in four decades, and that programs need to revise strategies of improving diversity in the field (~~11~~12). Previous research across scientific fields has shown that people from historically marginalized groups often experience uncomfortable working environments and have little trust in institutions to address systemic racism and inequities (Bernard & Cooperdock, 2018; Marín-Spiotta et al., 2020; Ruf, 2020; Schusler et al., 2021; Taylor, 2018), potentially ~~explaining why~~indicating that efforts focused on recruitment often fail to address issues of diversity in the field by not addressing barriers to retention.

When asked how supported they felt in both their professional and service and outreach works, participants expressed differing perspectives. People from marginalized backgrounds and cisgender women both agreed less that their research goals and professional goals were supported when compared to non-marginalized scientists and cisgender men. However, cisgender women felt equally as supported as men in their service and outreach work, while marginalized people reported feeling less supported in outreach and service work. Service work is often expected of women, and may reduce how supported they feel as scientists (~~Guarino & Borden, 2017).~~42). Climate surveys of other ecology ~~at~~and STEM departments have also revealed perceptions among participants of unequal participation in service work (6,~~36).~~ 43). Within STEM departments, racially minoritized women are more likely than racially minoritized men to engage in diversity-related service work, and have reported feeling unsupported in this role (~~Perez et al. 2022~~43). Despite comprising the majority of our respondents, cisgender women

reported significantly lower feelings of acceptance and support for their research when compared to cisgender men. This information would be lost if representation (52% of survey respondents were women) was our only consideration in assessing community wellness. Our results demonstrate that representation does not preclude inequities among historically marginalized groups, suggesting that efforts focused solely on representation, recruitment, and increasing diversity may still fail to create equitable working conditions, which are crucial to retention of diverse scientists across a range of backgrounds.

While all respondents, both those who identified as marginalized and those who did not, expressed confidence that their direct supervisors were committed to ~~DEI activities~~diversity, equity, and inclusion, they were less confident about the commitment of institutional leadership to advancing DEI through actions. While ‘commitment to DEI’ is somewhat open to interpretation, based on written responses, this might have been interpreted as support for DEI actions and/or long-term commitment to work on breaking down barriers to a diverse, equitable, and inclusive scientific enterprise. For people from historically marginalized groups, distrust of leadership may stem from their historical exclusion from the ecology and environmental science fields (~~27,~~28,29), the field’s historical colonization of natural spaces and sciences without consideration for resident and indigenous perspectives and practices (~~27~~29), leadership focus on performative rather than sustainable actions (~~36~~43), and co-opting of grassroots efforts by administrators (~~36~~43). Rebuilding trust may require demonstrated actions by institutional leadership to support sustainable programming and long term commitment to inclusive and equitable spaces. This may include a focus on enhancing positive interactions with current faculty, staff, and researchers to foster inclusive environments ~~(37,38~~ (44,45). In their long answers, respondents suggested hiring a professional DEI consultant to support efforts,

potentially to help create program plans. In a national study of environmental NGOs, organizations with developed program plans that included concrete goals, incentives, and community buy-in were more successful in their diversity outcomes (~~39).~~ 46). Thus, development of long-term activities that support diversity, equity, and inclusion in the NE CASC community should lead to more positive experiences in climate adaptation science and better retention of researchers and staff. For example, since this survey, NE CASC has set up a DEI committee that meets regularly; consistently funded graduate DEI fellows to build a more inclusive community; established a travel grant to support graduate students and postdoctoral scholars participation in conferences and workshops in order to increase DEI; and has co-produced a statement describing its long-term commitment and actions towards improving DEI.

Discomfort and distrust, in addition to systemic inequities, may be driving marginalized scientists out of the field. Building community and actively creating inclusive environments can help to increase belonging and ultimately retention (45,47). For example, interactions with near-peer and expert in-group mentors can help to increase retention of people from marginalized groups (36,48–50). Respondents who identified as marginalized were more likely than other groups to agree that they had considered leaving academia in the last year, although these respondents also disagreed that they had considered leaving due to their identity or interest. As we have noted above, scientists from marginalized backgrounds also felt less supported than their non-marginalized peers. While we cannot derive causation, feeling less supported may contribute to scientists who identified as marginalized considering leaving academia. These community members may essentially be saying “it’s not me, it’s *you*” and are considering leaving science and academia due to an unsupportive working environment or a failure of the field to support research goals and interests. For example, despite high levels of interest,

capability, and enrollment in environmental science and ecology, undergraduate BIPOC students have high rates of attrition in these majors (1317), which may be related to reported discrimination, feelings of isolation, and dissatisfaction with social perspectives and social consciousness in delivered curricula (5,2729). While the NE CASC mainly supportsattrition was not quantified in this study, our results indicate that similar barriers may exist for graduate students, postdocspostdoctoral scholars, staff, and principal investigators, similar feelings may emerge if inequitable institutional structures also affect people at these levels. Building that are part of the survey community and actively creating inclusive environments can help to increase belonging and ultimately retention (38,40). For example, interactions with near-peer and expert in-group mentors can help to increase retention of people from marginalized groups (41–44). of this study.

Respondents from marginalized groups had different reasons for pursuing climate science and ecology.Our results suggest that socially minded priorities persist for marginalized scientists beyond undergraduate science courses. Shifting focus to applications of research that investigate impacts of environmental issues on humans and non-human beings, rather than abstract scientific “coolness”, could help to attract scientists from more backgrounds (2). Respondents from marginalized groups were more likely to have socially minded reasons for pursuing climate and environmental science compared their non-marginalized peers. Incorporating these motivations into curriculum development, seminar series, and departmental discussions may improve inclusion of theirsocial impact interests and promote overall retention. Similar to Schusler et al. (5), we found that people from marginalized groups cited either social or conservation related reasons for pursuing their research rather than intellectual curiosity alone. In contrast, one-third of non-marginalized scientists cited intellectual curiosity as their primary reason for pursuing the

field. Differences in priorities may lead to a misalignment in activities aimed at recruiting and supporting people from marginalized groups in ecology and environmental sciences~~.~~, which may prevent scientists from feeling professionally satisfied and prompt them to leave the field. Unfortunately, the history of ecology and environmental science has been highly colonial; natural spaces were often brutally seized from Indigenous peoples in the name of "exploration" and "scientific discovery" (~~27,45~~29,51). For example, geologic mapping was a primary weapon of colonization, wherein resource maps provided by geologists to the U.S. government were used as intellectual weapons to justify the forced removal of Indigenous groups (despite evidence in colonizer geologic notes that Indigenous groups held superior knowledge in regional topography and ecology~~) and~~). The scientists who engaged in these colonial practices are often held up as trailblazers in required coursework ~~(45~~(51). Thus, while people from marginalized groups share an interest in the ecology and environmental sciences, curriculum failures and others' perceptions of who belongs in the field may deter diverse scientists from entering or staying in environmental sciences (~~27~~29). As ~~ours~~our and other studies show, marginalized scientists are more likely to have socially and community minded reasons for pursuing environmental research (5), but social and environmental justice disciplines have traditionally been considered separate from ecological and environmental departments (5,~~27).~~29), which means that marginalized scientists who are interested in climate and environmental science but also have social motivations may prefer departments with a stronger interdisciplinary research focus. BIPOC students and scholars have cited efforts to decolonize environmental science curricula and incorporate social perspectives as one way to address issues of inclusion (5,~~27~~29). Our results suggest that socially minded priorities persist for marginalized scientists beyond undergraduate science courses. Shifting focus to applications of research to humans and non-human beings,

rather than abstract scientific "coolness", could help to attract scientists from more backgrounds (2).

Scientists from marginalized groups ranked funding for undergraduate students as a top priority, while scientists from non-marginalized groups ranked funding for undergraduate students as their lowest priority and ranked DEI policy as their top priority. ~~Scientists~~Many NE CASC researchers supervise and rely on undergraduate students to support their research as research and field assistants, though few of these undergraduate researchers are formally involved in NE CASC activities. For undergraduate students, internships and technician experiences are crucial to advancement in natural science careers as these experiences are often considered in admission to graduate schools and other scientific positions (12,52–54). Unpaid and underpaid undergraduate positions are prevalent in the environmental sciences due to funding limitations and outdated attitudes among researchers that unpaid work is a rite of passage (53,55); this practice may exclude people who may have inadequate financial support, different abilities, or responsibilities at home (12,56), which reduces diversity in the field. A nationwide survey of undergraduate students demonstrated that pay below minimum wage was the largest barrier to field and research experiences, followed by incompatible scheduling and non-inclusive work environments (54). Even when paid positions are available, they are extremely competitive and often require previous experience; the ability to take an unpaid position may reflect a person's connections within the field and has been associated with a greater likelihood of persisting in STEM (53). In our survey, scientists from marginalized groups may have seen a lack of funded undergraduate research opportunities as a barrier to their own career paths or to their attempts to hire research assistants from diverse groups.~~ On the other hand, scientists~~Scientists from non-marginalized backgrounds may see unpaid internships as a

rite of passage based on their own experiences and may fail to consider their negative impacts (46,47). For undergraduate students, internships and technician experiences are crucial to advancement in natural science careers as these experiences are often considered in admission to graduate schools and other scientific positions (11,46,48,49). The prevalence of unpaid and underpaid undergraduate positions in environmental sciences excludes people who may have inadequate financial support, different abilities, or responsibilities at home (11,50). A nationwide survey of undergraduate students demonstrated that pay below minimum wage was the largest barrier to field and research experiences, followed by incompatible scheduling and non-inclusive work environments (49). Even when paid positions are available, they are extremely competitive and often require previous experience; the ability to take an unpaid position may reflect a person's connections within the field and has been associated with a greater likelihood of persisting in STEM (46).53,55). In response to these survey results, NE CASC has begun participating in a USGS-funded paid summer research program for undergraduates called Climate Adaptation Scientists of Tomorrow and to date has hosted nine undergraduate researchers at UMass. Increased funding for undergraduate students may support access to research opportunities and field experiences for a more diverse range of students. facing systemic financial barriers.

Sustainable university DEI programs require faculty involvement and sustained funding, as graduate students are only transiently involved. Inclusion of DEI service work in job responsibilities along with recognition and reward structures for participation in DEI service work may help spur other individuals to get involved. In our study, people from marginalized backgrounds called for more funding for DEI work while also noting concerns with the sustainability of DEI work that they perceived to be led primarily by graduate students of

color. Unequal participation in service work is a known issue in academia; in a study of 140 institutions, being a female faculty member was associated with performing significantly more service work across academic rank, race/ethnicity, and field (~~51~~42). Within STEM departments, Perez et al. (~~36~~43) found that racially minoritized (Black, Latino/Hispanic~~/Latinx~~, Native American, Asian American, Multiracial) women graduate students were “compelled to initiate change and engage in labor to advance diversity, equity, and inclusion because they could not wait on faculty to act”, and that this investment “negatively affected their well-being and academic success, limited their access to professional opportunities, and unintentionally, perpetuated faculty inaction” (~~36~~43). Graduate students in these roles also noted unacknowledged emotional labor, like addressing microaggressions and mentoring other students. As a result, racially minoritized women find themselves in a no-win situation: if they do not act they must work in a toxic environment, and if they do act they are chastised for spending too much time on DEI initiatives or their accomplishments are co-opted by faculty (~~36). Sustainable~~43). ~~DEI programs require faculty involvement and sustained funding, as graduate students are only transiently involved. Inclusion of DEI service work in job responsibilities along with recognition and reward structures for participation in DEI service work may help spur other individuals to get involved.~~

It is important to note that while this study references the broad category of “identifies as marginalized”, and the singular categories of gender, age, and career stage (fellow vs. non-fellow), the role of intersectionality in the experiences of survey participants is a crucial one that could not be quantitatively examined while maintaining survey confidentiality. Despite lacking a method for quantitative evaluation, we emphasize the potential role of intersectionality in the responses of this survey. Interpreting the results from a solely gender or racial viewpoint would

potentially exclude the multidimensional experiences of identities of both racial and gender minorities (~~19~~20). Furthermore, gender analysis has historically centered the experiences of white women, as identified by Crenshaw ~~et al. (1989) in her examination of the court case *Moore v. Hughes Helicopter, Inc.*; here, she described~~(20) as the “implicit grounding of white female experiences~~ in the doctrinal conceptualization of sex discrimination~~.” We acknowledge in this study that “gender” as a category may disproportionately center the experiences of white women, while “identify as marginalized” as a category may in a similar way center the experiences of one minority group over those holding intersectional identities that may be “multiply-burdened” (~~Crenshaw, 1989~~20). Therefore, while we continue to discuss “gender” and “identify as marginalized” categories from the survey, we consider the results of the survey with intersectionality in mind, so as to not discuss issues from singularly gender or racial frameworks that would overlook multidimensional challenges faced by intersectional identities.

Conclusions

By allowing individuals to self-identify as historically marginalized in the fields of climate adaptation, ecology, and environmental science, and by centering our analyses on these individuals, we were able to address intersectionality of experiences and highlight voices of historically marginalized members of the NE CASC community~~.~~ and identify some priorities for action. This approach may be especially useful in smaller organizations. While grouping “marginalized” people into one group can have the disadvantage of generalizing and assuming a monolithic experience, in cases of small or organizations with very low representation, we argue that using this tool can help to center voices of marginalized people without identifying individuals. This said, our marginalized category prevents closer examination of specific

experiences of people from marginalized backgrounds who will have unique experiences: for example. a person with a physical disability will face different barriers to inclusion than a BIPOC person. Similarly, this category is limited because we do not know if everyone who is traditionally grouped as "marginalized or underrepresented" based on any demographic factor, also identified as "marginalized or underrepresented" in the survey. Thus, while this term can support intersectional analysis, it does limit interpretation. Further, when demographic data are used to isolate "marginalized" individuals, institutions become responsible for deciding who is heard and who is not. Allowing survey participants to self-identify as coming from a historically marginalized group allows an assessment of how respondents from these backgrounds feel without displaying identifiable data or making assumptions about background and experience. We used this method to highlight the voices and needs of the NE CASC community, which allowed for more targeted action steps to support DEI goals. Our methods may be useful for other small organizations who want to complete similar climate studies.

Based onInformed by this survey, NE CASC leadership have committed to long-term action in support of DEI.- These actions include continuing to fund graduate DEI fellows to focus on building opportunities for peer-mentoring and leading discussion groups of DEI-related research, adding funds for a travel grant to subsidize conference travel for fellows from historically marginalized groups, continuing to survey the NE CASC community to understand changing priorities on DEI, hiring a consultant to evaluate and suggest strategies to improve how PIs mentor graduate students and postdocs, and hosting undergraduate research students from historically marginalized groups through the USGS' 'Climate Adaptation Scientists of Tomorrow' program. While NE CASC represents only a portion of most students' experience in

science, the center aims to increase support and sense of belonging in the hopes of creating a similarly positive experience for all community members.

Acknowledgements

All opinions expressed in this paper are the authors and do not necessarily reflect the policies and views of DOI or USGS. Any use of trade, firm or product names is for descriptive purposes only and does not imply endorsement by the U.S. Government. We are grateful to the NECASC ~~community~~consortium for supporting this work and participating in our survey. We would also like to thank the staff at the University of Massachusetts Office of Equity and Inclusion inspiring our use of a survey and reviewing initial plans for survey execution.

Author Contributions

Meghna N. Marjadi contributed to conceptualization, survey development, analysis, figure creation, writing, and editing. Rebecca A. Smith contributed to analysis, figure creation, writing, and editing. Hsin Fei Tu contributed to survey data organization, analysis, writing, and editing. Asha M. Ajmani contributed to survey development, writing, and editing. Addie Rose Holland contributed to survey development, writing, and editing. Bianca E. Lopez contributed to survey development, writing, and editing~~;~~. Toni Lyn Morelli contributed to survey development, writing, and editing. Bethany A. Bradley contributed to conceptualization, survey development, analysis, figures, writing, and editing.

Conflict of Interest Statement:

This was a community based research project. All the authors have or are members of the NECASC community and have been either directly or indirectly funded by the center to complete this study or other research. The authors do not declare any other conflicts of interest related to this publication.

Formatted: Pattern: Clear, Highlight

Formatted: Font: Arial, 11 pt

Formatted: Space Before: 12 pt, After: 12 pt, Line spacing: Double

References

1. Beck C, Boersma K, Tysor CS, Middendorf G. Diversity at 100: women and underrepresented minorities in the ESA. Front Ecol Environ. 2014;12(8):434–6. .https://tos.org/oceanography/article/unpaid-internships-are-a-barrier-to-diverse-and-equitable-recruitment-in-marine-science
2. Bowser G, Cid CR. Developing the ecological scientist mindset among underrepresented students in ecology fields. Ecol Appl. 2021;31(6):e02348.

33. Cech EA. The intersectional privilege of white able-bodied heterosexual men in STEM. Sci Adv. 2022 Jun 15;8(24):eabo1558.

4. Kou-Giesbrecht S. Asian Americans: The Forgotten Minority in Ecology. Bull Ecol Soc Am. 2020;101(3):1–5.

4. Díaz S, Settele J, Brondízio ES, Ngo HT, Agard J, Arneth A, et al. Pervasive human-driven decline of life on Earth points to the need for transformative change. Science. 2019;366(1327).

5. Schusler TM, Espedido CB, Rivera BK, Hernández M, Howerton AM, Sepp K, et al. Students of colour views on racial equity in environmental sustainability. Nat Sustain. 2021;
6. Barrile GM, Bernard RF, Wilcox RC, Becker JA, Dillon ME, Thomas-Kuzilik RR, et al. Equity, community, and accountability: Leveraging a department-level climate survey as a tool for action. PLOS ONE. 2023 Aug 17;18(8):e0290065.
7. Cantor D, Fisher B, Chibnall S, Townsend R, Lee H, Bruce C, et al. Report on the AAU Campus Climate Survey on Sexual Assault and Sexual Misconduct. Rockville, Maryland: The Association of American Universities, Westat; 2019 p. 439.

Formatted: Font: 12 pt

Formatted: Normal, Indent: Left: 0", Hanging: 0.27", No widow/orphan control, Border: Top: (No border), Bottom: (No border), Left: (No border), Right: (No border), Between : (No border)

Formatted: Highlight

Formatted: Highlight

Formatted: Normal, Indent: Left: 0", Hanging: 0.27", No widow/orphan control, Border: Top: (No border), Bottom: (No border), Left: (No border), Right: (No border), Between : (No border)

Formatted: Highlight

Formatted: Normal, Indent: Left: 0", Hanging: 0.27", No widow/orphan control, Border: Top: (No border), Bottom: (No border), Left: (No border), Right: (No border), Between : (No border)

Formatted: Highlight

Formatted: Highlight

Formatted: Highlight

~~8~~8. Ruf J. Why Environmental Studies is Among the Least Diverse Fields in STEM. Diverse: Issues in Higher Education. 2020 Feb 6;36(25):16–8.

9. Taylor DE. The State of Diversity in Environmental Organizations: Mainstream NGOs, Foundations and Government Agencies [Internet]. Green 2.0; 2014. Available from: www.diversegreen.org/report

Formatted: Normal, Indent: Left: 0", Hanging: 0.27", No widow/orphan control, Border: Top: (No border), Bottom: (No border), Left: (No border), Right: (No border), Between : (No border)

Formatted: Highlight

~~9~~10. Díaz S, Settele J, Brondízio ES, Ngo HT, Agard J, Arneth A, et al. Pervasive human-driven decline of life on Earth points to the need for transformative change. Science. 2019;366(1327).

11~~. Ruf J. Why Environmental Studies is Among the Least Diverse Fields in STEM. Diverse: Issues in Higher Education. 2020 Feb 6;36(25):16–8.~~

~~10~~. Otto IM, Reckien D, Reyer CPO, Marcus R, Le Masson V, Jones L, et al. Social vulnerability to climate change: a review of concepts and evidence. Reg Environ Change. 2017 Aug 1;17(6):1651–62.

Formatted: Normal, Indent: Left: 0", Hanging: 0.27", No widow/orphan control, Border: Top: (No border), Bottom: (No border), Left: (No border), Right: (No border), Between : (No border)

Formatted: Highlight

~~11~~12. Bernard RE, Cooperdock EHG. No progress on diversity in 40 years. Nat Geosci. 2018 May;11(5):292–5.

Formatted: Highlight

~~12~~13. Freeman J. LGBTQ scientists are still left out. Nature. 2018 Jul;559(7712):27–8.

Formatted: Highlight

~~13. Taylor DE. Enhancing racial diversity in the Association for Environmental Studies and Sciences. J Environ Stud Sci. 2018 Dec 1;8(4):379–84.~~

14. Cech EA. LGBT Professionals' Workplace Experiences in STEM-Related Federal Agencies. In 2015 [cited 2023 Sep 22]. p. 26.1094.1-26.1094.10. Available from: https://peer.asee.org/lgbt-professionals-workplace-experiences-in-stem-related-federal-agencies

Formatted: Normal, Indent: Left: 0", Hanging: 0.27", No widow/orphan control, Border: Top: (No border), Bottom: (No border), Left: (No border), Right: (No border), Between : (No border)

Formatted: Highlight

15. Cech EA, Pham MV. Queer in STEM Organizations: Workplace Disadvantages for LGBT Employees in STEM Related Federal Agencies. Soc Sci. 2017 Mar;6(1):12.

16. Landivar LC. Disparities in STEM Employment by Sex, Race, and Hispanic Origin [Internet]. Washington, D.C.: U.S. Census Bureau; 2013 [cited 2023 Sep 25]. Report No.: ACS-24. Available from: https://www.census.gov/library/publications/2013/acs/acs-24.html

~~17~~17. Taylor DE. Enhancing racial diversity in the Association for Environmental Studies and Sciences. J Environ Stud Sci. 2018 Dec 1;8(4):379–84.

18. Cech EA, Waidzunas TJ. Systemic inequalities for LGBTQ professionals in STEM. Sci Adv. 2021 Jan 15;7(3):eabe0933.

~~18~~19. Directorate-General for Research and Innovation (European Commission). She figures 2021: tracking progress on the path towards gender equality in research and innovation [Internet]. LU: Publications Office of the European Union; 2021 [cited 2023 Sep 22]. Available from: https://data.europa.eu/doi/10.2777/602295

~~19~~20. Crenshaw K. Demarginalizing the Intersection of Race and Sex: A Black Feminist Critique of Antidiscrimination Doctrine, Feminist Theory and Antiracist Politics. Univ Chic Leg Forum. 1989;1989(1):139–67.

~~20~~21. Taylor DE. Race, gender, and faculty diversity in environmental disciplines. In: E. Taylor D, editor. Environment and Social Justice: An International Perspective [Internet]. Emerald Group Publishing Limited; 2010 [cited 2023 Sep 25]. p. 385–407. (Research in Social Problems and Public Policy; vol. 18). Available from: https://doi.org/10.1108/S0196-1152(2010)0000018015

~~21~~22. Kellert SR. American Attitudes Toward and Knowledge of Animals: An Update. In: Fox MW, Mickley LD, editors. Advances in Animal Welfare Science 1984 [Internet]. Dordrecht:

Formatted: Highlight

Formatted: Highlight

Formatted: Normal, Indent: Left: 0", Hanging: 0.27", No widow/orphan control, Border: Top: (No border), Bottom: (No border), Left: (No border), Right: (No border), Between : (No border)

Formatted: Highlight

Formatted: Highlight

Formatted: Highlight

Formatted: Highlight

Springer Netherlands; 1985 [cited 2023 Nov 17]. p. 177–213. Available from: http://link.springer.com/10.1007/978-94-009-4998-0_11

~~22~~23. Stamps SM, Stamps MB. Race, Class and Leisure Activities of Urban Residents. J Leis Res. 1985 Jan 1;17(1):40–56.

~~23~~24. Miriti MN, Rawson AJ, Mansfield B. The history of natural history and race: Decolonizing human dimensions of ecology. Ecol Appl. 2023;33(1):e2748.

~~24~~25. Mohai P. Dispelling Old Myths: African American. Environ Sci Policy Sustain Dev. 2003 Jun 1;45(5):10–26.

~~25~~26. Taylor DE. Racial and Ethnic Differences in Connectedness to Nature and Landscape Preferences Among College Students. Environ Justice. 2018 Jun;11(3):118–36.

~~26~~27. Taylor DE. Racial and ethnic differences in the students' readiness, identity, perceptions of institutional diversity, and desire to join the environmental workforce. J Environ Stud Sci. 2018 Jun 1;8(2):152–68.

~~27. Chaudhury A, Colla S. Next steps in dismantling discrimination: Lessons from ecology and conservation science. Conserv Lett. 2021 Mar;14(2):e12774.~~

28. Marín-Spiotta E, Barnes RT, Berhe AA, Hastings MG, Mattheis A, Schneider B, et al. Hostile climates are barriers to diversifying the geosciences. Adv Geosci. 2020 Jul 28;53:117–27.

~~29.~~29. Chaudhury A, Colla S. Next steps in dismantling discrimination: Lessons from ecology and conservation science. Conserv Lett. 2021 Mar;14(2):e12774.

~~McGee EO. Devalued Black and Latino Racial Identities: A By-Product of STEM College Culture? - Ebony O. McGee, 2016. Am Educ Res J. 2016;53(6):16261662.~~

Formatted: Highlight

Formatted: Highlight

Formatted: Highlight

Formatted: Highlight

Formatted: Highlight

Formatted: Highlight

Formatted: Normal, Indent: Left: 0", Hanging: 0.27", No widow/orphan control, Border: Top: (No border), Bottom: (No border), Left: (No border), Right: (No border), Between : (No border)

Formatted: Highlight

30. Leath S, Chavous T. Black Women's Experiences of Campus Racial Climate and Stigma at Predominantly White Institutions: Insights from a Comparative and Within-Group Approach for STEM and Non-STEM Majors. J Negro Educ. 2018;87(2):125–39.

~~31~~31. McGee EO. Devalued Black and Latino Racial Identities: A By-Product of STEM College Culture? - Ebony O. McGee, 2016. Am Educ Res J. 2016;53(6):16261662.

32. Rainey K, Dancy M, Mickelson R, Stearns E, Moller S. Race and gender differences in how sense of belonging influences decisions to major in STEM. Int J STEM Educ 2018 51. 2018 Apr;5(1):1–14.

33. Spoon K, LaBerge N, Wapman KH, Zhang S, Morgan AC, Galesic M, et al. Gender and retention patterns among U.S. faculty. Sci Adv. 2023 Oct 20;9(42):eadi2205.

34. Krause KH, Woofter R, Haardörfer R, Windle M, Sales JM, Yount KM. Measuring campus sexual assault and culture: A systematic review of campus climate surveys. Psychol Violence. 2019;9(6):611–22.

35. Pritchard AJ, DeKeseredy WS, Nolan J, Hall-Sanchez A. Who Speaks First? Analyzing Response Waves in a Large-Scale Campus Climate Survey. J Aggress Maltreatment Trauma. 2019 Aug 9;28(7):888–901.

36. Stout JG, Dasgupta N, Hunsinger M, McManus MA. STEMing the tide: Using ingroup experts to inoculate women's self-concept in science, technology, engineering, and mathematics (STEM). J Pers Soc Psychol. 2011;100(2):255–70.

37. Miller DC, Salkind NJ. Handbook of Research Design and Social Measurement [Internet]. Sage Publications Inc.; 2002 [cited 2023 Sep 25]. Available from: https://us.sagepub.com/en-us/nam/handbook-of-research-design-and-social-measurement/book10296

~~32~~38. de Winter JCF. Using the Student's t-test with extremely small sample sizes. Pract Assess

Formatted: Normal, Indent: Left: 0", Hanging: 0.27", No widow/orphan control, Border: Top: (No border), Bottom: (No border), Left: (No border), Right: (No border), Between : (No border)

Formatted: Highlight

Formatted: Normal, Indent: Left: 0", Hanging: 0.27", No widow/orphan control, Border: Top: (No border), Bottom: (No border), Left: (No border), Right: (No border), Between : (No border)

Formatted: Highlight

Res Eval [Internet]. 2019 Nov 25;18(1). Available from: https://scholarworks.umass.edu/pare/vol18/iss1/10

~~33~~39. R Core Team. R: A language and environment for statistical computing. [Internet]. Vienna, Austria: R Foundation for Statistical Computing; 2023. Available from: https://www.R-project.org/

~~34. Wikham~~40. Wickham H. ~~gplot2~~ggplot2: Elegant Graphics for Data Analysis. [Internet]. Springer-Verlag. New York~~;~~.: 2016. Available from: https://ggplot2.tidyverse.org.

41. Charmaz K. Constructing grounded theory. London ; Thousand Oaks, Calif: Sage Publications; 2006. 208 p.

42. Guarino CM, Borden VMH. Faculty Service Loads and Gender: Are Women Taking Care of the Academic Family? Res High Educ. 2017 Sep 1;58(6):672–94.~~35. Kruskal WH, Wallis WA. Use of Ranks in One-Criterion Variance Analysis. J Am Stat Assoc. 1952;47(260):583–621.~~

~~36~~

43. Perez RJ, Motshubi R, Rodriguez SL. “We are a huge source of labor”: Exploring STEM Graduate Students’ Roles in Changing Departmental Climate. Rev High Educ. 2022;46(1):33–66.

~~37~~44. Chaudhary VB, Berhe AA. Ten simple rules for building an antiracist lab. PLOS Comput Biol. 2020 Oct;16(10):e1008210.

~~38~~45. Golden N, Devarajan K, Balantic C, Drake J, Hallworth MT, Morelli TL. Ten simple rules for productive lab meetings. PLOS Comput Biol. 2021 May 27;17(5):e1008953.

~~39~~46. Beasley MA. Beyond Diversity: A roadmap to building an inclusive organization [Internet]. Diverse Green; 2017. Available from: https://diversegreen.org/wp-

Formatted: Highlight

Formatted: Highlight

Formatted: Highlight

Formatted: Normal, Indent: Left: 0", Hanging: 0.27", No widow/orphan control, Border: Top: (No border), Bottom: (No border), Left: (No border), Right: (No border), Between : (No border)

Formatted: Highlight

Formatted: Highlight

Formatted: Highlight

content/uploads/2021/01/BeyondDiversity_Report_060517-1.pdf

4047. Yosso T, Smith W, Ceja M, Solórzano D. Critical Race Theory, Racial Microaggressions, and Campus Racial Climate for Latina/o Undergraduates. Harv Educ Rev. 2010 Jan 29;79(4):659–91.

4148. Anadu J, Ali H, Jackson C. Ten Steps to Protect BIPOC Scholars in the Field. Eos [Internet]. 2020 Oct 14 [cited 2023 Nov 17];101. Available from: https://eos.org/opinions/ten-steps-to-protect-bipoc-scholars-in-the-field

4249. Stout JG, Dasgupta N, Hunsinger M, McManus MA. STEMing the tide: Using ingroup experts to inoculate women’s self-concept in science, technology, engineering, and mathematics (STEM). J Pers Soc Psychol. 2011;100(2):255–70.

43. Wolfe BA, Riggs EM. Macrosystem Analysis of Programs and Strategies to Increase Underrepresented Populations in the Geosciences. J Geosci Educ. 2017 Nov;65(4):577–93.

4450. Wu DJ, Thiem KC, Dasgupta N. Female peer mentors early in college have lasting positive impacts on female engineering students that persist beyond graduation. Nat Commun. 2022 Nov 11;13(1):6837.

4551. Pico T. The Darker Side of John Wesley Powell. Scientific American [Internet]. 2019 Sep 9 [cited 2023 Nov 17]; Available from: https://blogs.scientificamerican.com/voices/the-darker-side-of-john-wesley-powell/

4652. Beltran RS, Marnocha E, Race A, Croll DA, Dayton GH, Zavaleta ES. Field courses narrow demographic achievement gaps in ecology and evolutionary biology. Ecol Evol. 2020 Jun;10(12):5184–96.

53. Fournier AMV, Holford AJ, Bond AL, Leighton MA. Unpaid work and access to science professions. Rosenbloom JL, editor. PLOS ONE. 2019 Jun 19;14(6):e0217032.

Formatted: Highlight

Formatted: Highlight

Formatted: Highlight

Formatted: Normal, Indent: Left: 0", Hanging: 0.27", No widow/orphan control, Border: Top: (No border), Bottom: (No border), Left: (No border), Right: (No border), Between : (No border)

Formatted: Highlight

Formatted: Highlight

Formatted: Highlight

Formatted: Normal, Indent: Left: 0", Hanging: 0.27", No widow/orphan control, Border: Top: (No border), Bottom: (No border), Left: (No border), Right: (No border), Between : (No border)

Formatted: Highlight

~~47~~54. Jensen AJ, Bombaci SP, Gigliotti LC, Harris SN, Marneweck CJ, Muthersbaugh MS, et al. Attracting Diverse Students to Field Experiences Requires Adequate Pay, Flexibility, and Inclusion. BioScience. 2021 Jul 1;71(7):757–70.

55. Fournier AMV, Bond AL. Volunteer field technicians are bad for wildlife ecology: Volunteer Field Technicians Are Bad for Wildlife Ecology. Wildl Soc Bull. 2015 Dec;39(4):819–21.

~~48~~56. ~~Beltran RS, Marnocha E, Race A, Croll DA, Dayton GH, Zavaleta ES. Field courses narrow demographic achievement gaps in ecology and evolutionary biology. Ecol Evol. 2020 Jun;10(12):5184–96.~~

~~49. Jensen AJ, Bombaci SP, Gigliotti LC, Harris SN, Marneweck CJ, Muthersbaugh MS, et al. Attracting Diverse Students to Field Experiences Requires Adequate Pay, Flexibility, and Inclusion. BioScience. 2021 Jul 1;71(7):757–70.~~

~~50~~. Kreuser A, Bishop A, Meyer-Gutbrod E. FROM THE TOS JEDI COMMITTEE: Unpaid Internships Are a Barrier to Diverse and Equitable Recruitment in Marine Science. Oceanography [Internet]. 2023 May 25 [cited 2023 Aug 14]; Available from: https://tos.org/oceanography/article/unpaid-internships-are-a-barrier-to-diverse-and-equitable-recruitment-in-marine-science

~~51~~

Formatted: Normal, Indent: Left: 0", Hanging: 0.27", No widow/orphan control, Border: Top: (No border), Bottom: (No border), Left: (No border), Right: (No border), Between : (No border)

Formatted: Highlight

Formatted: Normal, Indent: Left: 0", Hanging: 0.27", No widow/orphan control, Border: Top: (No border), Bottom: (No border), Left: (No border), Right: (No border), Between : (No border)

Formatted: Highlight

Supporting Information

S1 File. Survey questions, format, and answers. PDF of Survey questions, formats and answers.

S2 File. Anonymized survey data. Anonymized data from our study figures and analyses for each reported question and figure. . Guarino CM, Borden VMH. Faculty Service Loads and Gender: Are Women Taking Care of the Academic Family? Res High Educ. 2017 Sep 1;58(6):672–94.

Formatted: Not Highlight

Formatted: Line spacing: Multiple 1.15 li, Widow/Orphan control, Border: Top: (No border), Bottom: (No border), Left: (No border), Right: (No border), Between : (No border)

Dear Editors,

Thank you for your consideration of our manuscript. We have made improvements to our manuscript to address the reviewer comments, which we have responded to below. Additionally, we completed a separate review with the United States Geological Survey (USGS) since one of our authors is affiliated (TLM). We have included those comments and our responses to the USGS review at the end of the rebuttal letter. As requested in the additional comments and journal requirement:

1. We have done our best to adhere to journal formatting requirements based on the provided links.
2. We have updated our financial disclosures to reflect the involvement of funders:
    a. "This research was supported in part by the U.S. Geological Survey, Northeast Climate Adaptation Science Center (NE CASC) through Grant No. G19AC00091. This study was a participatory project which involved researchers, staff, and graduate students at the NE CASC. Additionally, MNM was supported by a Switzer Foundation fellowship while working on this project and is currently supported by NOAA Cooperative Institute for the North Atlantic Region (Grant No. NA19OAR4320074); these funders had no role in study design, data collection and analysis, decision to publish, or preparation of the manuscript."
3. We have reviewed the data sharing agreements. Our study collected sensitive demographic data data (including disability status, race, and age) from a very small group, which means that individuals could potentially be identified if data are shared. As per our IRB agreement (#2705), we can only provide these data in the aggregate. We have done so in our paper and supplemental materials.
4. We have included captions for the supporting information files and have updated in-text citations.

    Thank you for your consideration of our manuscript for publication. Please let us know if we can provide additional information.

Best wishes,
Meghna N. Marjadi

Responses to comments:

PloS Reviewer's Responses to Questions
Comments to the Author

1. Is the manuscript technically sound, and do the data support the conclusions?

The manuscript must describe a technically sound piece of scientific research with data that supports the conclusions. Experiments must have been conducted rigorously, with appropriate controls, replication, and sample sizes. The conclusions must be drawn appropriately based on the data presented.
Reviewer #1: Yes
Reviewer #2: Partly

2. Has the statistical analysis been performed appropriately and rigorously?
Reviewer #1: Yes
Reviewer #2: Yes

---

3. Have the authors made all data underlying the findings in their manuscript fully available?

The PLOS Data policy requires authors to make all data underlying the findings described in their manuscript fully available without restriction, with rare exception (please refer to the Data Availability Statement in the manuscript PDF file). The data should be provided as part of the manuscript or its supporting information, or deposited to a public repository. For example, in addition to summary statistics, the data points behind means, medians and variance measures should be available. If there are restrictions on publicly sharing data—e.g. participant privacy or use of data from a third party—those must be specified.
Reviewer #1: Yes
Reviewer #2: No
>>We recognize that the underlying data for the survey are not available. However, per IRB rules, we are not able to share the data because of risk of compromising respondent identities.

---

4. Is the manuscript presented in an intelligible fashion and written in standard English?

PLOS ONE does not copyedit accepted manuscripts, so the language in submitted articles must be clear, correct, and unambiguous. Any typographical or grammatical errors should be corrected at revision, so please note any specific errors here.
Reviewer #1: Yes
Reviewer #2: Yes

---

5. Review Comments to the Author

Please use the space provided to explain your answers to the questions above. You may also include additional comments for the author, including concerns about dual publication, research ethics, or publication ethics. (Please upload your review as an attachment if it exceeds 20,000 characters)

Reviewer #1:
1. This is a sound manuscript that adequately presents gaps and data that support the conclusions stated.
    a. Thank you
2. The title is informative and contains relevant and meaningful keywords.
    a. Thank you
3. The final sentence of lines 97-99 is missing a reference, but the introduction adequately summarizes the existing knowledge about the topic and gaps that exist in the literature.
    a. Thank you. We have added references to support this sentence.
4. Study aims are clear though the hypothesis could be framed more clearly.

    a. We feel that the question-based framing at the end of the introduction is most appropriate for this study because we did not have hypotheses about how community members were experiencing and interacting with NE CASC.

5. The methodology while mostly clear needs clarification. What methodology was used for the qualitative coding (line 202)?
    a. We followed grounded theory methodology to complete our coding. We have added more details to the qualitative analysis section of the results.
6. Also, please elaborate on why the Kruskal-Wallis rank sum test was used (line 204).
    a. We used the Kruskal-Wallis test to compare the numbers of responses in each category for the marginalized and non-marginalized groups. We used this non-parametric test to accommodate our small dataset which had an unequal distribution across groups. We have added clarification in this section of the methods.
7. The results and discussion were clear and all figures appeared accurate and were appropriately descriptive.
    a. Thank you

Reviewer #2: This study provided DEI insights in an academic institution setting. A survey was conducted with the Northeast Climate Adaptation Science Center consortium institutions to understand challenges faced by their community members, especially those from underrepresented groups. The study includes some interesting results related to the different motivations presented in the marginalized and non-marginalized groups. The paper can benefit from improvements in the following areas:

1. The introduction section only introduced the importance of understanding diversity in scientific and academic spaces, but failed to demonstrate the knowledge gaps in the area, and how this current study fills in these knowledge gaps. A literature review of similar previous studies in understanding DEI issues in scientific settings is completely missing. What approach has been taken by previous studies in understanding DEI issues, and what did they find as barriers to retention of scientists from historically marginalized backgrounds?
    a. We focused on describing study results in our introduction rather than comparing methodologies. We have included an additional paragraph (paragraph 8) in the introduction to clarify the methods for some of the studies we cited earlier in the introduction. We have discussed barriers to retention in paragraph 6 and 7 of the introduction. We have also discussed these barriers in the context of our results in the discussion. Other surveys have been campus wide, undergraduate focused, or conducted across many STEM disciplines. Our study is unique in three ways: 1) focus on a smaller community with similar research goals and 2) consideration of different career levels, 3) focusing on marginalized communities with an intersectional lens.
2. The introduction also seems to misalign with the actual research conducted. The latter has an emphasis of career stage and age diversity in addition to race and gender diversity, which was mainly discussed in the introduction section. Similarly, the “barriers to retention of scientists from historically marginalized backgrounds” mentioned as a goal in the introduction section isn’t really addressed by this research.

a. We agree that the introduction focuses on race and gender diversity - this was intentional given that our results show that experiences of NE CASC community members primarily differed based on whether respondents identified as part of a marginalized group or whether they identified as cis-women. In the former case, people from marginalized groups tended to feel less supported and were more likely to have considered leaving science. In the latter case, cis-women also tended to feel less supported. Because we did not see consistent differences based on age or career stage, we elected not to frame the introduction based on these identifiers.
    i. Our interpretation of the results related to whether respondents feel accepted and supported in the NE CASC community (Fig 2) and in science more generally (Fig 3) is that these feelings of inclusion are directly related to retention of people from historically marginalized groups. We have added a paragraph to the introduction to better connect feelings of support and belonging to retention of scientists in STEM.

3. Was the survey able to engage any of the tribal members involved in the Center? What specific efforts have been made to engage tribal members in the survey, especially given the Center's emphasis on regional tribal nations?
    a. One participant at the center identified as Indigenous American (as reported in the demographic section). We do not have demographic information for all people affiliated with the center, so we do not know how many Indigenous people did not participate. For this survey, we aimed to engage all members of the center equally to better understand experiences of the NE CASC community from all backgrounds. Much of the past and ongoing Tribal engagement work involves working with Tribal Nations and organizations whose members are not part of the NE CASC community (i.e. not directly supported with funding from the Center). However, partner organizations were not included in the survey.
4. What kinds of DEI initiatives have already been carried out by the Center? What is the Center's vision related to DEI? What motivated the survey? This kind of background is important for the readers to understand the context of and the responses to the survey.
    a. This survey was among the initial DEI initiatives conducted by the Center. The survey was conducted in spring 2021 and the initial funding of 'DEI Fellows' began in fall 2020. The survey was motivated by the online workshop held in December 2020, where a discussion among community members revealed that baseline data about demographics and experiences in science and at NE CASC were necessary to evaluate whether future DEI actions had any effect. We have added background text on NE CASC's DEI activities and motivation to the beginning of the survey development and implementation section.
5. Figure 1: This figure should include breakdowns in terms of age and career stage, as these are important groups being discussed in the subsequent analyses. Similarly, the gender figure needs to have breakdowns of cis-male and female.
    a. Thanks for this suggestion. We have expanded Figure 1 to include the breakdowns of age, career stage, and gender.
6. What constituents disabled? Physical or mental? This is not clear.
    a. We included all types of disabilities in this category. The question in the survey was: "Please indicate any disability status that you may have" and the answers

were: "Yes I have a disability" and "No I do not have a disability". We have included a clarifying sentence in the Demographic Information section of the Methods.

7. The narrative in the results section repeats the information that has already been presented in the figures. It is more important to discuss the analysis and the implications in the results, rather than simply restating the figures.
    a. While some results text and figures might be redundant, we prefer to keep the results text because it allows us to include all of the p values associated with the Kruskal-Wallis tests. The format of Plos ONE has a separate results and discussion section, so we have discussed the analysis and implications in the discussion section.
8. What are the implications of the differences in terms of motivation for pursuing climate adaptation science? How does it relate to the retention of the underrepresented climate scientists?
    a. Scientists from marginalized groups who have social motivations for their climate adaptation research may leave the field if they do not have the resources or support to do that research or if the field does not recognize the importance of social motivations in climate adaptation research. Other research (Schusler et al. 2021) has demonstrated that differences in motivations may contribute to students in environmental science feeling unwelcome and potentially leaving the field. Based on our results, this could also happen for researchers at later stages in their careers. Additionally, traditional funding mechanisms may also reduce opportunities to conduct research with social implications and motivations. We have revised this paragraph in the discussion to make the connections clearer.
9. How does the support for community members influence retention? What are the underlying reasons for the marginalized groups to feel less supported?
    a. Researchers who identified as marginalized were more likely to feel less supported than their non-marginalized peers and also more likely to have considered leaving academia in the last year. While we cannot derive causation, lack of support may be related to considering leaving the field. To make this connection clearer, we have revised the paragraph that starts: "Discomfort and distrust, in addition to systemic inequities, may be driving marginalized scientists out of the field." Additionally, within our own study we do not have long term data to track whether participants left CASC or the field and the survey was anonymous.
    b. We have discussed some of the underlying reasons that scientists may not feel supported in our discussion and literature review. For example, if they feel their motivations for research do not align with department goals. Our survey goal was to assess how community members felt at the center. Some of the underlying issues are related to systemic problems in academia and science, which we have discussed in both the introduction and discussion.

10. What does "commitment to DEI" entail? What kind of commitment already exists? This is the part that really needs some background information about the institutional context of the NE CASC from the DEI perspective.

    a. This is a good question - respondents might have interpreted this question in different ways. Based on open ended responses, it is likely that commitment to DEI was often interpreted as support for activities that build inclusivity, support for trainings and other opportunities that help people learn about diverse perspectives on science, and a long-term commitment to continue to support such activities. We added text to the introduction (in response to comment 4) to better describe the DEI activities supported by NE CASC leading up to this survey - these activities were focused on funding for 'DEI Fellows', whose efforts led to this survey and many years of Tribal engagement. NE CASC had written a response condemning the murder of George Floyd, but did not have another stated commitment to DEI prior to this survey. We have added some interpretation of 'commitment to DEI' to the discussion.

11. "Funding undergraduate students" is hard to understand again without the context. Is there a high percentage of unpaid undergraduate researchers within NE CASC currently? If so, what are the reasons for these students not to be compensated?
    a. We have added additional context in the discussion paragraph that starts "Scientists from marginalized groups ranked funding for undergraduate students as a top priority, while scientists from non-marginalized groups ranked funding for undergraduate students as their lowest priority and ranked DEI policy as their top priority." Unpaid undergraduate opportunities are prevalent in environmental and natural sciences due to funding limitations and the perception among some researchers that they are a 'rite of passage'. Recruiting diverse undergraduate students contributes to a more diverse workforce and can be difficult when many unpaid opportunities exclude diverse researchers. We have rearranged the paragraph and added more context to explain this issue.

USGS Reviewer Comments and responses:

I very much enjoyed the opportunity to read this manuscript and provide some comments. I would definitely recommend the manuscript for publication, were I reviewing it on behalf of a journal. It seems to me that the manuscript identifies a crucial limitation of the utilization of surveys in the DEIA space, not just where there is not a great deal of diversity in the survey pool, but also where there are small number of persons and anonymity becomes impossible to maintain.
I found the findings to be quite timely and relevant. The recommendation to allow participants to self-identify as marginalized is a valuable one, and I hope that it will be replicated.
Thank you! We are grateful for your review and consideration of our manuscript.

The recommendations/comments that I have are as follows:

1. I think the article might benefit for a more in-depth discussion of limitations. For example, the survey was not given to any undergraduate students, nor was it given to non-PIs on funded projects. I would wonder if responses might be at all different among those researchers who were not the PIs.
    a. We provided the survey to all individuals who were formally associated with the NE CASC and its activities. Our study focused on graduate students, postdocs,

and faculty to evaluate department dynamics in the CASC. Undergraduate students are not directly part of the NE CASC, which we have clarified in our methods section. Additionally, undergraduate experiences were not the focus of this study. There are already many studies on STEM experiences and ecology/environmental science experiences that are limited to undergraduate students (Schusler et al., 2021; Taylor, 2018b, 2018c).

   b. As noted, we provided the survey to all individuals who were formally associated with the NE CASC and its programming. We did not intentionally exclude non-PIs on funded projects. In fact, several graduate students and postdocs were non-PIs on funded projects, which was why they were a part of the CASC environment. We did wonder if PIs and non-PIs would have different experiences, this was one reason we made comparisons between fellows/non-fellows. We have clarified this in the methods section. We have added more about this in our introduction. We have also added some discussion of the limitations of using the "marginalized respondents" category to the discussion.

2. Additionally, it is unclear to what extent the self-designation as marginalized overlapped with the demographic characteristics that are typically classed as marginalized.
   a. Yes. We have intentionally not made this comparison explicitly to preserve privacy of participants in accordance with our IRB; however, we have listed specific demographic information alongside the number of self-identified marginalized participants in Figure 1 and in the first paragraph of the demographics section. The answer to this question depends on which demographic one is considering (disability status, gender, sexuality, race, ethnicity, first generation college status, immigration status, socioeconomic background). Part of the goal of our study was to demonstrate that marginalized identities are intersectional and using singular demographic categories may miss the true experiences of community members, which we have discussed in depth in the discussion. For example, women have historically been marginalized in STEM, but while 22 cisgender women replied to the survey, only 10 people identified as historically marginalized. On the other hand, two individuals identified as having a disability (another group that would be historically marginalized in STEM). Additionally, because of the low numbers of survey respondents, we were not allowed to look at the specific breakdowns to preserve respondent privacy. They were collated for us by an outside party.
3. Finally, it is unclear why ten respondents chose not to answer the question regarding if they self-identified as marginalized. This is almost a quarter of the respondents, and just as many as those who had identified as marginalized.
   a. We can only speculate about why these ten respondents did not complete this question in the survey. It is possible that they did not understand or skipped the question accidentally. Demographic questions were at the end of the survey, so it is possible that some respondents closed the survey before completing it. Additionally, we allowed respondents to check "yes" or "no" and some respondents may not have had strong feelings about where they fit. We have added this limitation to the discussion.
4. The article points to the need to address systemic barriers, but it doesn't really indicate how an entity such as NECASC could do so. I know that we have limitations on the

ability to prescribe policy solutions, but it does seem that the problem is larger than the span of control of an organization such as NECASC. Are there recommendations that can be made that address these systemic challenges? Are some of those within the sphere of influence of the CASC? For example, there is explicit discussion of the need to have ecology curriculums not be blind to the harms that were perpetuated by some of the "great men" of the field. Similarly, the article points to the potential for additional barriers for students who, for example, cannot afford to take unpaid or underpaid work. Additional funding for undergraduate programs is mentioned as a potential solution, but perhaps a clearer recommendations section would be beneficial?

a. Addressing systemic challenges may be beyond the scope of this paper and survey. However, we have included an additional paragraph at the end of the discussion to highlight how the NE CASC has considered survey results and taken action. Our goal was to highlight the voices of the scientists who identified as marginalized. As you note, we have highlighted many of these recommendations in the discussion along with justifications based on our results. Previously the recommendations were at the end of each paragraph and may have been missed. Instead of creating a recommendations section, we have moved the recommendations to the beginning of the paragraphs to make them stand out more and allow them to stay with their justifications.
