# Supplementary material for: Centering voices of scientists from marginalized backgrounds to understand experiences in climate adaptation science and inform action
Source: PLoS One. 2025 Feb 21;20(2):e0318438. doi: 10.1371/journal.pone.0318438 (PMC11844896; doi:10.1371/journal.pone.0318438)
Supplement: Attachment — Submitted filename: PLOS_Response_To_Reviewers.docx [file pone.0318438.s003.docx]

Dear Editors,

Thank you for your consideration of our manuscript. We have made improvements to our manuscript to address the reviewer comments, which we have responded to below. Additionally, we completed a separate review with the United States Geological Survey (USGS) since one of our authors is affiliated (TLM). We have included those comments and our responses to the USGS review at the end of the rebuttal letter. As requested in the additional comments and journal requirement:

1. We have done our best to adhere to journal formatting requirements based on the provided links.
2. We have updated our financial disclosures to reflect the involvement of funders:
   a. “This research was supported in part by the U.S. Geological Survey, Northeast Climate Adaptation Science Center (NE CASC) through Grant No. G19AC00091. This study was a participatory project which involved researchers, staff, and graduate students at the NE CASC. Additionally, MNM was supported by a Switzer Foundation fellowship while working on this project and is currently supported by NOAA Cooperative Institute for the North Atlantic Region (Grant No. NA19OAR4320074); these funders had no role in study design, data collection and analysis, decision to publish, or preparation of the manuscript.”
3. We have reviewed the data sharing agreements. Our study collected sensitive demographic data data (including disability status, race, and age) from a very small group, which means that individuals could potentially be identified if data are shared. As per our IRB agreement (#2705), we can only provide these data in the aggregate. We have done so in our paper and supplemental materials.
4. We have included captions for the supporting information files and have updated in-text citations.

Thank you for your consideration of our manuscript for publication. Please let us know if we can provide additional information.

Best wishes,

Meghna N. Marjadi

Responses to comments:

PloS Reviewer's Responses to Questions

Comments to the Author

1. Is the manuscript technically sound, and do the data support the conclusions?

The manuscript must describe a technically sound piece of scientific research with data that supports the conclusions. Experiments must have been conducted rigorously, with appropriate controls, replication, and sample sizes. The conclusions must be drawn appropriately based on the data presented.

Reviewer #1: Yes

Reviewer #2: Partly

2. Has the statistical analysis been performed appropriately and rigorously?

Reviewer #1: Yes

Reviewer #2: Yes

3. Have the authors made all data underlying the findings in their manuscript fully available?

The [PLOS Data policy](https://nam10.safelinks.protection.outlook.com/?url=http%3A%2F%2Fc05y1x9s.r.us-east-2.awstrack.me%2FL0%2Fhttp%3A%252F%252Fwww.plosone.org%252Fstatic%252Fpolicies.action%2523sharing%2F1%2F010f019132625300-265ed855-a7e2-4fe6-94fb-d7bfc9270c60-000000%2FUkelw6B9cDubtYSic0pqXfJXsUY%3D171&data=05%7C02%7Cbbradley%40eco.umass.edu%7Ce6f87c4111df4f39687e08dcb7b87eea%7C7bd08b0b33954dc194bbd0b2e56a497f%7C0%7C0%7C638587250841829161%7CUnknown%7CTWFpbGZsb3d8eyJWIjoiMC4wLjAwMDAiLCJQIjoiV2luMzIiLCJBTiI6Ik1haWwiLCJXVCI6Mn0%3D%7C0%7C%7C%7C&sdata=x7SgNeSGkGD67QB6AjTS013NdRefjMSEJhuMysqs%2B%2Bc%3D&reserved=0) requires authors to make all data underlying the findings described in their manuscript fully available without restriction, with rare exception (please refer to the Data Availability Statement in the manuscript PDF file). The data should be provided as part of the manuscript or its supporting information, or deposited to a public repository. For example, in addition to summary statistics, the data points behind means, medians and variance measures should be available. If there are restrictions on publicly sharing data—e.g. participant privacy or use of data from a third party—those must be specified.

Reviewer #1: Yes

Reviewer #2: No

>>We recognize that the underlying data for the survey are not available.  However, per IRB rules, we are not able to share the data because of risk of compromising respondent identities.

4. Is the manuscript presented in an intelligible fashion and written in standard English?

PLOS ONE does not copyedit accepted manuscripts, so the language in submitted articles must be clear, correct, and unambiguous. Any typographical or grammatical errors should be corrected at revision, so please note any specific errors here.

Reviewer #1: Yes

Reviewer #2: Yes

5. Review Comments to the Author

Please use the space provided to explain your answers to the questions above. You may also include additional comments for the author, including concerns about dual publication, research ethics, or publication ethics. (Please upload your review as an attachment if it exceeds 20,000 characters)

Reviewer #1:

1. This is a sound manuscript that adequately presents gaps and data that support the conclusions stated.
   a. Thank you
2. The title is informative and contains relevant and meaningful keywords.
   a. Thank you
3. The final sentence of lines 97-99 is missing a reference, but the introduction adequately summarizes the existing knowledge about the topic and gaps that exist in the literature.
   a. Thank you. We have added references to support this sentence.
4. Study aims are clear though the hypothesis could be framed more clearly.
   a. We feel that the question-based framing at the end of the introduction is most appropriate for this study because we did not have hypotheses about how community members were experiencing and interacting with NE CASC.
5. The methodology while mostly clear needs clarification. What methodology was used for the qualitative coding (line 202)?
   a. We followed grounded theory methodology to complete our coding. We have added more details to the qualitative analysis section of the results.
6. Also, please elaborate on why the Kruskal-Wallis rank sum test was used (line 204).
   a. We used the Kruskal-Wallis test to compare the numbers of responses in each category for the marginalized and non-marginalized groups. We used this non-parametric test to accommodate our small dataset which had an unequal distribution across groups. We have added clarification in this section of the methods.
7. The results and discussion were clear and all figures appeared accurate and were appropriately descriptive.
   a. Thank you

Reviewer #2: This study provided DEI insights in an academic institution setting. A survey was conducted with the Northeast Climate Adaptation Science Center consortium institutions to understand challenges faced by their community members, especially those from underrepresented groups. The study includes some interesting results related to the different motivations presented in the marginalized and non-marginalized groups. The paper can benefit from improvements in the following areas:

1. The introduction section only introduced the importance of understanding diversity in scientific and academic spaces, but failed to demonstrate the knowledge gaps in the area, and how this current study fills in these knowledge gaps. A literature review of similar previous studies in understanding DEI issues in scientific settings is completely missing. What approach has been taken by previous studies in understanding DEI issues, and what did they find as barriers to retention of scientists from historically marginalized backgrounds?
   a. We focused on describing study results in our introduction rather than comparing methodologies. We have included an additional paragraph (paragraph 8) in the introduction to clarify the methods for some of the studies we cited earlier in the introduction. We have discussed barriers to retention in paragraph 6 and 7 of the introduction. We have also discussed these barriers in the context of our results in the discussion. Other surveys have been campus wide, undergraduate focused, or conducted across many STEM disciplines. Our study is unique in three ways: 1) focus on a smaller community with similar research goals and 2) consideration of different career levels, 3) focusing on marginalized communities with an intersectional lens.
2. The introduction also seems to misalign with the actual research conducted. The latter has an emphasis of career stage and age diversity in addition to race and gender diversity, which was mainly discussed in the introduction section. Similarly, the “barriers to retention of scientists from historically marginalized backgrounds” mentioned as a goal in the introduction section isn’t really addressed by this research.
   a. We agree that the introduction focuses on race and gender diversity - this was intentional given that our results show that experiences of NE CASC community members primarily differed based on whether respondents identified as part of a marginalized group or whether they identified as cis-women.  In the former case, people from marginalized groups tended to feel less supported and were more likely to have considered leaving science.  In the latter case, cis-women also tended to feel less supported.  Because we did not see consistent differences based on age or career stage, we elected not to frame the introduction based on these identifiers.
      i. Our interpretation of the results related to whether respondents feel accepted and supported in the NE CASC community (Fig 2) and in science more generally (Fig 3) is that these feelings of inclusion are directly related to retention of people from historically marginalized groups. We have added a paragraph to the introduction to better connect feelings of support and belonging to retention of scientists in STEM.
3. Was the survey able to engage any of the tribal members involved in the Center? What specific efforts have been made to engage tribal members in the survey, especially given the Center’s emphasis on regional tribal nations?
   a. One participant at the center identified as Indigenous American (as reported in the demographic section). We do not have demographic information for all people affiliated with the center, so we do not know how many Indigenous people did not participate. For this survey, we aimed to engage all members of the center equally to better understand experiences of the NE CASC community from all backgrounds. Much of the past and ongoing Tribal engagement work involves working with Tribal Nations and organizations whose members are not part of the NE CASC community (i.e. not directly supported with funding from the Center).  However, partner organizations were not included in the survey.
4. What kinds of DEI initiatives have already been carried out by the Center? What is the Center’s vision related to DEI? What motivated the survey? This kind of background is important for the readers to understand the context of and the responses to the survey.
   a. This survey was among the initial DEI initiatives conducted by the Center. The survey was conducted in spring 2021 and the initial funding of ‘DEI Fellows’ began in fall 2020.  The survey was motivated by the online workshop held in December 2020, where a discussion among community members revealed that baseline data about demographics and experiences in science and at NE CASC were necessary to evaluate whether future DEI actions had any effect.  We have added background text on NE CASC’s DEI activities and motivation to the beginning of the survey development and implementation section.
5. Figure 1: This figure should include breakdowns in terms of age and career stage, as these are important groups being discussed in the subsequent analyses. Similarly, the gender figure needs to have breakdowns of cis-male and female.
   a. Thanks for this suggestion.  We have expanded Figure 1 to include the breakdowns of age, career stage, and gender.
6. What constituents disabled? Physical or mental? This is not clear.
   a. We included all types of disabilities in this category. The question in the survey was: “Please indicate any disability status that you may have” and the answers were:  “Yes I have a disability” and “No I do not have a disability”. We have included a clarifying sentence in the Demographic Information section of the Methods.
7. The narrative in the results section repeats the information that has already been presented in the figures. It is more important to discuss the analysis and the implications in the results, rather than simply restating the figures.
   a. While some results text and figures might be redundant, we prefer to keep the results text because it allows us to include all of the p values associated with the Kruskal-Wallis tests. The format of Plos ONE has a separate results and discussion section, so we have discussed the analysis and implications in the discussion section.
8. What are the implications of the differences in terms of motivation for pursuing climate adaptation science? How does it relate to the retention of the underrepresented climate scientists?
   a. Scientists from marginalized groups who have social motivations for their climate adaptation research may leave the field if they do not have the resources or support to do that research or if the field does not recognize the importance of social motivations in climate adaptation research. Other research (Schusler et al. 2021) has demonstrated that differences in motivations may contribute to students in environmental science feeling unwelcome and potentially leaving the field. Based on our results, this could also happen for researchers at later stages in their careers. Additionally, traditional funding mechanisms may also reduce opportunities to conduct research with social implications and motivations. We have revised this paragraph in the discussion to make the connections clearer.
9. How does the support for community members influence retention? What are the underlying reasons for the marginalized groups to feel less supported?
   a. Researchers who identified as marginalized were more likely to feel less supported than their non-marginalized peers and also more likely to have considered leaving academia in the last year. While we cannot derive causation, lack of support may be related to considering leaving the field. To make this connection clearer, we have revised the paragraph that starts:  “Discomfort and distrust, in addition to systemic inequities, may be driving marginalized scientists out of the field.” Additionally, within our own study we do not have long term data to track whether participants left CASC or the field and the survey was anonymous.
   b. We have discussed some of the underlying reasons that scientists may not feel supported in our discussion and literature review. For example, if they feel their motivations for research do not align with department goals. Our survey goal was to assess how community members felt at the center. Some of the underlying issues are related to systemic problems in academia and science, which we have discussed in both the introduction and discussion.
10. What does “commitment to DEI” entail? What kind of commitment already exists? This is the part that really needs some background information about the institutional context of the NE CASC from the DEI perspective.
    a. This is a good question - respondents might have interpreted this question in different ways.  Based on open ended responses, it is likely that commitment to DEI was often interpreted as support for activities that build inclusivity, support for trainings and other opportunities that help people learn about diverse perspectives on science, and a long-term commitment to continue to support such activities.  We added text to the introduction (in response to comment 4) to better describe the DEI activities supported by NE CASC leading up to this survey - these activities were focused on funding for ‘DEI Fellows’, whose efforts led to this survey and many years of Tribal engagement.  NE CASC had written a response condemning the murder of George Floyd, but did not have another stated commitment to DEI prior to this survey.  We have added some interpretation of ‘commitment to DEI’ to the discussion.
11. “Funding undergraduate students” is hard to understand again without the context. Is there a high percentage of unpaid undergraduate researchers within NE CASC currently? If so, what are the reasons for these students not to be compensated?
    a. We have added additional context in the discussion paragraph that starts “Scientists from marginalized groups ranked funding for undergraduate students as a top priority, while scientists from non-marginalized groups ranked funding for undergraduate students as their lowest priority and ranked DEI policy as their top priority.” Unpaid undergraduate opportunities are prevalent in environmental and natural sciences due to funding limitations and the perception among some researchers that they are a ‘rite of passage’. Recruiting diverse undergraduate students contributes to a more diverse workforce and can be difficult when many unpaid opportunities exclude diverse researchers. We have rearranged the paragraph and added more context to explain this issue.

USGS Reviewer Comments and responses:

I very much enjoyed the opportunity to read this manuscript and provide some comments.  I would definitely recommend the manuscript for publication, were I reviewing it on behalf of a journal.  It seems to me that the manuscript identifies a crucial limitation of the utilization of surveys in the DEIA space, not just where there is not a great deal of diversity in the survey pool, but also where there are small number of persons and anonymity becomes impossible to maintain.

I found the findings to be quite timely and relevant.  The recommendation to allow participants to self-identify as marginalized is a valuable one, and I hope that it will be replicated.

Thank you! We are grateful for your review and consideration of our manuscript.

The recommendations/comments that I have are as follows:

1. I think the article might benefit for a more in-depth discussion of limitations.  For example, the survey was not given to any undergraduate students, nor was it given to non-PIs on funded projects.  I would wonder if responses might be at all different among those researchers who were not the PIs.
   a. We provided the survey to all individuals who were formally associated with the NE CASC and its activities. Our study focused on graduate students, postdocs, and faculty to evaluate department dynamics in the CASC. Undergraduate students are not directly part of the NE CASC, which we have clarified in our methods section. Additionally, undergraduate experiences were not the focus of this study. There are already many studies on STEM experiences and ecology/environmental science experiences that are limited to undergraduate students [(Schusler et al., 2021; Taylor, 2018b, 2018c)](https://www.zotero.org/google-docs/?DJg3IX).
   b. As noted, we provided the survey to all individuals who were formally associated with the NE CASC and its programming. We did not intentionally exclude non-PIs on funded projects. In fact, several graduate students and postdocs were non-PIs on funded projects, which was why they were a part of the CASC environment. We did wonder if PIs and non-PIs would have different experiences, this was one reason we made comparisons between fellows/non-fellows. We have clarified this in the methods section. We have added more about this in our introduction. We have also added some discussion of the limitations of using the “marginalized respondents” category to the discussion.
2. Additionally, it is unclear to what extent the self-designation as marginalized overlapped with the demographic characteristics that are typically classed as marginalized.
   a. Yes. We have intentionally not made this comparison explicitly to preserve privacy of participants in accordance with our IRB; however, we have listed specific demographic information alongside the number of self-identified marginalized participants in Figure 1 and in the first paragraph of the demographics section. The answer to this question depends on which demographic one is considering (disability status, gender, sexuality, race, ethnicity, first generation college status, immigration status, socioeconomic background). Part of the goal of our study was to demonstrate that marginalized identities are intersectional and using singular demographic categories may miss the true experiences of community members, which we have discussed in depth in the discussion. For example, women have historically been marginalized in STEM, but while 22 cisgender women replied to the survey, only 10 people identified as historically marginalized. On the other hand, two individuals identified as having a disability (another group that would be historically marginalized in STEM). Additionally, because of the low numbers of survey respondents, we were not allowed to look at the specific breakdowns to preserve respondent privacy. They were collated for us by an outside party.
3. Finally, it is unclear why ten respondents chose not to answer the question regarding if they self-identified as marginalized.  This is almost a quarter of the respondents, and just as many as those who had identified as marginalized.
   a. We can only speculate about why these ten respondents did not complete this question in the survey. It is possible that they did not understand or skipped the question accidentally. Demographic questions were at the end of the survey, so it is possible that some respondents closed the survey before completing it. Additionally, we allowed respondents to check “yes” or “no” and some respondents may not have had strong feelings about where they fit. We have added this limitation to the discussion.
4. The article points to the need to address systemic barriers, but it doesn’t really indicate how an entity such as NECASC could do so.  I know that we have limitations on the ability to prescribe policy solutions, but it does seem that the problem is larger than the span of control of an organization such as NECASC.  Are there recommendations that can be made that address these systemic challenges? Are some of those within the sphere of influence of the CASC?  For example, there is explicit discussion of the need to have ecology curriculums not be blind to the harms that were perpetuated by some of the “great men” of the field.  Similarly, the article points to the potential for additional barriers for students who, for example, cannot afford to take unpaid or underpaid work.  Additional funding for undergraduate programs is mentioned as a potential solution, but perhaps a clearer recommendations section would be beneficial?
   a. Addressing systemic challenges may be beyond the scope of this paper and survey. However, we have included an additional paragraph at the end of the discussion to highlight how the NE CASC has considered survey results and taken action. Our goal was to highlight the voices of the scientists who identified as marginalized. As you note, we have highlighted many of these recommendations in the discussion along with justifications based on our results. Previously the recommendations were at the end of each paragraph and may have been missed. Instead of creating a recommendations section, we have moved the recommendations to the beginning of the paragraphs to make them stand out more and allow them to stay with their justifications.
